# Synthesis of diamido-bridged bis-pillar[5]arenes and tris-pillar[5]arenes for construction of unique [1]rotaxanes and bis-[1]rotaxanes

**DOI:** 10.3762/bjoc.14.142

**Published:** 2018-07-04

**Authors:** Ying Han, Li-Ming Xu, Cui-Yun Nie, Shuo Jiang, Jing Sun, Chao-Guo Yan

**Affiliations:** 1 College of Chemistry & Chemical Engineering, Yangzhou University, Yangzhou 225002, P. R. China

**Keywords:** bis-[1]rotaxane, mechanically interlocked molecule, pillar[5]arene, [1]rotaxane, self-assembly

## Abstract

The pillar[5]arene mono- and di(oxyalkoxy)benzoic acids were successfully prepared in high yields by sequential alkylation of ω-bromoalkoxy-substituted pillar[5]arenes with methyl or ethyl *p*-hydroxybenzoate followed by a hydrolytic reaction under basic conditions. Under catalysis of HOBt/EDCl, the amidation reaction of pillar[5]arene mono(oxybutoxy)benzoic acid with monoamido-functionalized pillar[5]arenes afforded diamido-bridged bis-pillar[5]arenes. ^1^H NMR and 2D NOESY spectra clearly indicated that [1]rotaxanes were formed by insertion of longer diaminoalkylene unit into the cavity of one pillar[5]arene with another pillar[5]arene acting as a stopper. The similar catalysed amidation reaction of pillar[5]arene di(oxybutoxy)benzoic acid with monoamido-functionalized pillar[5]arenes resulted in the diamido-bridged tris-pillar[5]arenes, which successfully form the unique bis-[1]rotaxanes bearing longer than diaminopropylene diamido bridges.

## Introduction

The construction and dynamic motion of the mechanically interlocked molecules (MIMs) have attracted significant research interests due to their intrinsic self-assembled nature and potential applications in various aspects [1–4]. Pseudo[1]rotaxane and [1]rotaxane are one of particular supramolecular assembly system and are considered as an important building block in the construction of diverse MIMs [5–10]. [1]Rotaxane has a macrocyclic wheel component connected with a self-locked chain axle, and a bulky stopper at the terminal axle to prevent dissociation of the subcomponents. In recent years, many effects have been devoted to the construction and functionalization of pseudo[1]rotaxanes and [1]rotaxanes [11–20]. For this purpose, the well-known macrocycles such as crown ether [21–23], cyclodextrin [24–26], calixarene [27–29] and pillararene have been successfully employed as the wheel subcomponent. Pillararenes are new star macrocyclic compounds with aromatic rings *para*-bridged by methylene units and have unique tubular shape rather than cone [30–32]. In recent years, an explosive development on the construction of various supramolecular devices and diverse responsive materials has been reported by using diverse functionalized pillararenes [33–35]. Due to easily preparation and suitable cavity, functionalized pillar[5]arenes were widely used as wheel component for constructing of the various interlocked molecules [36–42]. In the past few years, many elegant works on the construction of pseudo[1]rotaxanes and [1]rotaxanes have been developed on the basis of various mono-functionalized pillar[5]arenes [43–57]. Recently, we have successfully constructed a couple of pseudo[1]rotaxane and [1]rotaxane both in solution and in solid state developed by using mono-functionalized pillar[5]arene Schiff base, urea and pyridylimine derivatives [58–63]. In continuation of our effort on the development on the construction of [1]rotaxanes via various mono-functionalized pillar[5]arene derivatives, herein we wish to report the convenient synthesis of diamido-bridged bis-pillar[5]arenes and tris-pillar[5]arenes as well as formation of unique [1]rotaxanes and bis-[1]rotaxanes.

## Results and Discussion

**Scheme 1:**
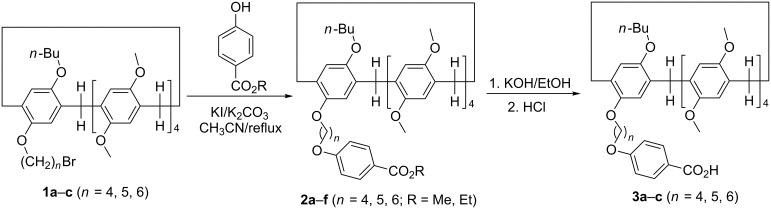
Synthesis of pillar[5]arene mono(oxyalkoxy)benzoic acids **3a**–**c**.

**Figure 1:**
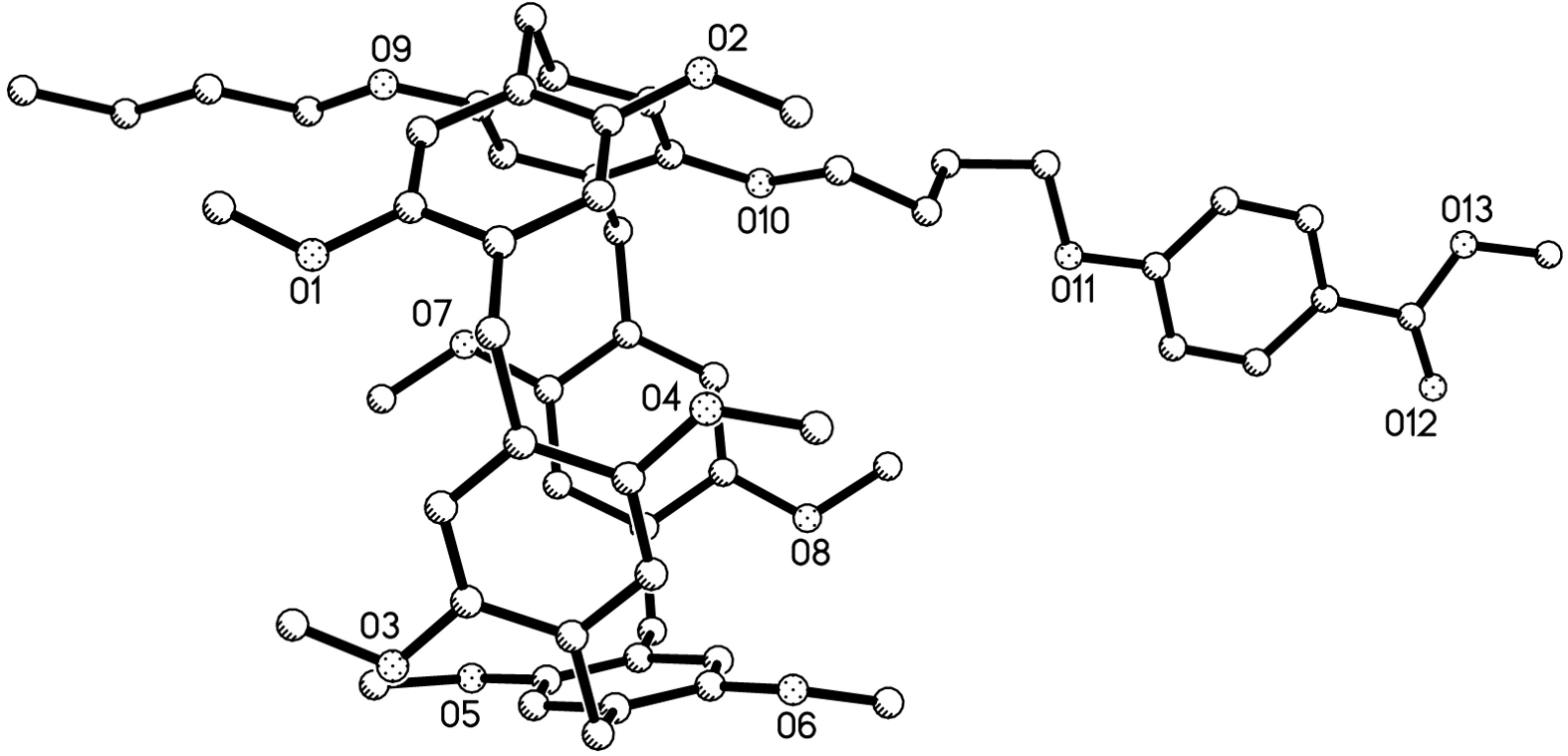
single crystal structure of pillar[5]arene **2a**.

**Figure 2:**
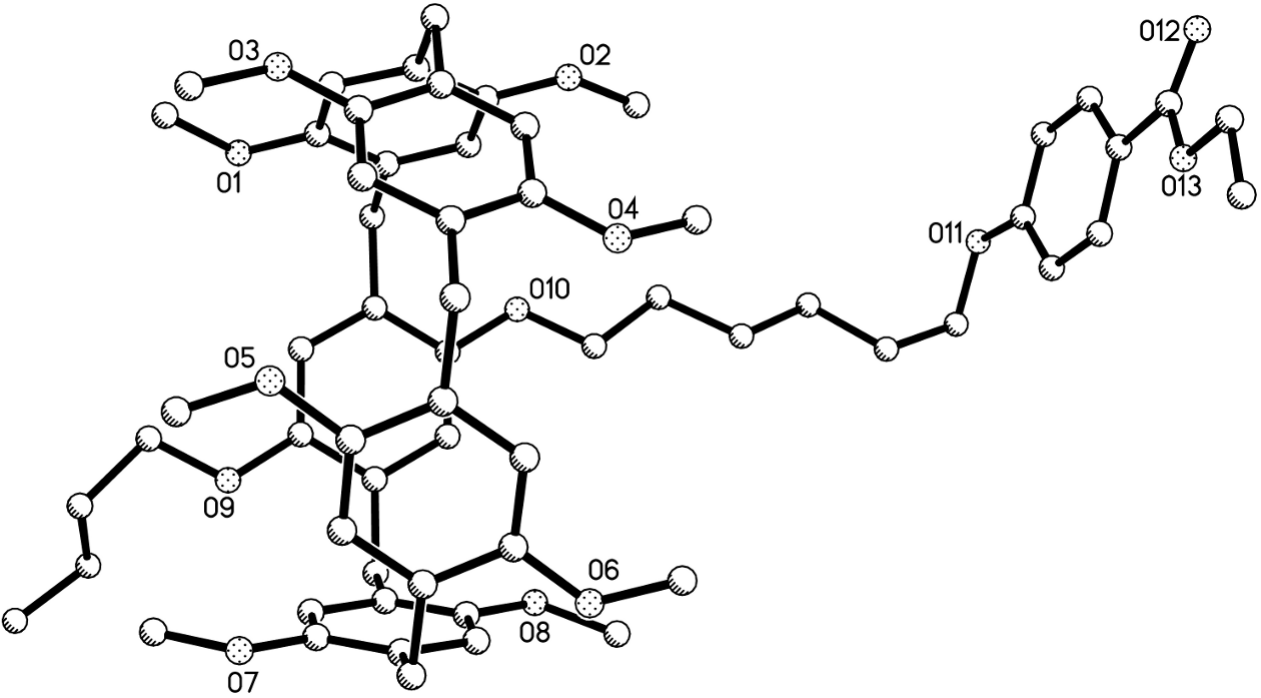
Single crystal structure of pillar[5]arene **2f**.

The synthetic route for the pillar[5]arene mono(oxyalkoxy)benzoic acids was illustrated in Scheme 1. Firstly, the alkylation of mono(bromoalkoxy)pillar[5]arene **1a**–**c** (*n* = 4, 5, 6) [64] with methyl or ethyl *p*-hydroxybenzoate was carried out in the refluxed medium of KI/K_2_CO_3_/CH_3_CN for one day. The pillar[5]arene mono(oxyalkoxy)benzoates **2a**–**f** were successfully prepared in high yields. Then, basic hydrolysis of pillar[5]arene mono(oxyalkoxy)benzoates **2a**–**f** in ethanol in the presence of potassium hydroxide afforded the desired pillar[5]arene mono(oxyalkoxy)benzoic acids **3a**–**c**. The structures of the prepared pillar[5]arenes **2a**–**f** and **3a**–**c** were fully characterized by the spectroscopic methods. The single crystal structures of the pillar[5]arenes **2a** (Figure 1), **2c**, **2d**, **2e** (Supporting Information File 1, Figure S1–S3) and **2f** (Figure 2) were successfully determined by X-ray diffraction. The same structural feature was obtained in the five single crystals. That is, the longer chain of methyl (ethyl) oxyalkoxybenzoate not only does not inserted in the cavity of the pillar[5]arene to form the pseudo[1]rotaxane, but also does not thread to the cavity of the neighbouring pillar[5]arene to form the supramolecular polymer. This result is consistent to the Cao’s previously reported results in the series of pillar[5]arenes bearing aliphatic esters [49], in that they found the chain of methyl oxybutyrate did not threaded into the cavity of pillar[5]arene.

**Scheme 2:**
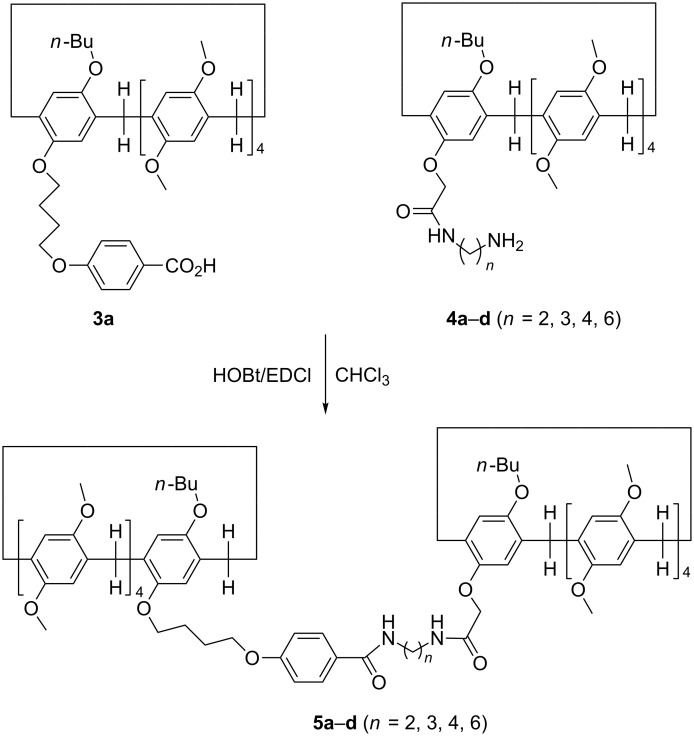
Synthesis of diamido-bridged bis-pillar[5]arenes **5a**–**d**.

**Figure 3:**
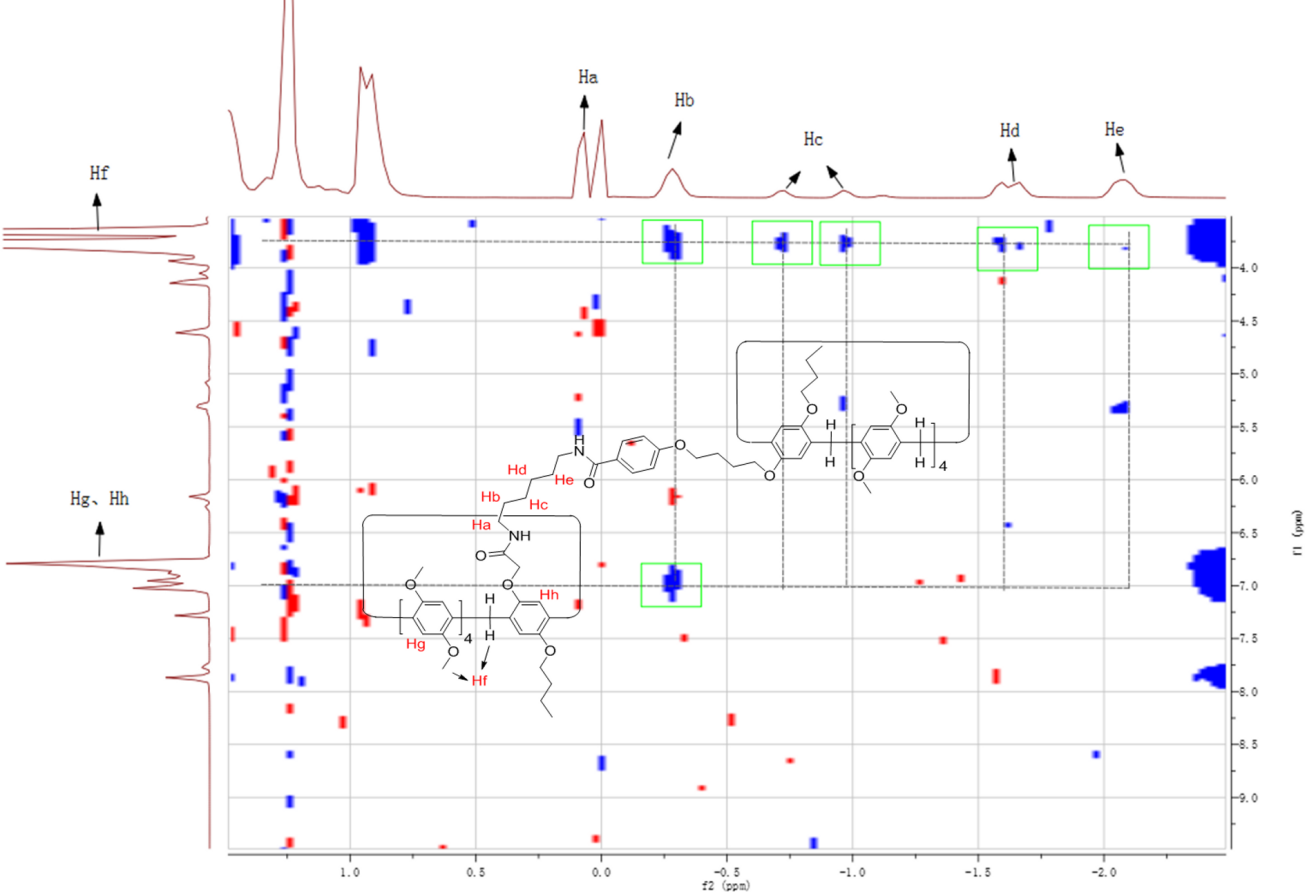
The 2D NOSEY spectrum of bis-pillar[5]arene **5d**.

The above synthetic pillar[5]arene mono(oxyalkoxy)benzoic acids have a longer chain functionalized group and a large macrocycle, which enabled them to be a good candidate as an efficient terminal stopper for the construction of rotaxanes. Therefore, the amidation reaction of pillar[5]arene mono(oxybutoxy)benzoic acid **3a** with our previously reported amido-functionalized pillar[5]arenes **4a**–**d** (*n* = 2, 3, 4, 6) [58] was carried out in chloroform under the combined catalysis of 1-hydroxybenzotrizole (HOBt) and 1-(3-dimethylaminopropyl)-3-ethylcarbodiimide hydrochloride (EDCl). The reaction proceeded smoothly to give diamido-bridged bis-pillar[5]arenes **5a**–**d** (*n* = 2, 3, 4, 6) in moderate yields (Scheme 2). It has been reported that the chain of *N*-(ω-aminoalkyl)oxyacetamide inserted in the cavity of pillar[5]arene in the amido-functionalized pillar[5]arene **4a**–**d** (*n* = 2, 3, 4, 6) to form pseudo[1]rotaxanes both in solution and in solid state [58]. The diamido-bridged bis-pillar[5]arenes **5a**–**d** might form the expected [1]rotaxanes. ^1^H NMR spectrum of the bis-pillar[5]arenes **5a** clearly showed that there is no any signals at very high magnetic field (δ < 0), which indicated that the diaminoethylene chain does not inserted in the cavity of pillar[5]arene to form the expected [1]rotaxane. Therefore, the two moieties of pillar[5]arenes are just connected by the diaminoethylene chain from the outside in diamido-bridged bis-pillar[5]arenes **5a**. However, a couple of characteristic signals at very high magnetic field can be seen in the ^1^H NMR spectra of the bis-pillar[5]arenes **5b**–**d**. There is a broad singlet at −1.82 ppm in **5b**, a mixed peak at −1.88 to −2.14 ppm in **5c** and several peaks in the range of 0.07 to −2.07 ppm in **5d**. This result clearly displayed that the unique [1]rotaxane structures were actually formed by threading the longer diaminoalkylene bridge in the cavity of one molecular pillar[5]arene, while another pillar[5]arene as the bigger stopper. Additionally, 2D NOESY spectra of the compound **5d** provided more strong evidence for the formation of [1]rotaxane (Figure 3). The NOE correlations were clearly observed between Ha, Hb, Hc, Hd, and He protons of the bridging hexylene chain with the proton Hf in the core of pillar[5]arene. The proton Hb of the bridging hexylene chain also correlated with protons of the aromatic protons Hg and Hf.

**Scheme 3:**
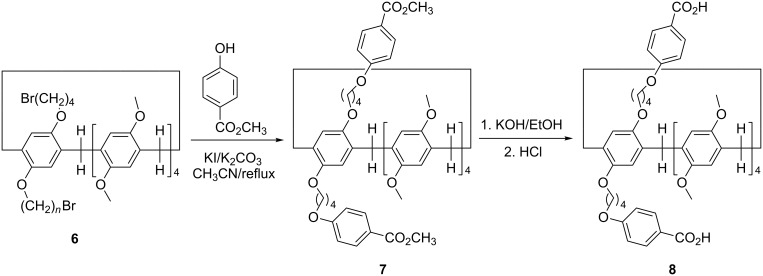
Synthesis of pillar[5]arene di(oxyalkoxy)benzoic acid **8**.

**Figure 4:**
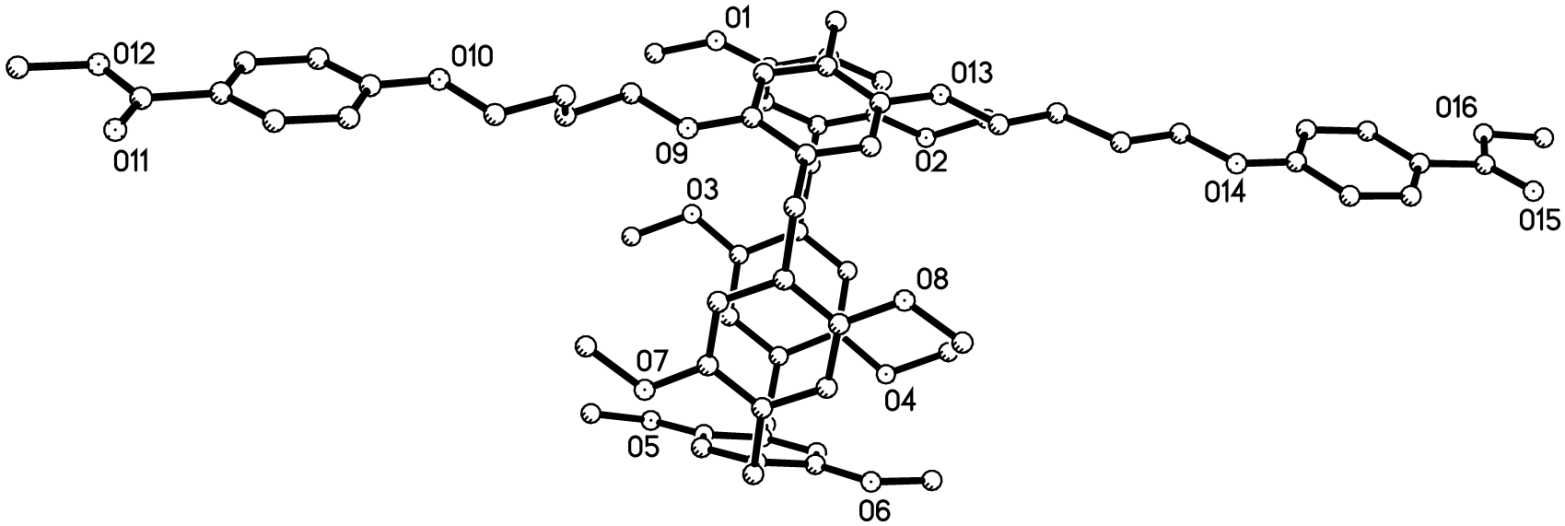
Single crystal structure of pillar[5]arene **7**.

According to similar reaction procedure for the synthesis of pillar[5]arene mono(oxyalkoxy)benzoic acids **3a**–**c**, pillar[5]arene di(oxybutoxy)benzoic acid **8** was prepared in moderate yield from sequential alkylation and basic hydrolysis reaction (Scheme 3). The single crystal structure of the pillar[5]arene di(oxybutoxy)benzoate **7** showed that the two chains of methyl oxybutoxybenzoate did not insert in the cavity of pillar[5]arene (Figure 4) as that of the above mentioned pillar[5]arene mono(oxybutoxy)benzoates **2a**–**f**. The two chains straight stretched to the opposite direction of central pillar[5]arene. It might be attribute to the electron-rich effect of the methyl oxybutoxybenzoate unit, which kept it away from the electron-rich cavity of pillar[5]arene.

**Scheme 4:**
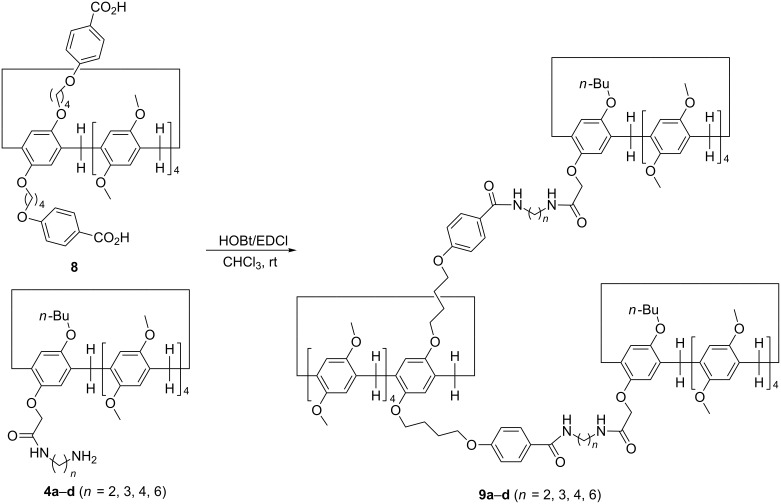
Synthesis of diamido-bridged tris-pillar[5]arenes **9a**–**d**.

Under the combined catalysis of HOBT and EDCl, the amide reaction of pillar[5]arene di(oxybutoxy)benzoic acid **8a** with two molecular amido-functionalized pillar[5]arenes **4a**–**d** in chloroform afforded tris-pillar[5]arenes **9a**–**d** in moderate yields (Scheme 4). The structures of the synthetic tris-pillar[5]arenes **9a**–**d** were fully characterized by IR, HRMS, ^1^H and ^13^C NMR spectra. The ^1^H NMR spectra provided stronger evidence for the formation of fascinating bis-[1]rotaxanes. Because there are no peaks with negative chemical shift in the ^1^H NMR spectra of the tris-pillar[5]arene **9a**, it can be concluded that the three pillar[5]arenes are connected from the outsides by two diamidoethylene-bridges. There is one broad peak at −1.80 ppm in tris-pillar[5]arene **9b**, a mixed peak at −2.00 ppm in tris-pillar[5]arene **9c**, and five broad peaks at −0.29 ppm, −0.74 ppm, −0.97 ppm, −1.62 ppm and −2.08 ppm in tris-pillar[5]arene **9d**. Therefore, ^1^H NMR spectra of **9b**–**d** indicated that the diaminoalkylene chain ambiguously inserted in the cavity of the pillar[5]arene. In other words, the fascinating bis-[1]rotaxane structures were formed in the tris-pillar[5]arenes **9b**–**d**. Here, the lengths of bridging chains played the critical role in the selflocked behaviour of pillar[5]arene-based [1]rotaxanes.

**Figure 5:**
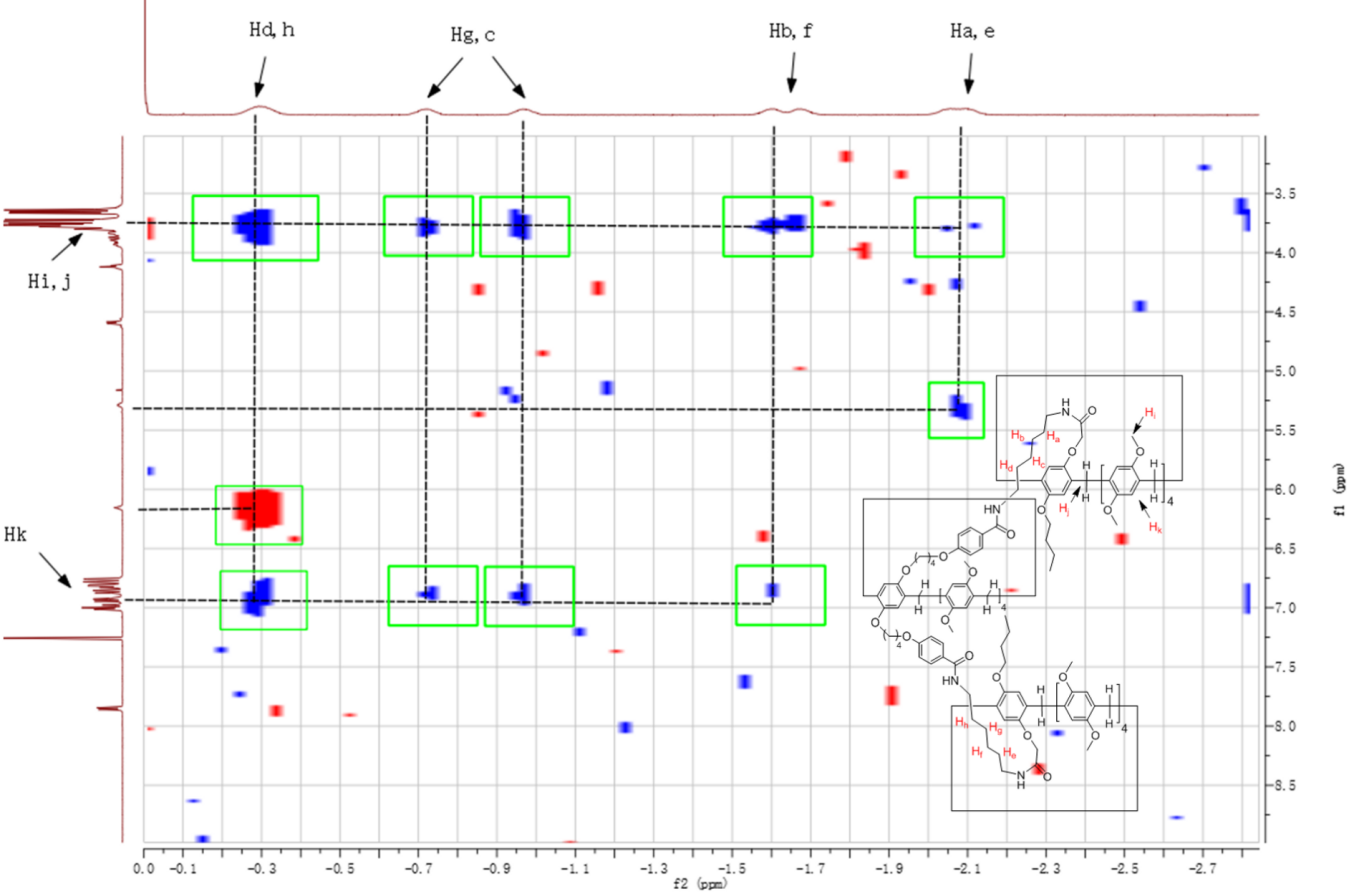
The 2D NOSEY spectra of tris-pillar[5]arene **9d**.

In order to confirm the formation of the bis-[1]rotaxanes, 2D NOESY spectra of the compounds **9a**–**d** were recorded. The 2D NOESY spectrum of compound **9d** was showed in Figure 5. There it can be seen that the NOE correlations were clearly observed between Ha, Hb, Hc, Hd, Hf, Hg, Hh protons of the bridging diaminohexylene chain and the protons Hi, Hj in the core of pillar[5]arene. Additionally, some correlations exists between protons Ha, He, Hd and Hh and active amino (N–H) group. These NOE correlations clearly indicated the two bridged diaminohexylene chain threading into the cavity of the two pillar[5]arenes to form the bis-[1]rotaxane. The similar correlations were also observed in the NOESY spectra of the tris-pillar[5]arene **9b** and **9c** (see Supporting Information File 1, Figures S5 and S6). However, there is no such correlation in the 2D NOESY spectrum of the compound **9a** (see Supporting Information File 1, Figure S4), which confirmed that the diamidoethylene bridge did not insert to the cavity of the pillar[5]arene to form [1]rotaxanes. Thus, the 2D NOESY spectra provided stronger evidence for the formation of novel bis-[1]rotaxanes for the tris-pillar[5]arenes **9c**–**d** bearing longer than diaminopropylene diamido-bridges.

## Conclusion

In summary, we have conveniently prepared several pillar[5]arene mono- and di(oxyalkoxy)benzoic acids and found that the chain of alkyl oxyalkoxybenzoate did not inserted to the cavity of pillar[5]arene. More importantly, a series of diamido-bridged bis-pillar[5]arenes and tris-pillar[5]arenes were efficiently synthesized by catalyzed amidation reaction of pillar[5]arene mono- and di(oxybutoxy)benzoic acids with monoamide-functionalized pillar[5]arenes. On the basis of ^1^H NMR and 2D NOESY spectra, we successfully concluded that the chains longer than diaminopropylene threaded into the one or two cavities of the pillar[5]arenes to form the unique [1]rotaxane and bis-[1]rotaxanes. This work not only provided a fundamental self-assembly of the mechanically interlocked molecules, but also developed the potential applications of pillar[5]arene in supramolecular chemistry. The design and construction of diverse mechanically interlocked molecules are underway in our laboratory.

## Supporting Information

Experimental procedures, analytical data, and copies of the ^1^H and ^13^C NMR spectra, HRMS spectra for all new products. Single crystal data for **2a** (CCDC: 1837205), **2c** (CCDC: 1837206), **2d** (CCDC: 1837207), **2e** (CCDC: 1837208), **2f** (CCDC: 1837209) and **7** (CCDC: 1846692) have been deposited at the Cambridge Crystallographic Data Centre.

File 1 Experimental and analytical data.
