# Supplementary material for: Synthesis of diamido-bridged bis-pillar[5]arenes and tris-pillar[5]arenes for construction of unique [1]rotaxanes and bis-[1]rotaxanes
Source: Beilstein J Org Chem. 2018 Jul 4;14:1660–7. doi: 10.3762/bjoc.14.142 (PMC6036973; doi:10.3762/bjoc.14.142)
Supplement: File 1 — Experimental and analytical data. [file Beilstein_J_Org_Chem-14-1660-s001.pdf]

Supporting Information

Synthesis of diamido-bridged bis-pillar[5]arenes and tris-pillar[5]arenes for construction of unique [1]rotaxanes and bis-[1]rotaxanes

Ying Han, Li -Ming Xu, Cui-Yun Nie, Shuo Jiang, Jing Sun and Chao-Guo Yan*

Address: College of Chemistry & Chemical Engineering, Yangzhou University, Yangzhou 225002, P. R. China

Email: Chao-Guo Yan - cgyan@yzu.edu.cn

*Corresponding author

Experimental and analytical data

Experimental procedures and characterization data S2–S10

Figures of single crystal structures S1-S3 S11

2D NOESY spectra S12–S14

Characterization data and ^{1}H NMR, ^{13}C NMR, HRMS spectra S15–S52

Experimental procedures and characterization data

Materials and methods

All reactions were performed in open atmosphere unless otherwise stated. All reagents, unless otherwise indicated, were obtained from commercial sources. Melting points (m.p.) were determined using a Focus X-4 apparatus and were not corrected. All yields were given as isolated yields. NMR spectra were recorded on a Bruker DPX 400 MHz or 600 MHz spectrometer with internal standard tetramethylsilane (TMS) and solvent signals as internal references, and the chemical shifts (δ) were expressed in ppm and *J* values were given in Hz. 2D NOESY experiments were performed on a Bruker DPX 600 MHz spectrometer. High-resolution ionization mass spectra (HR-ESI-MS) were recorded on Trace DSQ XaMis LCMS equipped with an electrospray ionization (ESI) probe operating in positive-ion mode with direct infusion. The starting materials mono-(ω-bromoalkyl)pillar[5]arenes **1a–c** [1], amido-functionalized pillar[5]arenes **4a–d** [2], and di-(ω-bromobutyl)pillar[5]arenes **6** [1] were prepared according to the published methods.

[1] Huo, G. F.; Han, Y.; Sun, J.; Yan, C. G. *J Incl Phenom Macrocycl Chem* **2016**, *86*, 231-240.

[2] Han, Y.; Huo, G. F.; Sun, J.; Xie, J.; Yan, C. G.; Zhao, Y.; Wu, X.; Lin, C.; Wang, L. Y. *Sci. Reports* **2016**, *6*, 28748.

1. General procedure for the synthesis of pillar[5]arene mono-oxyalkoxybenzoates 2a–f: A mixture of mono-(ω-bromoalkyl)pillar[5]arene **1a–c** (1.0 mmol), methyl or ethyl 4-hydroxybenzoate (1.2 mmol), potassium iodide (0.5 g) and potassium carbonate (40.0 mmol, 5.50 g) in dry acetonitrile (50.0 mL) was refluxed for one day. After cooling, the solid was removed by filtration and the solvent was removed by evaporation at reduced pressure. The resulting crude product was recrystallized from ethanol to give the pure product.

2a: White solid, 80%, m.p. 88-90 °C; ^{1}H NMR (400 MHz, $CDCl_3$) δ: 7.98 (d, *J* = 4.4 Hz, 2H, ArH), 6.88 (d, *J* = 4.8 Hz, 2H, ArH), 6.80-6.77 (m, 10H, ArH), 4.01-4.00 (m, 2H, CH_2), 3.92-3.91 (m, 2H, CH_2), 3.89 (s, 3H, OCH_3), 3.84 (t, *J* = 6.4 Hz, 2H, CH_2), 3.77-3.76 (m, 10H, CH_2), 3.68-3.64 (m, 24H, OCH_3), 1.96 (brs, 4H, CH_2), 1.78-1.72 (m, 2H, CH_2), 1.54-1.47 (m, 2H, CH_2), 0.95 (t, *J* = 7.2 Hz, 3H, CH_3), ^{13}C NMR (100 MHz, $CDCl_3$) δ:166.8, 162.7, 150.7, 150.7, 150.7, 150.7, 150.6, 150.1, 149.7, 131.5, 128.3, 128.3, 128.1, 122.4, 114.9, 114.1, 114.0, 114.0, 114.0, 113.9, 113.9, 68.1, 67.8, 67.6, 60.5, 55.8, 55.7, 55.7, 55.7, 55.7, 55.6, 51.8, 31.8, 29.7, 29.6, 29.5,

26.2, 26.0, 19.4, 13.9, IR (KBr) υ:3579, 3414, 3052, 2944, 2862, 2835, 2750, 1928, 1713, 1660, 1610, 1503, 1460, 1400, 1260, 1211, 1177, 1105, 1045, 983, 931, 849, 772, 770 cm^{-1}, MS (m/z): HRMS (ESI) Calcd. for $C_{59}H_{69}O_{13}$ ($[M+H]^+$): 985.4738, found: 985.4749.

2b: White solid, 78%, m.p. 90-92 °C; ^1H NMR (400 MHz,$CDCl_3$) δ: 7.98 (d, *J* = 8.8 Hz, 2H, ArH), 6.88 (d, *J* = 8.8 Hz, 2H, ArH), 6.78-6.75 (m, 10H, ArH), 4.37-4.32 (m, 2H, CH_2), 4.01-4.00 (m, 2H, CH_2), 3.89 (t, *J* = 5.2 Hz, 2H, CH_2), 3.83 (t, *J* = 6.4 Hz, 2H, CH_2), 3.77-3.76 (m, 10H, CH_2), 3.65-3.60 (m, 24H, OCH_3), 1.94 (brs, 4H, CH_2), 1.77-1.70 (m, 2H, CH_2), 1.52-1.47 (m, 2H, CH_2), 1.38 (t, *J* = 7.2 Hz, 3H, CH_3), 0.94 (t, *J* = 7.6 Hz, 3H, CH_3), ^{13}C NMR (100 MHz, $CDCl_3$) δ:166.4, 162.7, 150.7, 150.6, 150.6, 150.6, 150.6, 150.1, 149.6, 131.5, 128.4, 128.4, 128.3, 128.3, 128.2, 122.7, 114.8, 114.0, 113.9, 113.8, 113.8, 68.1, 67.9, 67.6, 60.6, 55.8, 55.8, 55.7, 55.7, 55.6, 31.8, 29.6, 29.6, 29.5, 29.5, 26.3, 19.4, 14.3, 13.9, IR (KBr) υ:3462, 3051, 2944, 2863, 2835, 2750, 1934, 1708, 1607, 1503, 1461, 1400, 1361, 1211, 1177, 1104, 1044, 931, 850, 772, 701 cm^{-1}, MS (m/z):HRMS (ESI) Calcd. for $C_{60}H_{71}O_{13}$ ($[M+H]^+$): 999.4895, found: 999.4910.

2c: White solid, 81%, m.p. 99-101 °C; ^1H NMR (400 MHz, DMSO-d_6) δ: 7.90 (d, *J* = 8.4 Hz, 2H, ArH), 7.01 (d, *J* = 8.4 Hz, 2H, ArH), 6.77-6.75 (m, 10H, ArH), 4.04 (brs, 2H, CH_2), 3.86-3.81 (m, 7H, CH_2,OCH_3), 3.65-3.61 (m, 34H, CH_2, OCH_3), 1.77 (brs, 4H, CH_2), 1.69-1.59 (m, 4H, CH_2), 1.48-1.42 (m, 2H, CH_2), 0.90 (t, *J* = 7.6 Hz, 3H, CH_3), ^{13}C NMR (100 MHz, $CDCl_3$) δ: 166.8, 162.8, 150.7, 150.6, 150.6, 150.6, 150.0, 149.7, 131.4, 128.3, 128.1, 115.0, 114.8, 114.0, 68.1, 55.7, 51.8, 31.8, 29.6, 29.5, 29.4, 29.4, 19.4, 13.9, IR (KBr) υ: 3845, 3579, 3408, 3072, 2943, 2864, 2835, 2742, 1927, 1714, 1606, 1504, 1462, 1399, 1257, 1211, 1107, 1045, 930, 870, 850, 773, 701 cm^{-1}, MS (m/z): HRMS (ESI) Calcd. for $C_{60}H_{71}O_{13}$ ($[M+H]^+$): 999.4895, found: 999.4907

2d: White solid, 76%, m.p. 96-98 °C; ^1H NMR (400 MHz, DMSO-d_6) δ: 7.90 (d, *J* = 8.8 Hz, 2H, ArH), 7.01 (d, *J* = 8.8 Hz, 2H, ArH), 6.77-6.75 (m, 10H, ArH), 4.29-4.24 (m, 2H, CH_2), 4.05 (brs, 2H, CH_2), 3.85-3.80 (m, 4H, CH_2), 3.65-3.61 (m, 34H, CH_2, OCH_3), 1.78 (brs, 4H, CH_2), 1.69-1.64 (m, 4H, CH_2), 1.47-1.42 (m, 2H, CH_2), 1.29 (t, *J* = 7.2 Hz, 3H, CH_3), 0.90 (t, *J* = 7.6 Hz, 3H, CH_3), ^{13}C NMR (100 MHz, $CDCl_3$) δ: 166.4, 162.8, 162.8, 150.5, 150.4, 150.4, 149.9, 149.6, 131.4, 128.5, 128.4, 128.3, 116.6, 114.6, 114.0, 113.7, 113.7, 113.6, 113.5, 68.1, 67.9, 60.6, 57.8, 55.7, 55.7, 31.9, 29.5, 19.5, 17.5, 14.3, 14.0, IR (KBr) υ: 3578, 3051, 2942, 2865, 2835, 1708, 1606, 1503, 1462, 1399, 1310, 1259, 1211, 1172, 1105, 1045, 930, 870, 773, 702 cm^{-1}, MS (m/z):

HRMS (ESI) Calcd. for $C_{61}H_{73}O_{13}$ ([M+H]$^+$): 1013.5051, found: 1013.5056.

2e: White solid, 79%, m.p. 111-113 °C; ^{1}H NMR (400 MHz, $CDCl_3$) δ: 7.98 (d, *J* = 8.4 Hz, 2H, ArH), 6.85 (d, *J* = 8.4 Hz, 2H, ArH), 6.78-6.76 (m, 10H, ArH), 3.89 (s, 3H, OCH_3), 3.82 (brs, 6H, CH_2), 3.76 (brs, 10H, CH_2), 3.64-3.60 (m, 24H, OCH_3), 1.74 (brs, 4H, CH_2), 1.48-1.46 (m, 4H, CH_2), 1.38 (t, *J* = 7.2 Hz, 2H, CH_2), 1.25-1.24 (m, 2H, CH_2), 0.93 (t, *J* = 6.8 Hz, 3H, CH_3), ^{13}C NMR (100 MHz, $CDCl_3$) δ:167.0, 163.1, 150.7, 150.7, 150.6, 150.6, 150.6, 150.1, 131.4, 128.2, 128.2, 113.9, 68.1, 60.6, 55.9, 55.8, 55.7, 51.8, 31.8, 29.7, 19.3, 14.4, 13.9, IR (KBr) υ: 3656, 2942, 2862, 2836, 1714, 1606, 1503, 1462, 1399, 1261, 1211, 1174, 1104, 1044, 929, 850, 773, 701 cm^{-1}, MS (m/z): HRMS (ESI) Calcd. for $C_{61}H_{73}O_{13}$ ([M+H]$^+$): 1013.5051, found: 1013.5060.

2f: White solid, 78%, m.p. 96-98 °C; ^{1}H NMR (400 MHz, $CDCl_3$) δ: 7.98 (d, *J* = 8.8 Hz, 2H, ArH), 6.86 (d, *J* = 8.8 Hz, 2H, ArH), 6.79-6.73 (m, 10H, ArH), 4.37-4.32 (m, 2H, CH_2), 3.85-3.81 (m, 8H, CH_2), 3.77-3.76 (m, 10H, CH_2), 3.65-3.59 (m, 24H, OCH_3), 1.76-1.71 (m, 4H, CH_2), 1.62 (brs, 2H, CH_2), 1.50-1.47 (m, 4H, CH_2), 1.38 (t, *J* = 6.8 Hz, 3H, CH_3), 0.94 (t, *J* = 7.2 Hz, 3H, CH_3), ^{13}C NMR (100 MHz, $CDCl_3$) δ: 166.5, 163.3, 162.9, 150.7, 150.7, 150.7, 150.7, 150.1, 149.8, 131.3, 128.2, 114.9, 113.9, 68.2, 60.6, 55.8, 55.7, 31.8, 29.7, 19.3, 14.4, 13.9, IR (KBr) υ:3657, 2942, 2862, 2835, 1708, 1607, 1502, 1462, 1398, 1263, 1211, 1173, 1100, 928, 850, 773, 702 cm^{-1}, MS (m/z): HRMS (ESI) Calcd. for $C_{62}H_{75}O_{13}$ ([M+H]$^+$): 1027.5208, found: 1027.5216.

2. General procedure for the synthesis of pillar[5]arene oxyalkoxybenzoic acids (3a–c):

A mixture of pillar[5]arene oxyalkoxybenzoates **2a–f** (0.5 mmol) and potassium hydroxide (10.0 mmol, 0.56 g) in 95% ethanol was refluxed overnight. After cooling, the solution was neutralized with dilute hydrochloric acid to give white precipitates, which were washed with water to give the pure products **3a–c**.

3a: White solid, 81%, m.p. 100-102 °C; ^{1}H NMR (400 MHz, DMSO-d_6) δ: 12.65 (s, 1H, COOH), 7.88 (d, *J* = 8.8 Hz, 2H, ArH), 7.00 (d, *J* = 8.8 Hz, 2H, ArH), 6.79-6.75 (m, 10H, ArH), 4.12 (t, *J* = 6.0 Hz, 2H, CH_2), 3.90 (t, *J* = 6.0 Hz, 2H, CH_2), 3.82 (t, *J* = 6.4 Hz, 2H, CH_2), 3.65-3.64 (m, 34H, CH_2, OCH_3), 1.96-1.90 (m, 4H, CH_2), 1.71-1.64 (m, 2H, CH_2), 1.48-1.42 (m, 2H, CH_2), 0.90 (t, *J* = 7.2 Hz, 3H, CH_3), ^{13}C NMR (100 MHz, DMSO-d_6) δ: 167.6, 162.5, 150.3, 150.3, 150.2, 149.6, 149.4, 131.7, 128.1, 127.9, 127.9, 127.9, 127.9, 127.8, 114.5, 113.7, 113.7, 113.6, 67.9, 55.8, 55.7, 31.6, 29.5, 29.4, 26.2, 25.9, 19.3, 14.1, IR (KBr) υ: 3834, 3577, 3427, 2941, 2862, 2835, 2659, 1926, 1685, 1605, 1504, 1461, 1398, 1309, 1252, 1232, 1187, 1101, 1043, 931, 851, 775 cm^{-1}, MS

(m/z):HRMS (ESI) Calcd. for $C_{58}H_{67}O_{13}$ ($[M+H]^+$): 971.4582, found: 971.4587.

3b: White solid, 78%, m.p. 106-108 °C; ^{1}H NMR (400 MHz, DMSO-d_6) δ: 12.61 (s, 1H, COOH), 7.88 (d, *J* = 8.8 Hz, 2H, ArH), 7.00 (d, *J* = 8.8 Hz, 2H, ArH), 6.77-6.76 (m, 10H, ArH), 4.05 (t, *J* = 6.0 Hz, 2H, CH_2), 3.87-3.80 (m, 4H, CH_2,), 3.65-3.64 (m, 34H, CH_2, OCH_3), 3.82 (t, *J* = 6.4 Hz, 2H, CH_2), 3.65-3.61 (m, 34H, CH_2, OCH_3), 1.79 (t, *J* = 6.4 Hz, 4H, CH_2), 1.69-1.63 (m, 4H, CH_2), 1.48-1.42 (m, 2H, CH_2), 0.90 (t, *J* = 7.2 Hz, 3H, CH_3), ^{13}C NMR (100 MHz, DMSO-d_6) δ: 167.4, 162.6, 150.3, 150.3, 150.2, 150.2, 149.6, 131.7, 128.0, 128.0, 127.9, 127.8, 127.8, 114.5, 113.7, 113.7, 113.6, 113.6, 68.1, 68.0, 67.8, 55.8, 55.7, 55.7, 31.6, 29.3, 22.7, 19.3, 14.2,δ: IR (KBr) υ: 3656, 2940, 2862, 2835, 1685, 1605, 1503, 1462, 1399, 1255, 1211, 1171, 1100, 1044, 930, 850, 775, 701 cm^{-1}, MS (m/z): HRMS (ESI) Calcd. for $C_{59}H_{69}O_{13}$ ($[M+H]^+$): 985.4738, found: 985.4743.

3c: White solid, 79%, m.p. 110-112 °C; ^{1}H NMR (400 MHz, DMSO-d_6) δ: 12.61 (s, 1H, COOH), 7.87 (d, *J* = 8.8 Hz, 2H, ArH), 6.97 (d, *J* = 8.8 Hz, 2H, ArH), 6.77-6.76 (m, 10H, ArH), 4.00 (t, *J* = 6.4 Hz, 2H, CH_2), 3.85-3.80 (m, 4H, CH_2,), 3.65-3.64 (m, 34H, CH_2, OCH_3),3.82 (t, *J* = 6.4 Hz, 2H, CH_2), 3.65-3.61 (m, 34H, CH_2, OCH_3), 1.78-1.64 (m, 6H, CH_2), 1.53-1.40 (m, 6H, CH_2), 0.90 (t, *J* = 7.2 Hz, 3H, CH_3), ^{13}C NMR (100 MHz, DMSO-d_6) δ: 167.5, 162.5, 150.4, 150.3, 150.3, 150.3, 149.5, 149.5, 131.6, 128.0, 127.9, 127.9, 114.5, 113.8, 113.7, 113.7, 68.1, 68.0, 67.8, 55.8, 31.6, 28.8, 25.8, 25.7, 19.3, 14.1, IR (KBr) υ: 3634, 2940, 2860, 2836, 2661, 1686, 1605, 1503, 1462, 1399, 1304, 1254, 1211, 1171, 1100, 1043, 930, 850, 775, 703 cm^{-1}, MS (m/z):HRMS (ESI) Calcd. for $C_{60}H_{71}O_{13}$ ($[M+H]^+$): 999.4895, found: 999.4903.

3. General procedure for the preparation of diamido-bridged bispillar[5]arenes 5a–d: A mixture of pillar[5]arene oxybutoxybenzoic acid **3a** (0.6 mmol, 0.58g), amido-functionalized pillar[5]arenes **4a–d** (0.6 mmol), HOBt (1.0 mmol, 0.14g) and EDCl (1.0 mmol, 0.19 g) in dry $CHCl_3$ (10.0 mL) was stirred at room temperature for twelve hours. The solvent was removed under reduced pressure. The residue was subjected to column chromatography with methylene dichloride/methanol (v/v = 30:1–10:1) as eluent to give the pure product **5a–d** for analysis.

5a: White solid, 29%, m.p. 126-128 °C; ^{1}H NMR (600 MHz, $CDCl_3$) δ: 7.51 (d, *J* = 7.2 Hz, 2H, ArH), 6.99-6.87 (m, 6H, ArH), 6.83-6.67 (m, 16H, ArH), 6.43 (brs, 1H, NH), 4.32 (s, 2H, CH_2), 4.10 (t, *J* = 6.0 Hz, 2H, CH_2), 4.00 (brs, 1H, NH), 3.93 (t, *J* = 6.0 Hz, 2H, CH_2), 3.86-3.63 (m, 68H, 10CH_2, 16OCH_3), 3.36 (brs, 2H, CH_2), 2.09-2.04 (m, 2H, CH_2), 2.02-1.97 (m, 2H, CH_2),

1.85-1.81 (m, 2H, CH_2), 1.75-1.70 (m, 2H, CH_2), 1.62 (s, 4H, CH_2), 1.58-1.54 (m, 2H, CH_2), 1.51-1.47 (m, 2H, CH_2), 1.28-1.22 (m, 2H, CH_2), 0.99 (t, J = 7.2 Hz, 3H, CH_3), 0.94 (t, J = 7.2 Hz, 3H, CH_3); ^{13}C NMR (100 MHz, $CDCl_3$) δ: 161.2, 151.5, 150.9, 150.8, 150.7, 150.6, 150.5, 150.3, 150.2, 150.1, 149.7, 148.9, 129.0, 128.7, 128.3, 128.2, 128.1, 128.0, 127.8, 127.7, 127.6, 127.0, 115.0, 114.9, 114.8, 114.6, 114.5, 114.2, 114.1, 114.0, 113.9, 113.8, 113.2, 112.9, 112.8, 68.1, 67.9, 67.7, 66.5, 56.1, 55.9, 55.8, 55.7, 55.4, 55.3, 52.4, 38.1, 31.9, 31.8, 29.7, 29.6, 29.4, 26.5, 26.4, 22.6, 19.4, 14.0, 13.9; IR (KBr) υ: 3397, 2933, 2837, 1667, 1610, 1501, 1460, 1397, 1309, 1210, 1101, 1042, 928, 871, 770 cm^{-1}; MS (m/z): HRMS (ESI) Calcd. for $C_{109}H_{127}N_2O_{23}$ ($[M+H]^+$): 1832.8863, found: 1832.8849.

5b: White solid, 44%, m.p. 136-138 °C; ^{1}H NMR (600 MHz, $CDCl_3$) δ: 7.71 (d, J = 8.4 Hz, 2H, ArH), 6.97-6.75 (m, 22H, ArH), 6.51 (brs, 1H, NH), 4.43 (brs, 1H, NH), 4.11 (t, J = 6.0 Hz, 2H, CH_2), 3.98-3.92 (m, 4H, CH_2), 3.89 (brs, 2H, CH_2), 3.84-3.64 (m, 68H, 10CH_2, 16OCH_3), 3.57 (brs, 2H, CH_2), 3.32 (brs, 2H, CH_2), 2.09-2.06 (m, 2H, CH_2), 2.01-1.99 (m, 2H, CH_2), 1.81-1.79 (m, 2H, CH_2), 1.79-1.72 (m, 2H, CH_2), 1.57 (s, 4H, CH_2), 1.55-1.48 (m, 4H, CH_2), 0.98 (t, J = 7.2 Hz, 3H, CH_3), 0.95 (t, J = 7.2 Hz, 3H, CH_3); ^{13}C NMR (100 MHz, $CDCl_3$) δ: 161.0, 150.8, 150.7, 150.6, 150.5, 150.4, 150.3, 150.2, 149.7, 148.4, 128.8, 128.6, 128.3, 128.2, 128.1, 128.0, 114.9, 114.8, 114.1, 114.0, 113.9, 68.1, 67.9, 67.7, 55.8, 55.7, 55.6, 36.0, 35.1, 31.9, 31.8, 29.7, 29.6, 29.5, 29.4, 29.3, 26.4, 26.3, 22.6, 19.4, 14.1, 14.0, 13.9; IR (KBr) υ: 3401, 2931, 2857, 1663, 1611, 1501, 1460, 1396, 1307, 1210, 1042, 928, 873, 770 cm^{-1}; MS (m/z): HRMS (ESI) Calcd. for $C_{110}H_{129}N_2O_{23}$ ($[M+H]^+$): 1846.9020, found: 1846.9072.

5c: White solid, 16%, m.p. 130-132 °C; ^{1}H NMR (600 MHz, $CDCl_3$) δ: 7.85 (d, J = 8.4 Hz, 2H, ArH), 7.02-6.96 (m, 8H, ArH), 6.90 (s, 1H, ArH), 6.84-6.75 (m, 12H, ArH), 6.61 (s, 1H, ArH), 5.25 (brs, 1H, NH), 4.62 (brs, 1H, NH), 4.54 (d, J = 5.4 Hz, 2H, CH_2), 4.14 (t, J = 6.0 Hz, 2H, CH_2), 3.94 (t, J = 6.0 Hz, 2H, CH_2), 3.87-3.63 (m, 68H, 10CH_2, 16OCH_3), 3.53 (s, 2H, CH_2), 2.10-2.08 (m, 2H, CH_2), 2.02-2.00 (m, 2H, CH_2), 1.74-1.72 (m, 2H, CH_2), 1.59 (s, 6H, CH_2), 1.51-1.49(m, 2H, CH_2), 1.47-1.43 (m, 2H, CH_2), 1.24 (m, 2H, CH_2), 0.95-0.91 (m, 6H, CH_3), -1.88- -2.14 (m, 4H, CH_2); ^{13}C NMR (100 MHz, $CDCl_3$) δ: 166.5, 161.2, 150.8, 150.7, 150.6, 150.5, 150.4, 150.2, 150.1, 150.0, 149.8, 149.7, 148.4, 131.9, 129.8, 129.0, 128.7, 128.6, 128.4, 128.3, 128.2, 128.1, 128.0, 127.5, 127.4, 126.6, 117.8, 117.0, 114.8, 114.1, 114.0, 113.9, 113.7, 113.1, 113.0, 112.6, 112.4, 68.1, 67.9, 67.8, 66.0, 57.7, 55.8, 55.7, 55.6, 55.4, 55.3, 39.8, 37.2,

31.8, 31.4, 29.7, 29.6, 29.5, 29.4, 28.7, 28.5, 27.4, 26.5, 26.4, 22.8, 22.6, 19.4, 14.0, 13.9; IR (KBr) υ: 3408, 2937, 1670, 1610, 1501, 1460, 1397, 1306, 1210, 1102, 1042, 928, 873, 771, 703 cm^{-1}; MS (m/z): HRMS (ESI) Calcd. for $C_{111}H_{131}N_2O_{23}$ ($[M+H]^+$): 1860.9176, found: 1860.9220.

5d: White solid, 28%, m.p. 129-131 °C; ^1H NMR (600 MHz, $CDCl_3$) δ: 7.85 (d, *J* = 8.4 Hz, 2H, ArH), 7.01-6.74 (m, 22H, ArH), 6.13 (brs, 1H, NH), 5.28 (brs, 1H, NH), 4.59 (d, *J* = 5.4 Hz, 2H, CH_2), 4.12 (t, *J* = 6.0 Hz, 2H, CH_2), 3.92 (t, *J* = 6.0 Hz, 2H, CH_2), 3.83-3.73 (m, 48H, $16OCH_3$), 3.66-3.60 (m, 20H, $10CH_2$), 3.52 (s, 2H, CH_2), 2.09-2.04 (m, 2H, CH_2), 1.99-1.97 (m, 2H, CH_2), 1.74-1.70 (m, 2H, CH_2), 1.62-1.58 (m, 8H, CH_2), 1.51-1.45 (m, 4H, CH_2), 1.24-1.23 (m, 6H, CH_2), 0.95-0.89 (m, 6H, CH_3), 0.07 (s, 2H, CH_2), -0.28 (s, 2H, CH_2), -0.72- -0.97 (m, 2H, CH_2), -1.58- -1.66 (m, 2H, CH_2), -2.07 (s, 2H, CH_2); ^{13}C NMR (100 MHz, $CDCl_3$) δ: 167.6, 166.5, 161.4, 150.9, 150.8, 150.7, 150.6, 150.5, 150.4, 150.3, 150.2, 150.1, 149.7, 147.2, 129.6, 129.3, 128.9, 128.6, 128.5, 128.4, 128.3, 128.2, 128.1, 128.0, 127.9, 127.8, 127.5, 127.1, 115.8, 115.2, 115.0, 114.9, 114.8, 114.3, 114.1, 114.0, 113.9, 112.8, 112.7, 112.2, 68.9, 68.1, 67.9, 67.8, 67.7, 65.9, 57.0, 56.3, 55.9, 55.8, 55.7, 55.6, 55.5, 55.4, 55.3, 55.1, 40.0, 37.9, 31.9, 31.8, 30.1, 29.7, 29.6, 29.5, 29.4, 28.9, 28.8, 28.6, 28.5, 26.4, 26.3, 26.2, 25.9, 24.2, 24.1, 23.4, 22.6, 19.4, 14.1, 13.9; IR (KBr) υ: 3411, 2934, 2858, 1668, 1609, 1501, 1460, 1397, 1306, 1210, 1100, 1043, 929, 872, 770, 704 cm^{-1}; MS (m/z): HRMS (ESI) Calcd. for $C_{113}H_{135}N_2O_{23}$ ($[M+H]^+$): 1888.9489, found: 1888.9523.

4. General procedure for the synthesis of pillar[5]arene di-oxyalkoxybenzoic acid 8: A mixture of di-(ω-bromobutyl)pillar[5]arene **6** (1.0 mmol, 0.990 g), methyl 4-hydroxybenzoate (2.4 mmol, 0.365 g), potassium iodide (2.4 mmol, 0.40 g) and potassium carbonate (40.0 mmol, 5.50 g) in dry acetonitrile (50.0 mL) was refluxed for one day. After cooling, the solid was removed by filtration and the solvent was removed by evaporation at reduced pressure. The residue was subjected to column chromatography with light petroleum and ethyl acetate (v/v = 3:1) to give the product. It was recrystallized from ethanol to give pure product **7**. Then, the product **7** was poured into potassium hydroxide (10.0 mmol, 0.56 g) in 95% ethanol and was refluxed overnight. After cooling, the solution was neutralized with dilute hydrochloric acid to give white precipitate, which was washed with water to give the pure product **8**.

7: White solid, 40%, m.p. 84.5-86.3 °C; ^1H NMR (400 MHz, $CDCl_3$) δ: 7.87 (d, *J* = 4 Hz, 4H, ArH), 6.87 (d, *J* = 4 Hz, 4H, ArH), 6.77-6.72 (m, 10H, ArH), 4.99 (t, *J* = 6 Hz, 4H, OCH_2),

3.89-3.88 (m, 10H, 4OCH_2, 6OCH_3), 3.77-3.75 (m, 10H, CH_2), 3.64-3.57 (m, 24H, OCH_3), 1.94-1.88 (m, 8H, CH_2); ^{13}C NMR (100 MHz, $CDCl_3$); δ: 166.8, 162.7, 150.7, 150.7, 150.7, 150.6, 149.8, 131.5, 128.3, 128.3, 128.3, 128.2, 128.1, 122.3, 114.9, 114.1, 114.1, 114.0, 114.0, 113.8, 113.8, 67.9, 67.6, 60.4, 55.8, 55.8, 55.7, 52.6, 51.8, 29.6, 29.5, 26.2, 25.9, 21.0, 14.1; IR (KBr) υ: 3435, 2936, 2829, 1715, 1606, 1580, 1501, 1466, 1436, 1399, 1281, 1256, 1212, 1168, 1103, 1046, 1010, 975, 930, 880, 849, 772, 732 cm^{-1}; MS (m/z): HRMS (ESI) Calcd. for $C_{67}H_{74}O_{16}$ ($[M+Na]^+$): 1157.4875, found: 1157.4856.

8: White solid, 85%, m.p. 216.6-218.2 °C; ^{1}H NMR (400 MHz, $CDCl_3$) δ: 12.62(s, 2H, COOH), 7.87 (d, J = 4Hz, 4H, ArH), 6.98 (d, J = 4Hz, 4H, ArH), 6.78-6.73 (m, 10H, ArH), 4.09 (s, 4H, CH_2), 3.92-3.57 (m, 36H, 12CH_2, 24OCH_3), 3.34 (s, 2H, CH_2), 1.93-1.90 (m, 8H, CH_2); ^{13}C NMR (100 MHz, $CDCl_3$) δ: 167.4, 162.6, 150.3, 150.2, 149.5, 131.7, 128.0, 127.9, 127.9, 127.8, 123.2, 114.5, 114.5, 114.4, 113.7, 113.7, 113.6, 67.9, 67.7, 55.7, 29.4, 29.2, 29.1, 26.1, 25.9; IR (KBr) υ: 3436, 2931, 1682, 1605, 1579, 1501, 1466, 1427, 1400, 1255, 1212, 1168, 1105, 1047, 929, 849, 774, 698 cm^{-1}; MS (m/z): HRMS (ESI) Calcd. for $C_{65}H_{70}O_{16}Na$ ($[M+Na]^+$): 1129.4592, found: 1129.4540.

5. General procedure for the preparation of diamido-bridged tris-pillar[5]arenes 9a-9d: To a solution of amido-functionalized pillar[5]arenes **4a-4d** (0.22mmol), pillar[5]arene di-oxybutoxybenzoic acid **8** (0.1 mmol, 0.11g) in dry $CHCl_3$ (20.0 mL) was added HOBt (0.25 mmol, 0.033g) and EDCl (0.25 mmol, 0.048 g). The mixture was stirred at room temperature for 24 hours. The solvent was removed under reduced pressure. The residue was subjected to column chromatography with methylene dichloride/methanol (v/v = 12:1) as eluent to give the pure produc**t 9a–d** for analysis.

9a: White solid, 26%, m.p. 129-131℃; ^{1}H NMR (400 MHz, $CDCl_3$) δ: 7.50 (d, J = 8 Hz, 4H, ArH), 6.96-6.65 (m, 34H, ArH), 6.40 (s, 2H, NH), 5.05 (s, 2H, NH), 4.29 (s, 4H, CH_2), 4.09-3.62 (m, 114H, 42CH_2, 72OCH_3), 3.35 (s, 4H, CH_2), 3.14 (brs, 4H, CH_2), 2.06-1.97 (m, 8H, CH_2), 1.84-1.77 (m, 4H, CH_2), 1.58-1.51 (m, 4H, CH_2), 0.97 (t, J = 7.4Hz, 6H, CH_3); ^{13}C NMR (100 MHz, $CDCl_3$) δ: 165.3, 161.2, 151.5, 150.8, 150.8, 150.8, 150.7, 150.7, 150.6, 150.6, 150.5, 150.3, 150.3, 150.3, 150.2, 150.1, 150.0, 148.8, 130.1, 129.0, 128.7, 128.4, 128.3, 128.3, 128.3, 128.2, 128.1, 128.1, 128.0, 127.8, 127.7, 127.7, 127.6, 127.5, 127.0, 114.9, 114.6, 114.2, 114.2, 114.1, 114.0, 114.0, 113.9, 113.9, 113.7, 113.7, 113.5, 113.4, 113.4, 113.4, 113.3, 113.2, 113.2, 113.2,

113.1, 112.9, 112.8, 68.0, 67.6, 66.4, 56.1, 55.8, 55.8, 55.8, 55.8, 55.7, 55.6, 55.6, 55.5, 55.4, 55.3, 55.3, 55.2, 38.1, 31.9, 29.7, 29.6, 29.1, 27.5, 27.5, 27.5, 26.4, 26.4, 26.3, 19.4, 13.9; IR (KBr) υ: 3397, 2935, 2829, 1662, 1608, 1501, 1463, 1400, 1309, 1250, 1045, 928, 878, 848, 771, 700 cm^{-1}; MS (m/z): HRMS (ESI) Calcd. for $C_{167}H_{190}N_4O_{36}Na$ ($[M+Na]^+$): 2851.3091, found: 2851.3060.

9b: White solid, 22%, m.p. 136-138℃; ^1H NMR (400 MHz, $CDCl_3$) δ: 7.72 (d, *J* = 8 Hz, 4H, ArH), 7.01-6.77 (m, 34H, ArH), 6.53 (s, 2H, NH), 4.95 (s, 2H, NH), 4.44 (s, 4H, CH_2), 4.14-3.58 (m, 118H, 46CH_2, 72OCH_3), 3.35(s, 4H, CH_2), 2.10-2.00 (m, 8H, CH_2), 1.84-1.77 (m, 4H, CH_2), 1.59-1.49 (m, 4H, CH_2), 0.99 (t, *J* = 8 Hz, 6H, CH_3), -1.80 (brs, 2H, CH_2); ^{13}C NMR (100 MHz, $CDCl_3$) δ: 161.0, 150.9, 150.8, 150.8, 150.7, 150.6, 150.6, 150.4, 150.3, 150.0, 148.4, 129.1, 128.8, 128.7, 128.7, 128.7, 128.6, 128.6, 128.5, 128.4, 128.4, 128.3, 128.2, 128.1, 128.1, 114.9, 114.2, 114.0, 114.0, 113.9, 68.0, 67.7, 55.8, 55.8, 55.7, 55.3, 36.0, 35.1, 31.9, 29.7, 29.6, 26.5, 26.4, 19.4, 14.0; IR (KBr) υ: 3340, 2935, 2830, 1664, 1608, 1501, 1400, 1309, 1249, 1211, 1044, 828, 878, 849, 771 cm^{-1}; MS (m/z): HRMS (ESI) Calcd. for $C_{169}H_{194}N_4O_{36}Na$ ($[M+Na]^+$): 2879.3404, found: 2879.3399.

9c: White solid, 19%, m.p. 146-148℃; ^1H NMR (400 MHz, $CDCl_3$) δ: 7.86(d, *J* = 6 Hz, 4H, ArH), 7.03-6.77 (m, 34H, ArH), 6.62 (s, 2H, NH), 5.25(s, 2H, NH), 4.54 (s, 4H, CH_2), 4.15(t, *J* = 6 Hz, 4H, CH_2), 3.95-3.53 (m, 118H, 46CH_2, 72OCH_3), 2.10-2.01 (m, 10H, CH_2), 1.47-1.41 (m, 6H, CH_2), 0.93 (t, *J* = 8 Hz, 6H, CH_3), -2.00 (m, 8H, CH_2); ^{13}C NMR (100 MHz, $CDCl_3$) δ: 166.6, 166.4, 161.2, 151.4, 150.9, 150.8, 150.8, 150.7, 150.6, 150.5, 150.5, 150.2, 150.1, 150.0, 149.8, 148.4, 131.9, 129.9, 129.0, 128.7, 128.6, 128.4, 128.3, 128.2, 128.2, 128.1, 128.0, 128.0, 127.5, 127.4, 126.6, 117.8, 117.0, 114.9, 114.3, 114.1, 114.0, 113.9, 113.7, 113.1, 113.1, 113.1, 113.0, 112.9, 112.6, 112.5, 112.4, 70.7, 68.0, 67.7, 66.0, 57.6, 55.8, 55.8, 55.8, 55.7, 55.4, 55.3, 39.8, 37.2, 31.8, 31.3, 29.7, 29.7, 29.6, 28.7, 28.5, 27.4, 26.4, 26.4, 22.9, 22.7, 19.3, 13.9; IR (KBr) υ: 3399, 2935, 2829, 1664, 1608, 1501, 1463, 1400, 1308, 1249, 1211, 1044, 928, 879, 771, 701 cm^{-1}; MS (m/z): HRMS (ESI) Calcd. for $C_{171}H_{198}N_4O_{36}Na$ ($[M+Na]^+$): 2907.3717, found: 2907.3667.

9d: White solid, 17%, m.p. 140-142℃; ^1H NMR (400 MHz, $CDCl_3$) δ: 7.84 (d, *J* = 4 Hz, 4H, ArH), 7.01-6.74 (m, 34H, ArH), 6.14 (s, 2H, NH), 5.28 (s, 2H, NH), 4.58 (s, 4H, CH_2), 4.11 (t, *J* = 4 Hz, 4H, CH_2), 3.91-3.62 (m, 118H, 46CH_2, 72OCH_3), 2.70 (brs, 2H, CH_2), 2.51 (brs, 2H, CH_2), 2.08-1.95 (m, 8H, CH_2), 1.48-1.42 (m, 4H, CH_2), 0.89 (t, *J* = 4 Hz, 6H, CH_3), -0.29 (brs, 4H, CH_2), -0.74 (brs, 2H, CH_2), -0.97 (brs, 2H, CH_2), -1.62 (brs, 4H, CH_2), -2.08 (brs, 4H, CH_2); ^{13}C NMR

(100 MHz, $CDCl_3$) δ:167.6, 166.4, 161.4, 150.9, 150.9, 150.8, 150.8, 150.7, 150.7, 150.6, 150.6, 150.5, 150.4, 150.3, 150.3, 150.2, 150.1, 149.9, 147.2, 129.6, 129.3, 128.8, 128.5, 128.5, 128.4, 128.4, 128.3, 128.2, 128.2, 128.2, 128.1, 128.0, 127.9, 127.8, 127.5, 127.1, 115.8, 115.1, 115.1, 115.0, 114.8, 114.3, 114.3, 114.1, 114.1, 114.0, 113.9, 113.9, 112.8, 112.7, 112.7, 112.2, 112.2, 112.1, 109.9, 68.8, 67.9, 67.7, 65.8, 57.0, 56.2, 55.9, 55.8, 55.8, 55.7, 55.5, 55.5, 55.4, 55.3, 55.0, 53.1, 40.0, 37.9, 31.8, 30.1, 29.7, 29.6, 29.6, 29.5, 29.5, 29.3, 28.8, 28.6, 28.5, 26.4, 26.3, 26.2, 26.1, 24.1, 23.4, 23.3, 19.4, 13.9; IR (KBr) υ: 3409, 2935, 2856, 1673, 1608, 1500, 1464, 1400, 1307, 1249, 1210, 1100, 1045, 928, 879, 850, 771, 702 cm^{-1}; MS (m/z): HRMS (ESI) Calcd. for $C_{175}H_{206}N_4O_{36}Na$ ($[M+Na]^+$): 2963.4343, found: 2963.4339.

Figures of single crystal structures S1-S3

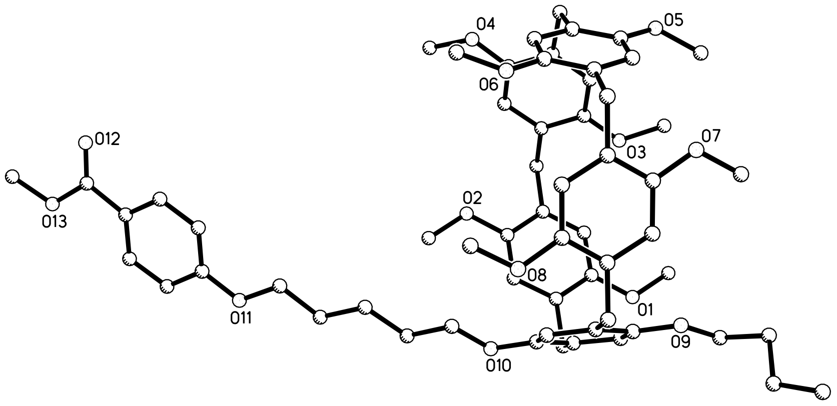

Figure S1: Single crystal structure of pillar[5]arene **2c**.

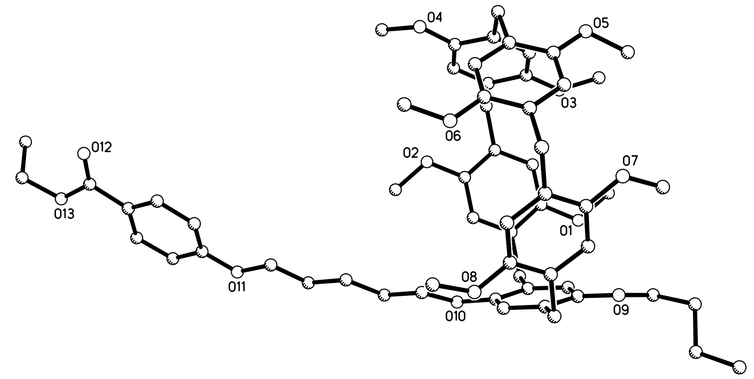

Figure S2: Single crystal structure of pillar[5]arene **2d**.

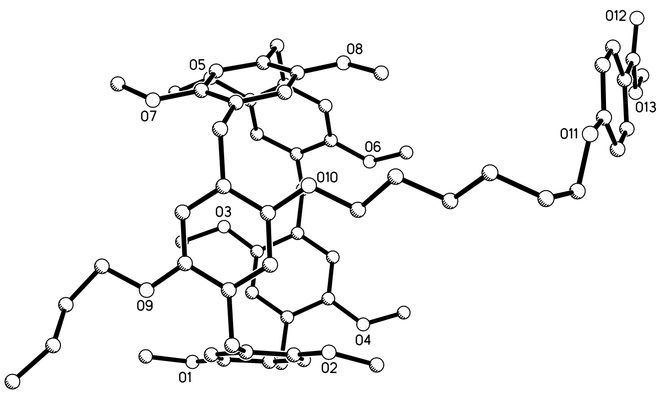

Figure S3: Single crystal structure of pillar[5]arene **2e**.

2D NOESY spectra

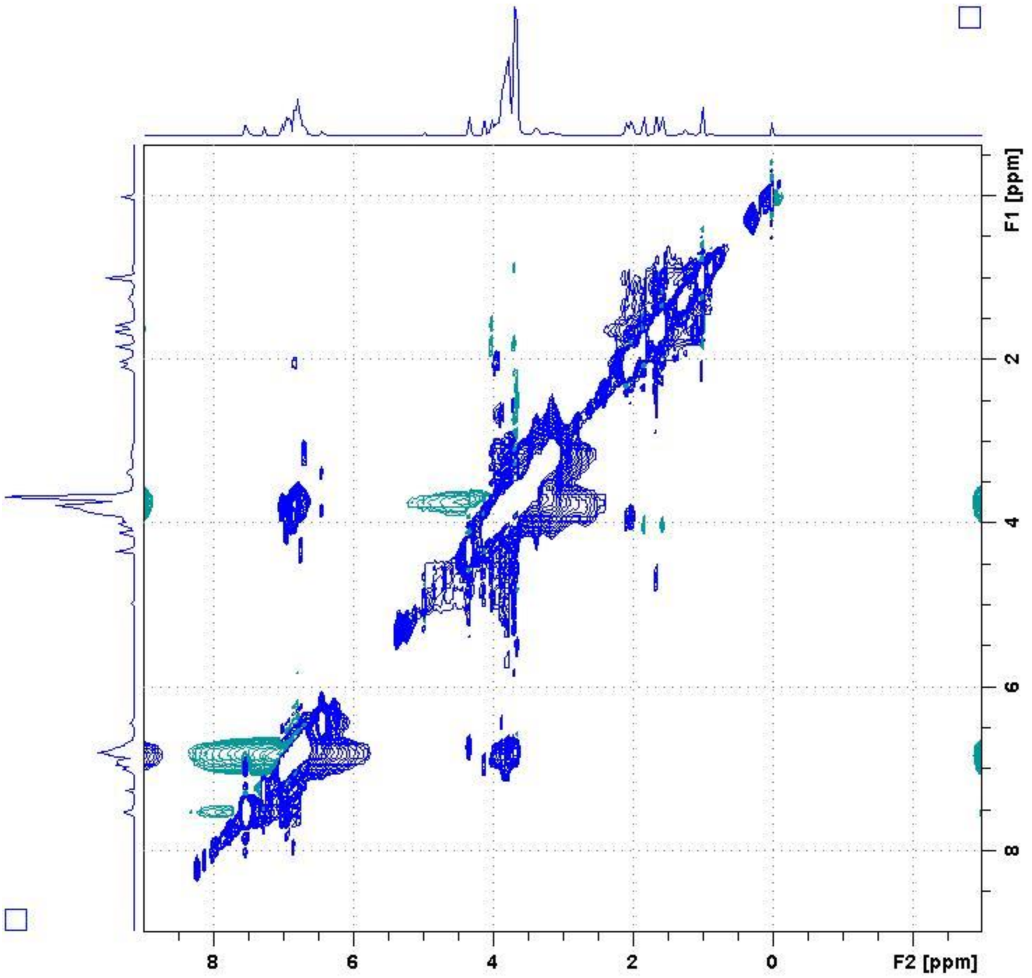

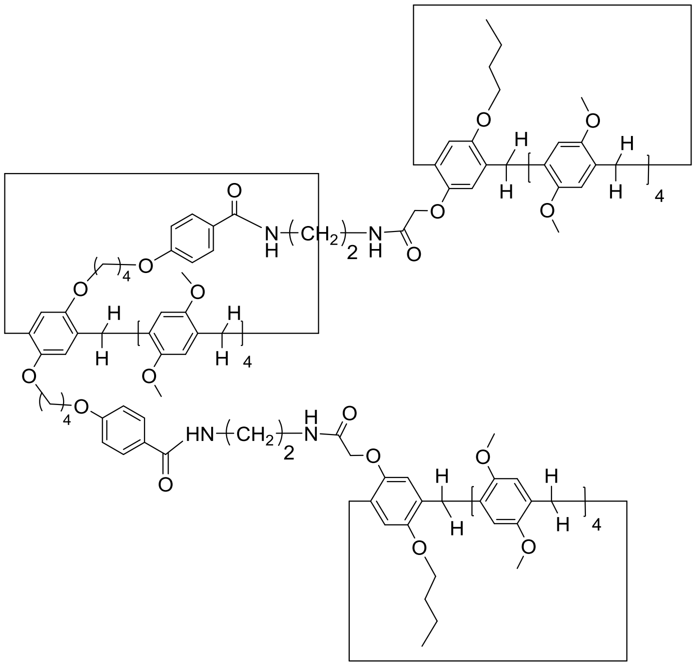

Figure S4: 2D NOESY sepctra of the tris-pillar[5]arene **9a**.

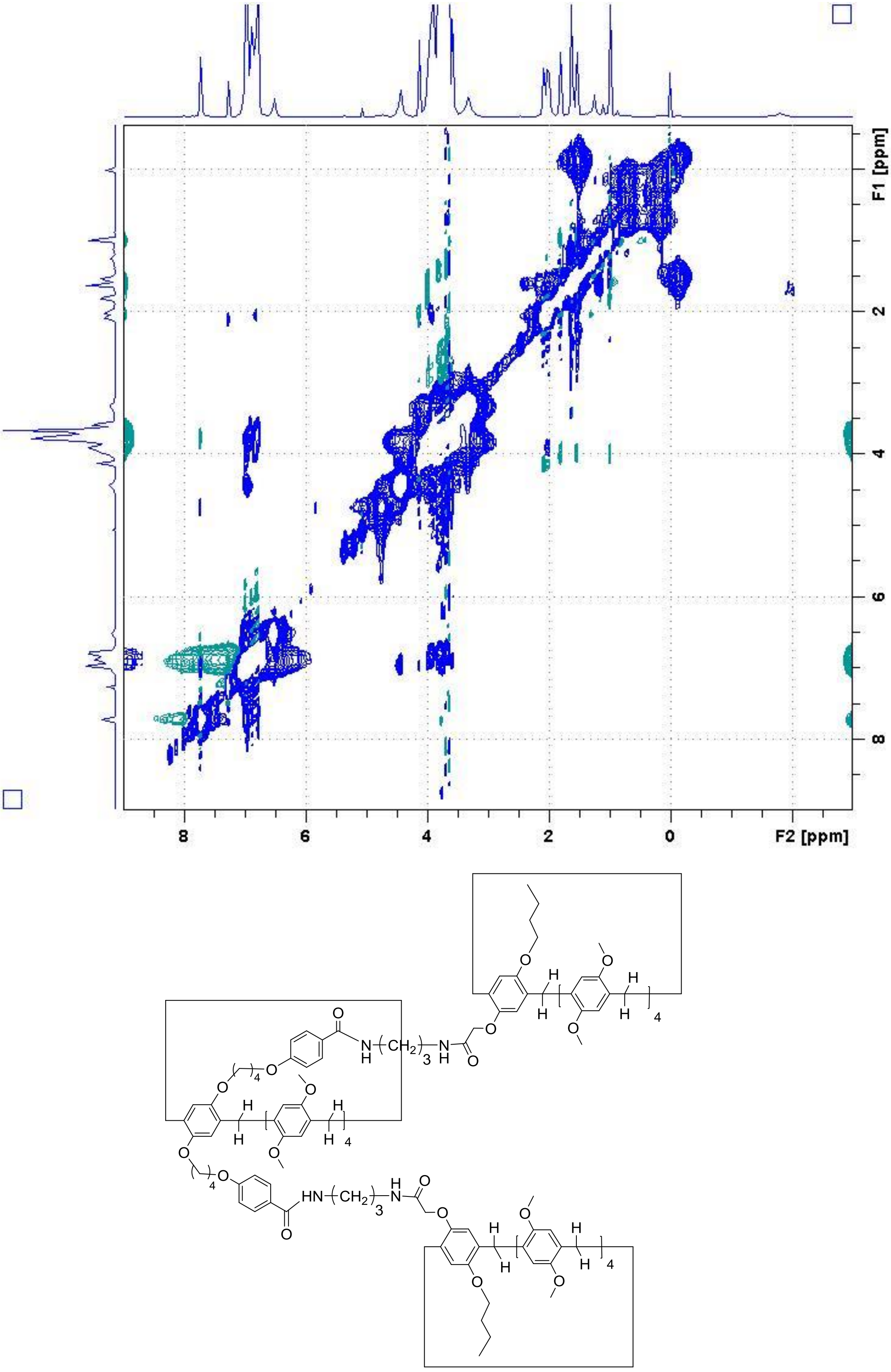

Figure S5: 2D NOESY sepctra of the tris-pillar[5]arene **9b**.

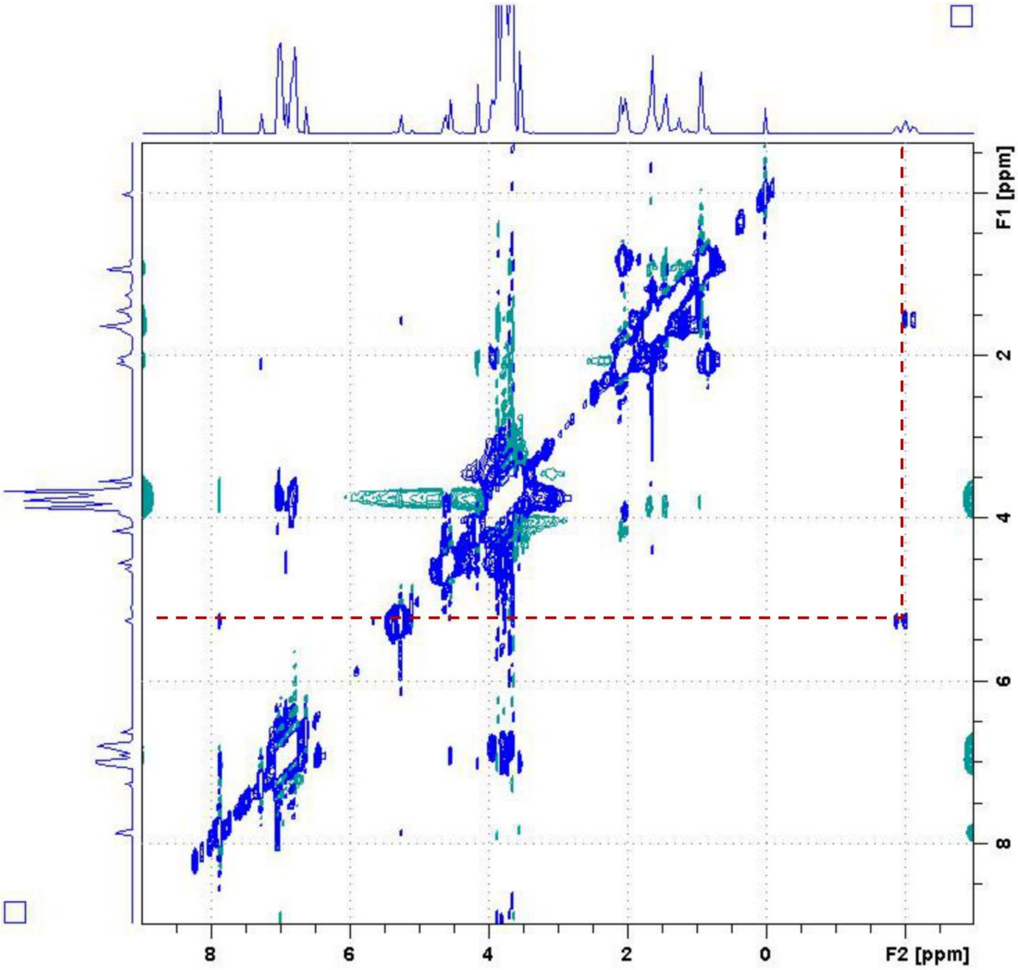

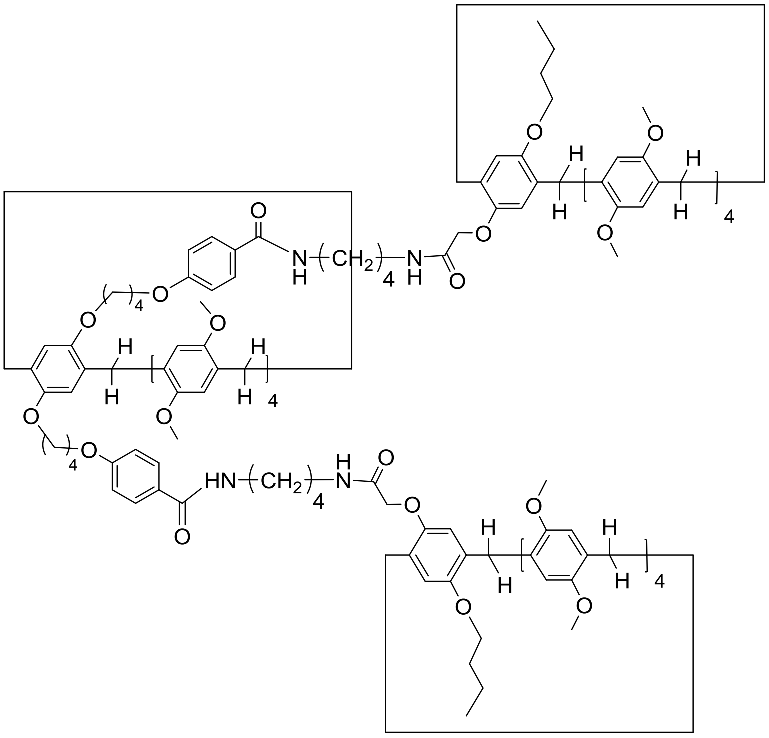

Figure S6: 2D NOESY sepctra of the tris-pillar[5]arene **9c**.

Characterization data and ¹H NMR, ¹³C NMR, HRMS spectra

2a: White solid, 80%, m.p. 88-90 °C; ^{1}H NMR (400 MHz, CDCl$_3$) δ: 7.98 (d, *J* = 4.4 Hz, 2H, ArH), 6.88 (d, *J* = 4.8 Hz, 2H, ArH), 6.80-6.77 (m, 10H, ArH), 4.01-4.00 (m, 2H, CH$_2$), 3.92-3.91 (m, 2H, CH$_2$), 3.89 (s, 3H, OCH$_3$), 3.84 (t, *J* = 6.4 Hz, 2H, CH$_2$), 3.77-3.76 (m, 10H, CH$_2$), 3.68-3.64 (m, 24H, OCH$_3$), 1.96 (brs, 4H, CH$_2$), 1.78-1.72 (m, 2H, CH$_2$), 1.54-1.47 (m, 2H, CH$_2$), 0.95 (t, *J* = 7.2 Hz, 3H, CH$_3$), ^{13}C NMR (100 MHz, CDCl$_3$) δ: 166.8, 162.7, 150.7, 150.7, 150.7, 150.7, 150.6, 150.1, 149.7, 131.5, 128.3, 128.3, 128.1, 122.4, 114.9, 114.1, 114.0, 114.0, 114.0, 113.9, 113.9, 68.1, 67.8, 67.6, 60.5, 55.8, 55.7, 55.7, 55.7, 55.7, 55.6, 51.8, 31.8, 29.7, 29.6, 29.5, 26.2, 26.0, 19.4, 13.9, IR (KBr) υ: 3579, 3414, 3052, 2944, 2862, 2835, 2750, 1928, 1713, 1660, 1610, 1503, 1460, 1400, 1260, 1211, 1177, 1105, 1045, 983, 931, 849, 772, 770 cm^{-1}, MS (m/z): HRMS (ESI) Calcd. for $C_{59}H_{69}O_{13}$ ([M+H]$^+$): 985.4738, found: 985.4749.

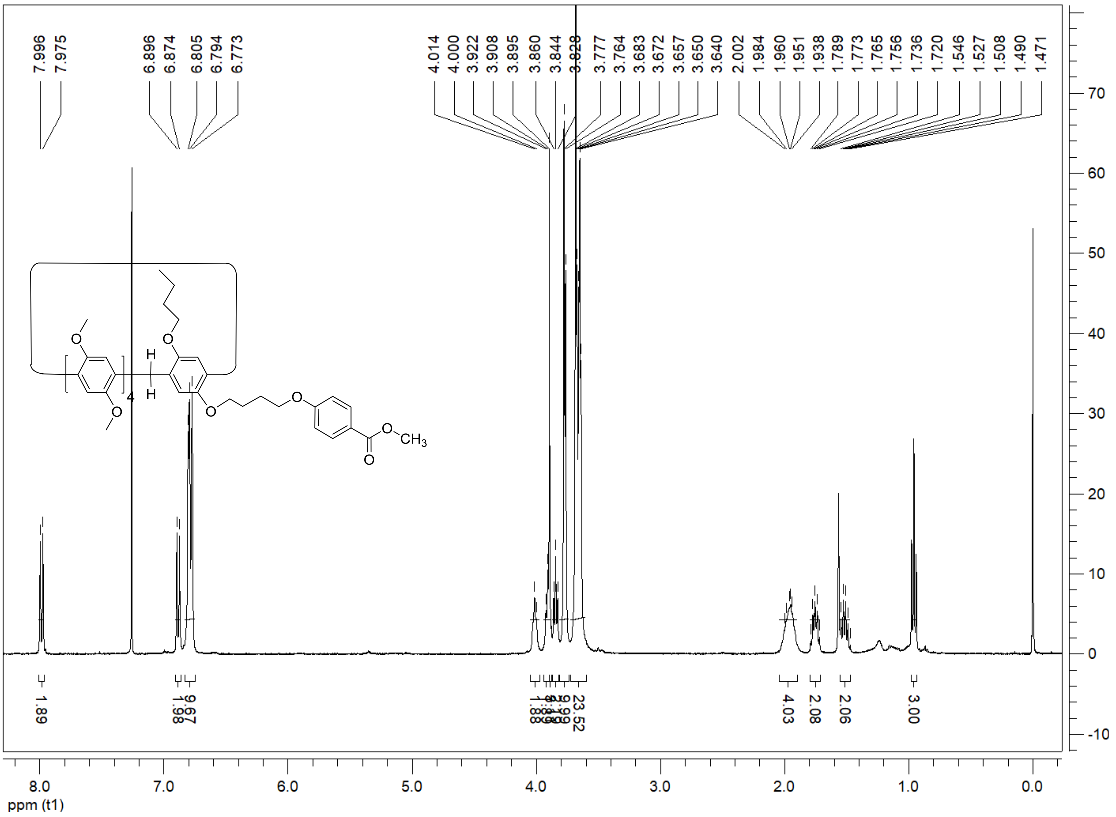

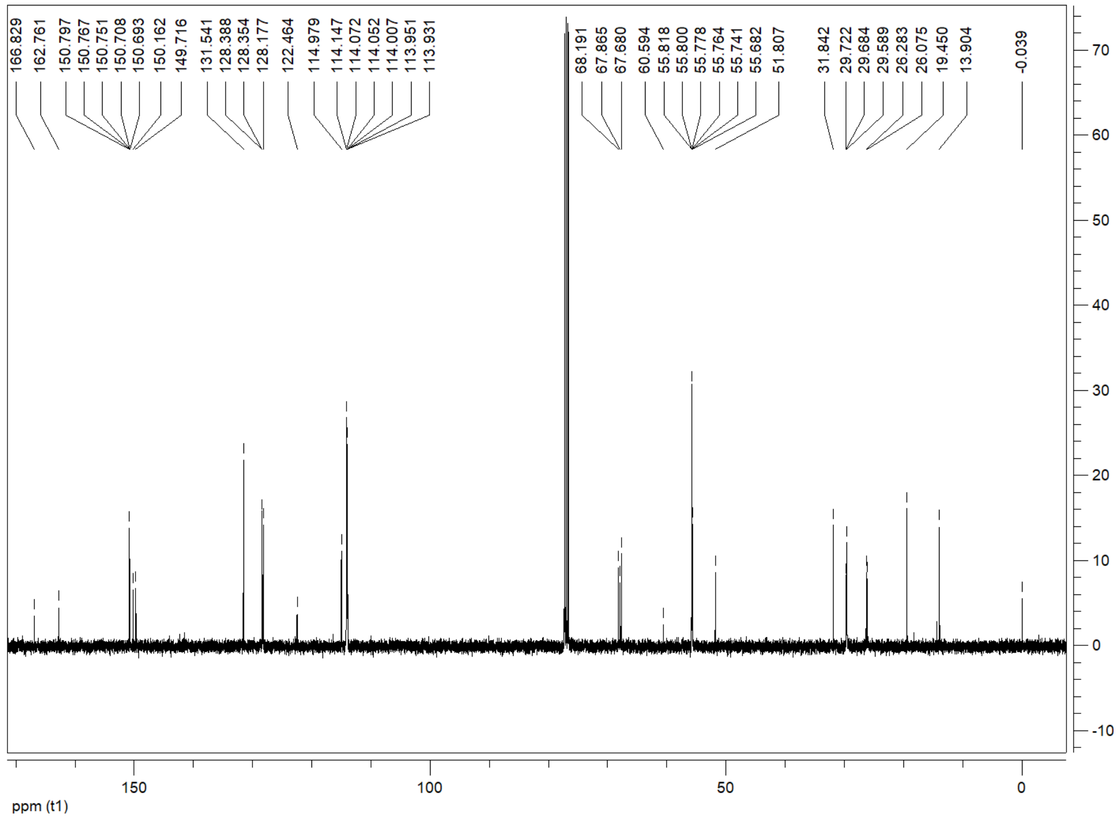
166.829
162.761
150.797
150.767
150.751
150.708
150.693
150.162
149.716
131.541
128.388
128.354
128.177
122.464
114.979
114.147
114.072
114.052
114.007
113.951
113.931
68.191
67.865
67.680
60.594
55.818
55.800
55.778
55.764
55.741
55.682
51.807
31.842
29.722
29.684
29.589
26.283
26.075
19.450
13.904
-0.039
ppm (t1)

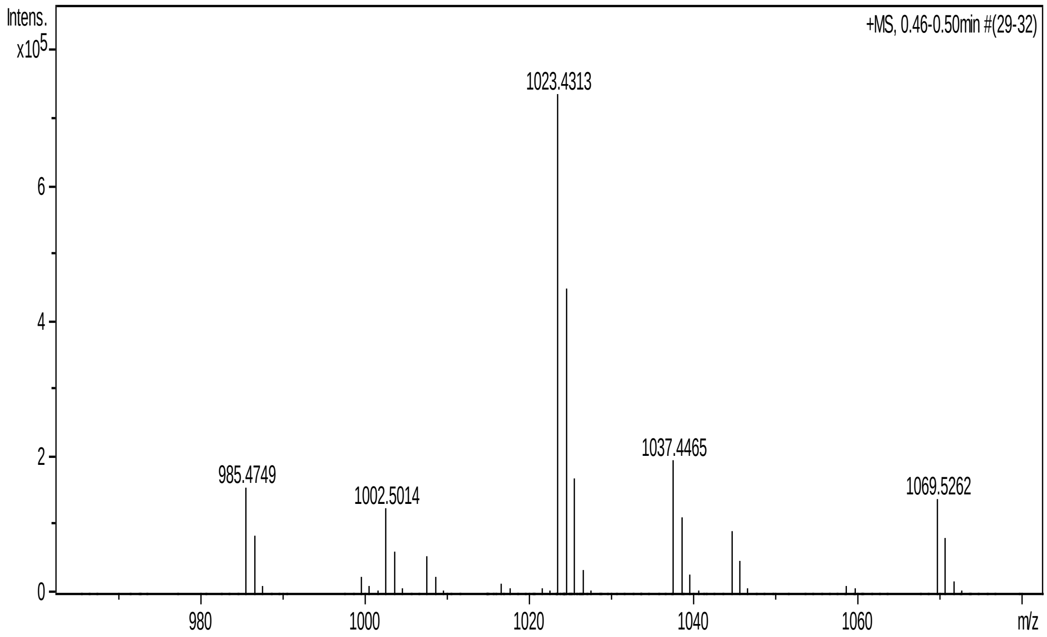
Intens.
x10^5
+MS, 0.46-0.50min #(29-32)
1023.4313
1037.4465
985.4749
1002.5014
1069.5262
m/z

2b: White solid, 78%, m.p. 90-92 °C; ^{1}H NMR (400 MHz,$CDCl_3$) δ: 7.98 (d, *J* = 8.8 Hz, 2H, ArH), 6.88 (d, *J* = 8.8 Hz, 2H, ArH), 6.78-6.75 (m, 10H, ArH), 4.37-4.32 (m, 2H, CH_2), 4.01-4.00 (m, 2H, CH_2), 3.89 (t, *J* = 5.2 Hz, 2H, CH_2), 3.83 (t, *J* = 6.4 Hz, 2H, CH_2), 3.77-3.76 (m, 10H, CH_2), 3.65-3.60 (m, 24H, OCH_3), 1.94 (brs, 4H, CH_2), 1.77-1.70 (m, 2H, CH_2), 1.52-1.47 (m, 2H, CH_2), 1.38 (t, *J* = 7.2 Hz, 3H, CH_3), 0.94 (t, *J* = 7.6 Hz, 3H, CH_3), ^{13}C NMR (100 MHz, $CDCl_3$) δ: 166.4, 162.7, 150.7, 150.6, 150.6, 150.6, 150.6, 150.1, 149.6, 131.5, 128.4, 128.4, 128.3, 128.3, 128.2, 122.7, 114.8, 114.0, 113.9, 113.8, 113.8, 68.1, 67.9, 67.6, 60.6, 55.8, 55.8, 55.7, 55.7, 55.6, 31.8, 29.6, 29.6, 29.5, 29.5, 26.3, 19.4, 14.3, 13.9, IR (KBr) υ: 3462, 3051, 2944, 2863, 2835, 2750, 1934, 1708, 1607, 1503, 1461, 1400, 1361, 1211, 1177, 1104, 1044, 931, 850, 772, 701 cm^{-1}, MS (m/z): HRMS (ESI) Calcd. for $C_{60}H_{71}O_{13}$ ($[M+H]^+$): 999.4895, found: 999.4910.

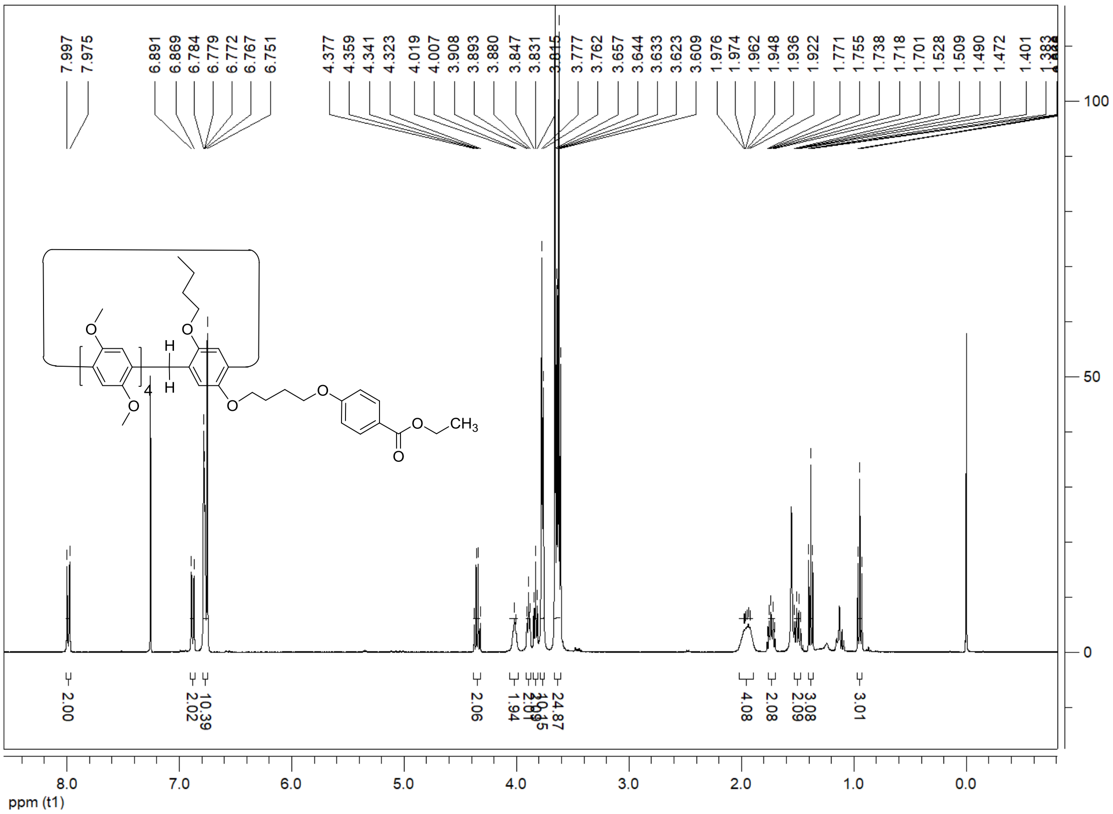

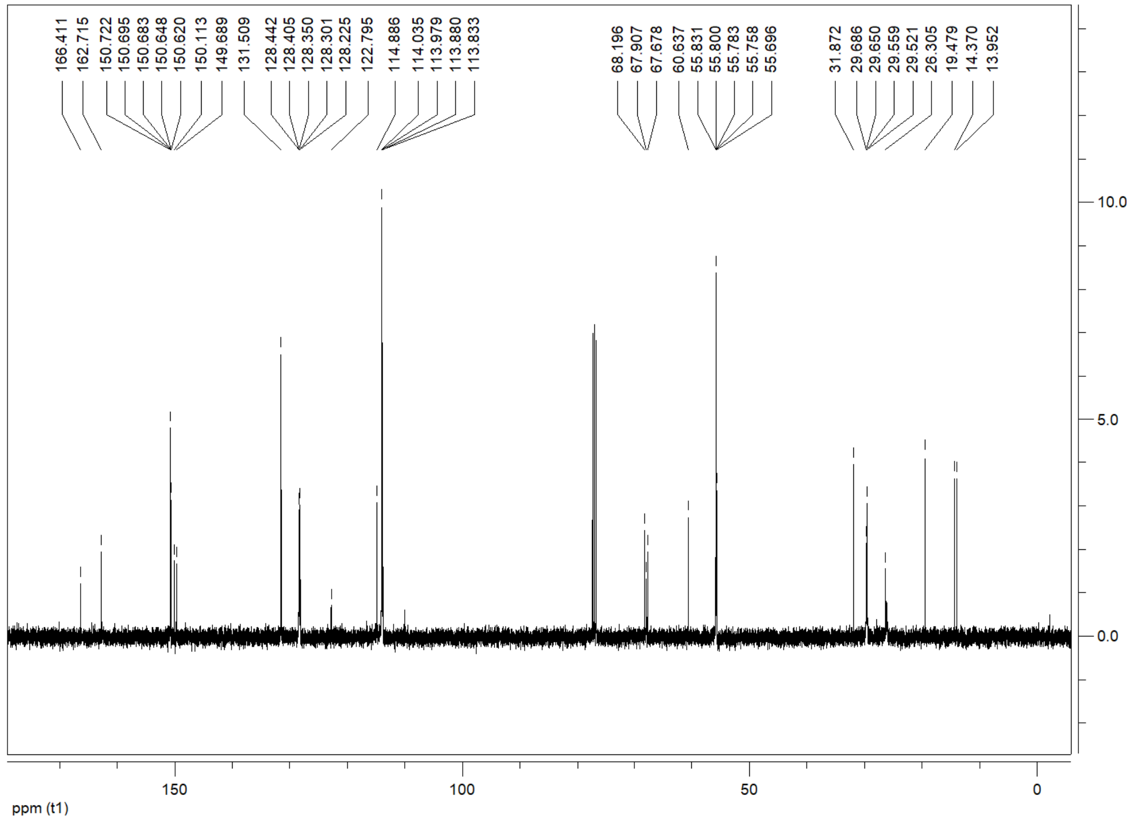
166.411
162.715
150.722
150.695
150.683
150.648
150.620
150.113
149.689
131.509
128.442
128.405
128.350
128.301
128.225
122.795
114.886
114.035
113.979
113.880
113.833
68.196
67.907
67.678
60.637
55.831
55.800
55.783
55.758
55.696
31.872
29.686
29.650
29.559
29.521
26.305
19.479
14.370
13.952
10.0
5.0
0.0
150
100
50
0
ppm (t1)

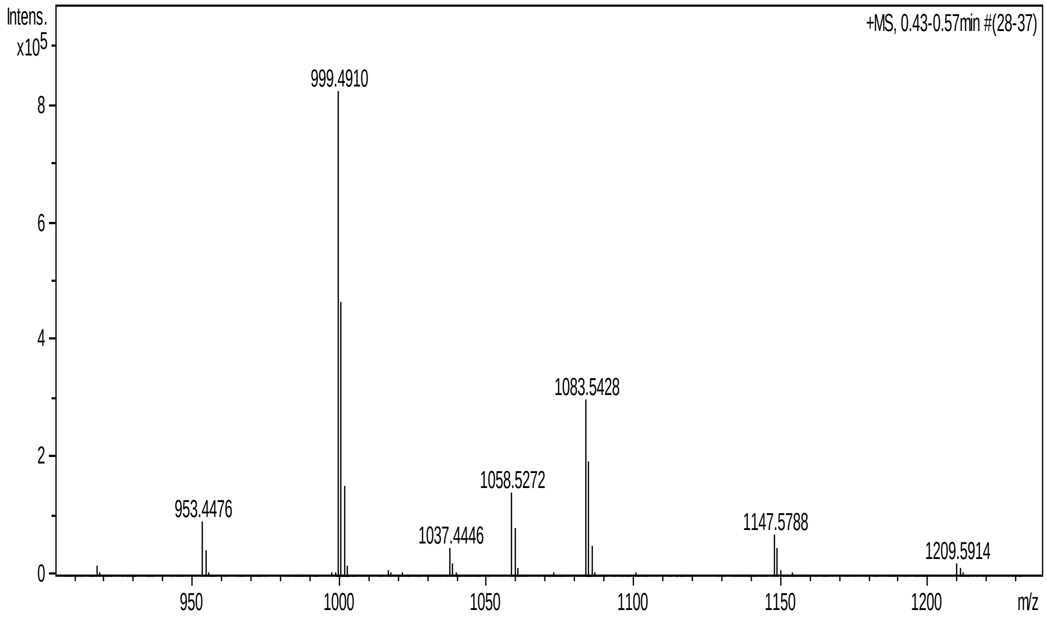
Intens.
x10^5
+MS, 0.43-0.57min #(28-37)
999.4910
1083.5428
1058.5272
953.4476
1037.4446
1147.5788
1209.5914
m/z

2c: White solid, 81%, m.p. 99-101 °C; ^{1}H NMR (400 MHz, DMSO-d_6) δ: 7.90 (d, J = 8.4 Hz, 2H, ArH), 7.01 (d, J = 8.4 Hz, 2H, ArH), 6.77-6.75 (m, 10H, ArH), 4.04 (brs, 2H, CH_2), 3.86-3.81 (m, 7H, CH_2,OCH_3), 3.65-3.61 (m, 34H, CH_2, OCH_3), 1.77 (brs, 4H, CH_2), 1.69-1.59 (m, 4H, CH_2), 1.48-1.42 (m, 2H, CH_2), 0.90 (t, J = 7.6 Hz, 3H, CH_3), ^{13}C NMR (100 MHz, $CDCl_3$) δ: 166.8, 162.8, 150.7, 150.6, 150.6, 150.6, 150.0, 149.7, 131.4, 128.3, 128.1, 115.0, 114.8, 114.0, 68.1, 55.7, 51.8, 31.8, 29.6, 29.5, 29.4, 29.4, 19.4, 13.9, IR (KBr) υ: 3845, 3579, 3408, 3072, 2943, 2864, 2835, 2742, 1927, 1714, 1606, 1504, 1462, 1399, 1257, 1211, 1107, 1045, 930, 870, 850, 773, 701 cm^{-1}, MS (m/z): HRMS (ESI) Calcd. for $C_{60}H_{71}O_{13}$ ($[M+H]^+$): 999.4895, found: 999.4907.

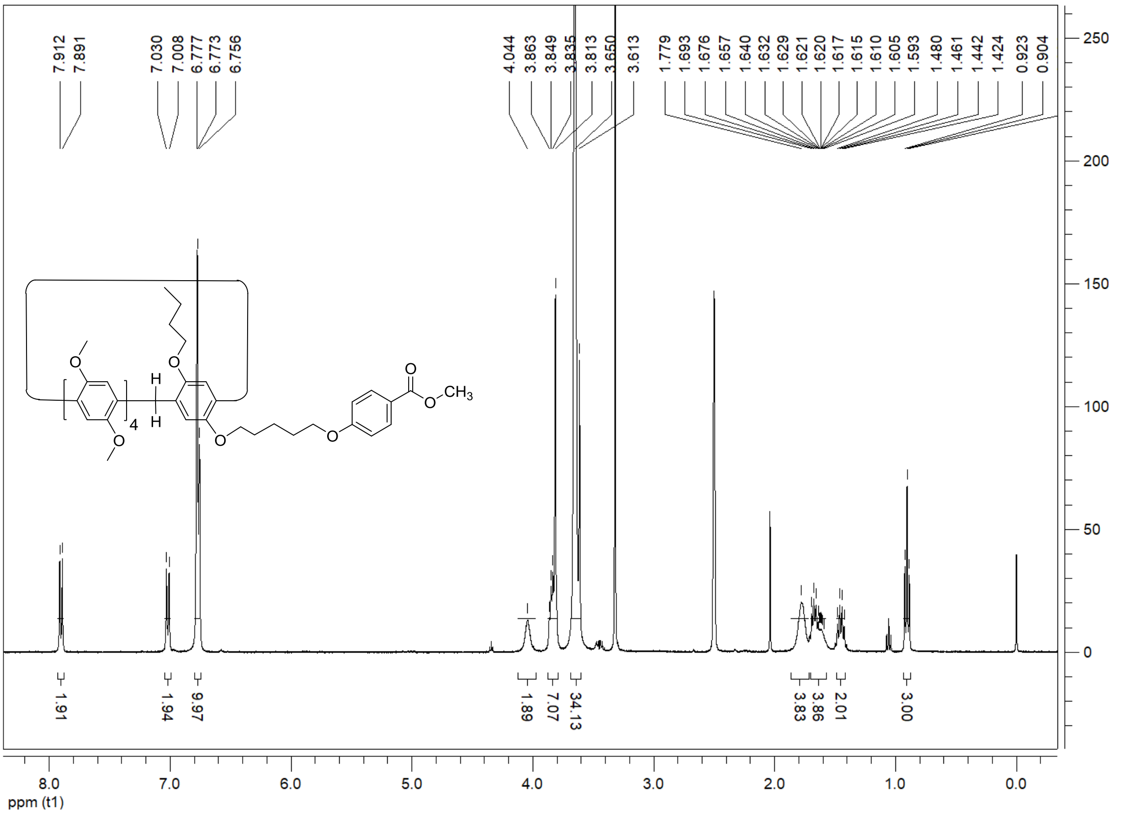

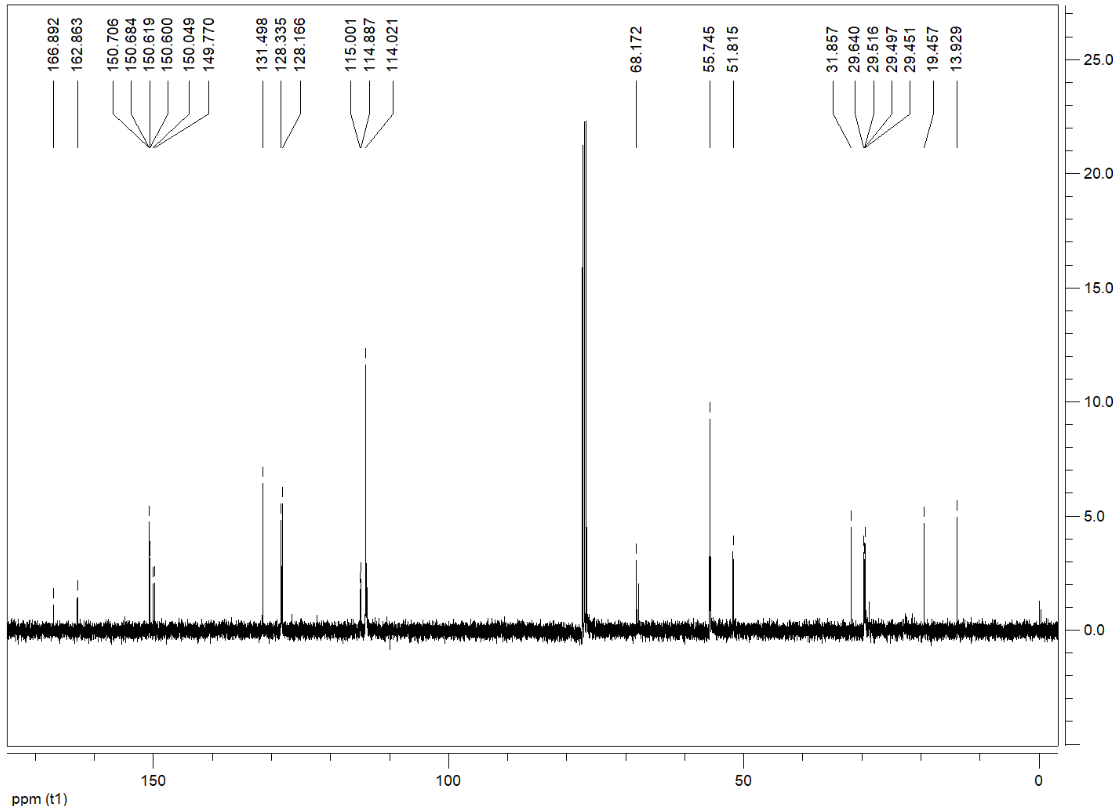
166.892
162.863
150.706
150.684
150.619
150.600
150.049
149.770
131.498
128.335
128.166
115.001
114.887
114.021
68.172
55.745
51.815
31.857
29.640
29.516
29.497
29.451
19.457
13.929
ppm (t1)

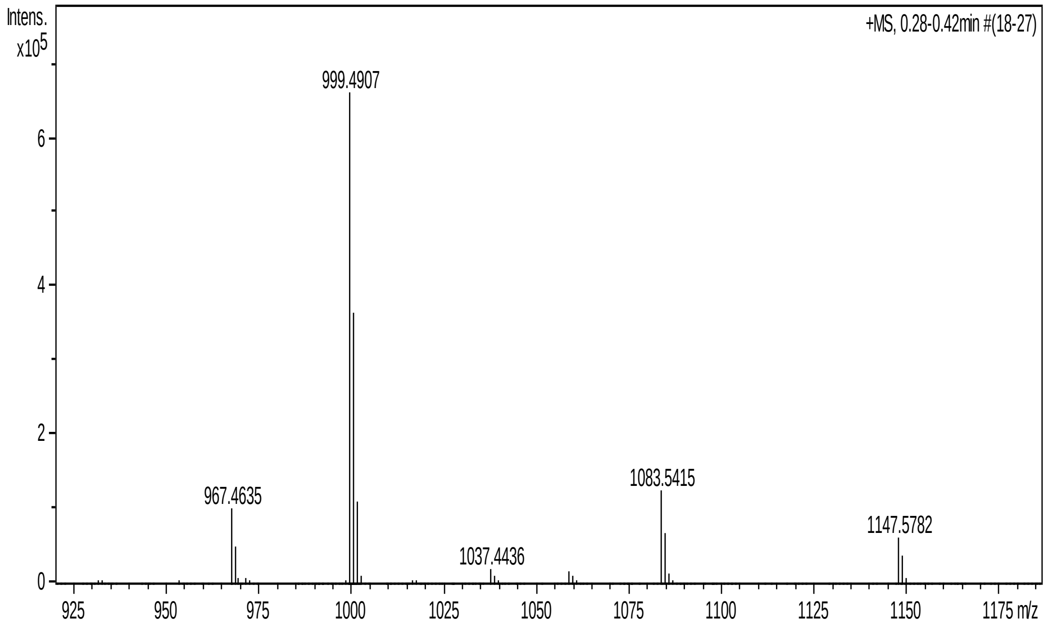
Intens.
x10^5
+MS, 0.28-0.42min #(18-27)
999.4907
967.4635
1037.4436
1083.5415
1147.5782
m/z

2d: White solid, 76%, m.p. 96-98 °C; ^{1}H NMR (400 MHz, DMSO-d_6) δ: 7.90 (d, J = 8.8 Hz, 2H, ArH), 7.01 (d, J = 8.8 Hz, 2H, ArH), 6.77-6.75 (m, 10H, ArH), 4.29-4.24 (m, 2H, CH_2), 4.05 (brs, 2H, CH_2), 3.85-3.80 (m, 4H, CH_2), 3.65-3.61 (m, 34H, CH_2, OCH_3), 1.78 (brs, 4H, CH_2), 1.69-1.64 (m, 4H, CH_2), 1.47-1.42 (m, 2H, CH_2), 1.29 (t, J = 7.2 Hz, 3H, CH_3), 0.90 (t, J = 7.6 Hz, 3H, CH_3), ^{13}C NMR (100 MHz, $CDCl_3$) δ: 166.4, 162.8, 162.8, 150.5, 150.4, 150.4, 149.9, 149.6, 131.4, 128.5, 128.4, 128.3, 116.6, 114.6, 114.0, 113.7, 113.7, 113.6, 113.5, 68.1, 67.9, 60.6, 57.8, 55.7, 55.7, 31.9, 29.5, 19.5, 17.5, 14.3, 14.0, IR (KBr) υ: 3578, 3051, 2942, 2865, 2835, 1708, 1606, 1503, 1462, 1399, 1310, 1259, 1211, 1172, 1105, 1045, 930, 870, 773, 702 cm^{-1}, MS (m/z): HRMS (ESI) Calcd. for $C_{61}H_{73}O_{13}$ ($[M+H]^+$): 1013.5051, found: 1013.5056.

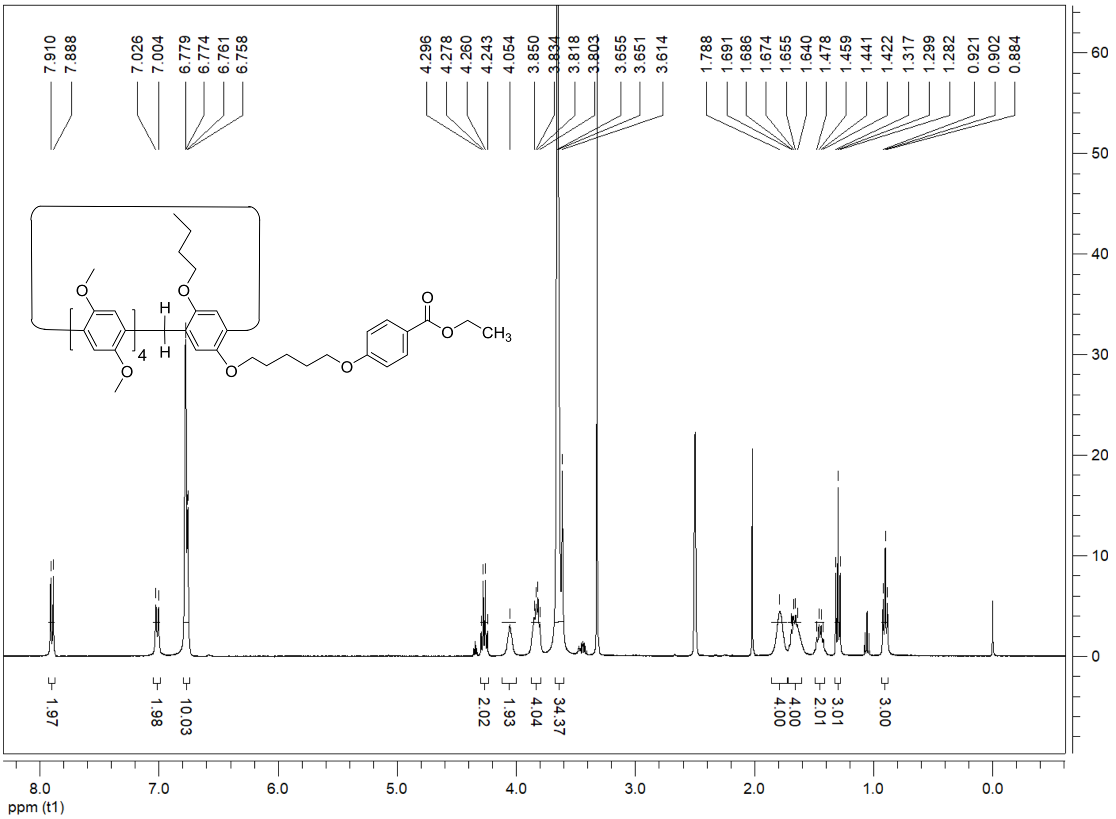

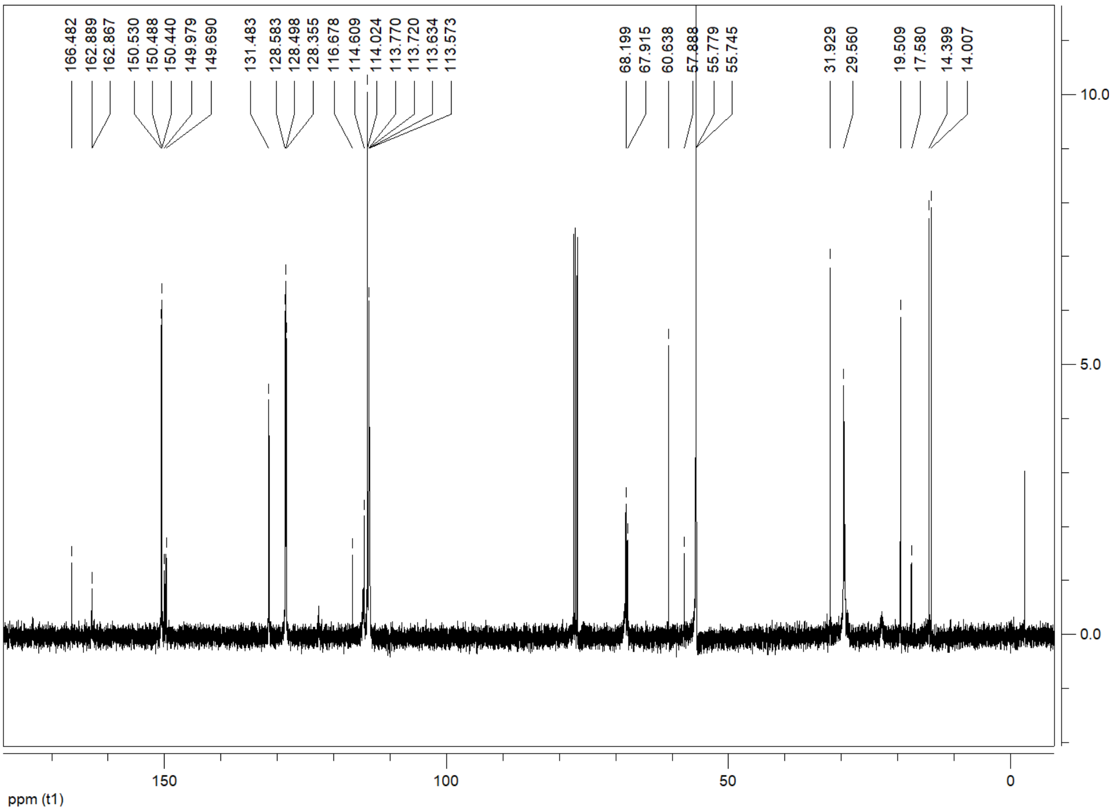
166.482
162.889
162.867
150.530
150.488
150.440
149.979
149.690
131.483
128.583
128.498
128.355
116.678
114.609
114.024
113.770
113.720
113.634
113.573
68.199
67.915
60.638
57.888
55.779
55.745
31.929
29.560
19.509
17.580
14.399
14.007
10.0
5.0
0.0
150
100
50
0
ppm (t1)

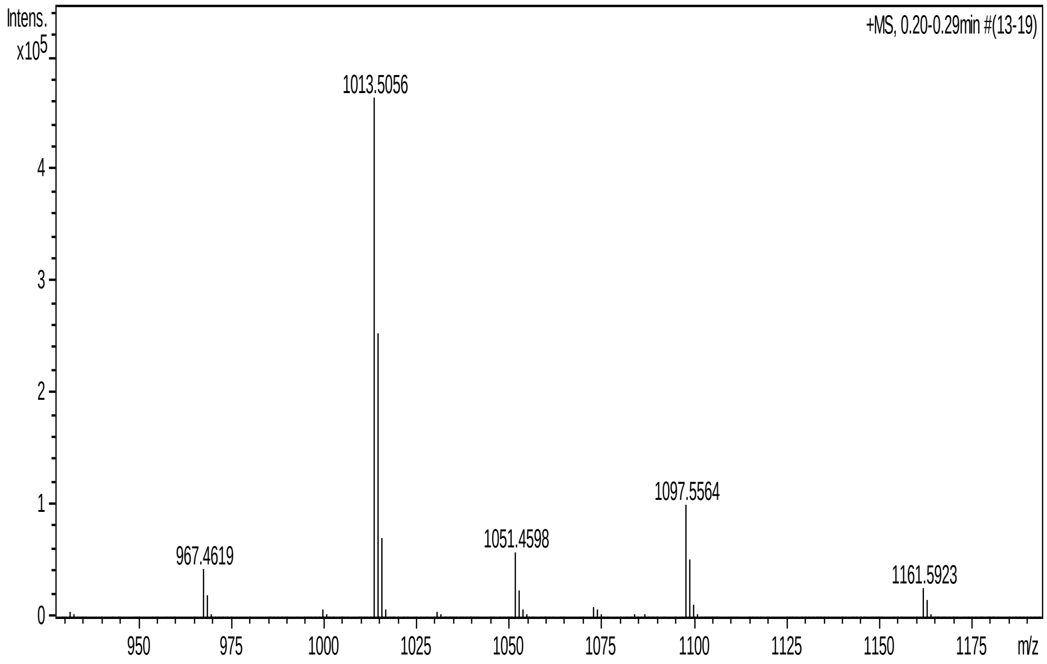
Intens.
x10^5
+MS, 0.20-0.29min #(13-19)
1013.5056
1097.5564
1051.4598
967.4619
1161.5923
4
3
2
1
0
950
975
1000
1025
1050
1075
1100
1125
1150
1175
m/z

2e: White solid, 79%, m.p. 111-113 °C; ^{1}H NMR (400 MHz, $CDCl_3$) δ: 7.98 (d, *J* = 8.4 Hz, 2H, ArH), 6.85 (d, *J* = 8.4 Hz, 2H, ArH), 6.78-6.76 (m, 10H, ArH), 3.89 (s, 3H, OCH_3), 3.82 (brs, 6H, CH_2), 3.76 (brs, 10H, CH_2), 3.64-3.60 (m, 24H, OCH_3), 1.74 (brs, 4H, CH_2), 1.48-1.46 (m, 4H, CH_2), 1.38 (t, *J* = 7.2 Hz, 2H, CH_2), 1.25-1.24 (m, 2H, CH_2), 0.93 (t, *J* = 6.8 Hz, 3H, CH_3), ^{13}C NMR (100 MHz, $CDCl_3$) δ:167.0, 163.1, 150.7, 150.7, 150.6, 150.6, 150.6, 150.1, 131.4, 128.2, 128.2, 128.2, 113.9, 68.1, 60.6, 55.9, 55.8, 55.7, 51.8, 31.8, 29.7, 19.3, 14.4, 13.9, IR (KBr) υ: 3656, 2942, 2862, 2836, 1714, 1606, 1503, 1462, 1399, 1261, 1211, 1174, 1104, 1044, 929, 850, 773, 701 cm^{-1}, MS (m/z): HRMS (ESI) Calcd. for $C_{61}H_{73}O_{13}$ ($[M+H]^+$): 1013.5051, found: 1013.5060.

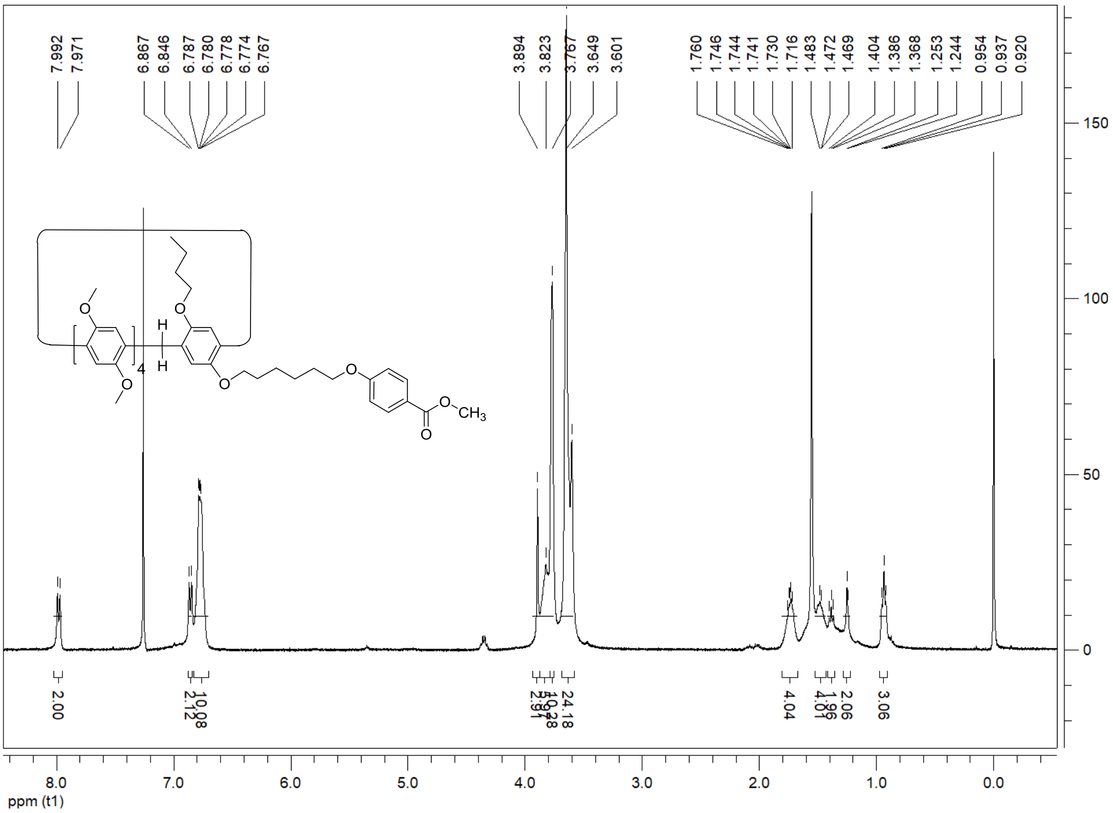

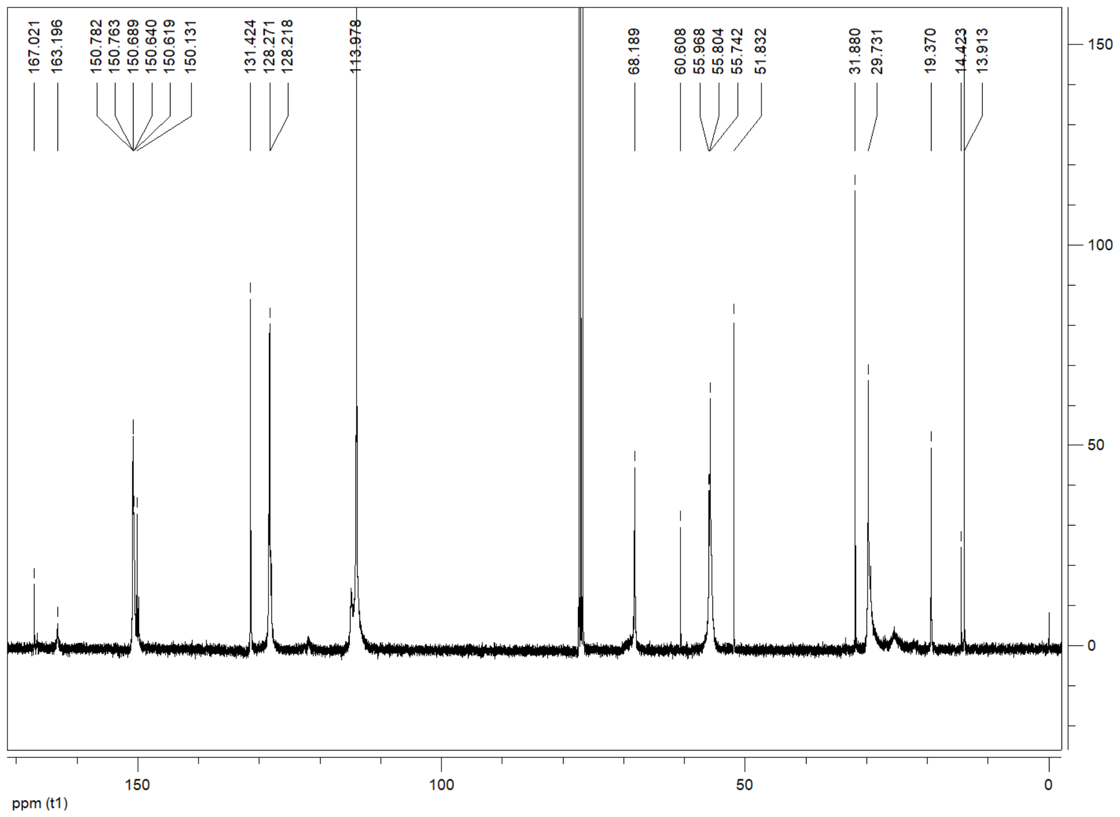
167.021
163.196
150.782
150.763
150.689
150.640
150.619
150.131
131.424
128.271
128.218
113.978
68.189
60.608
55.968
55.804
55.742
51.832
31.880
29.731
19.370
14.423
13.913
ppm (t1)

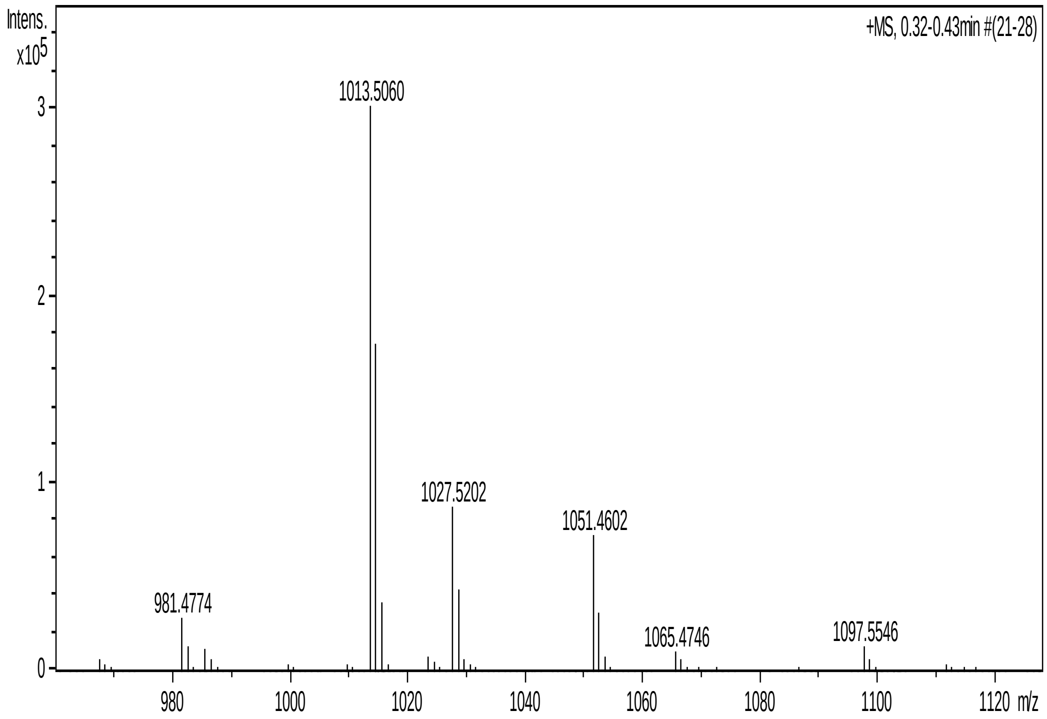
Intens.
x10^5
+MS, 0.32-0.43min #(21-28)
1013.5060
1027.5202
1051.4602
981.4774
1065.4746
1097.5546
m/z

2f: White solid, 78%, m.p. 96-98 °C; ^{1}H NMR (400 MHz, CDCl$_3$) δ: 7.98 (d, *J* = 8.8 Hz, 2H, ArH), 6.86 (d, *J* = 8.8 Hz, 2H, ArH), 6.79-6.73 (m, 10H, ArH), 4.37-4.32 (m, 2H, CH$_2$), 3.85-3.81 (m, 8H, CH$_2$), 3.77-3.76 (m, 10H, CH$_2$), 3.65-3.59 (m, 24H, OCH$_3$), 1.76-1.71 (m, 4H, CH$_2$), 1.62 (brs, 2H, CH$_2$), 1.50-1.47 (m, 4H, CH$_2$), 1.38 (t, *J* = 6.8 Hz, 3H, CH$_3$), 0.94 (t, *J* = 7.2 Hz, 3H, CH$_3$), ^{13}C NMR (100 MHz, CDCl$_3$) δ: 166.5, 163.3, 162.9, 150.7, 150.7, 150.7, 150.7, 150.1, 149.8, 131.3, 128.2, 114.9, 113.9, 68.2, 60.6, 55.8, 55.7, 31.8, 29.7, 19.3, 14.4, 13.9, IR (KBr) υ:3657, 2942, 2862, 2835, 1708, 1607, 1502, 1462, 1398, 1263, 1211, 1173, 1100, 928, 850, 773, 702 cm^{-1}, MS (m/z): HRMS (ESI) Calcd. for $C_{62}H_{75}O_{13}$ ([M+H]$^+$): 1027.5208, found: 1027.5216.

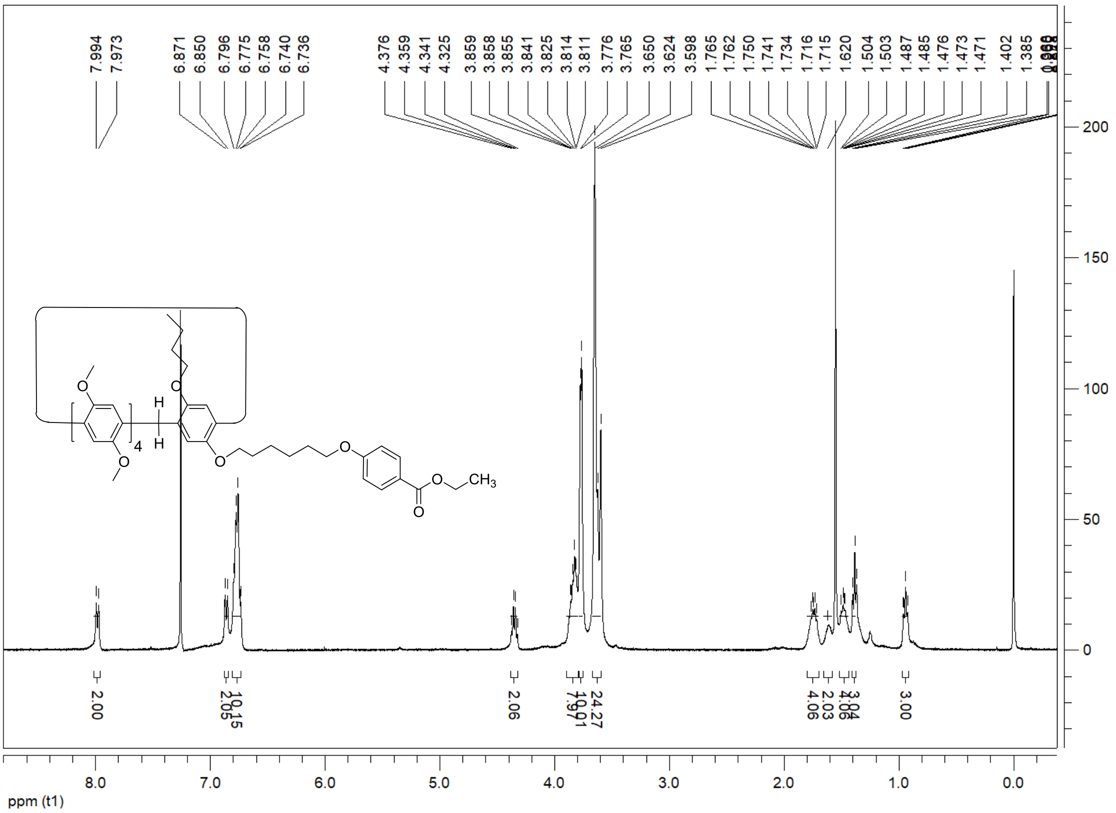

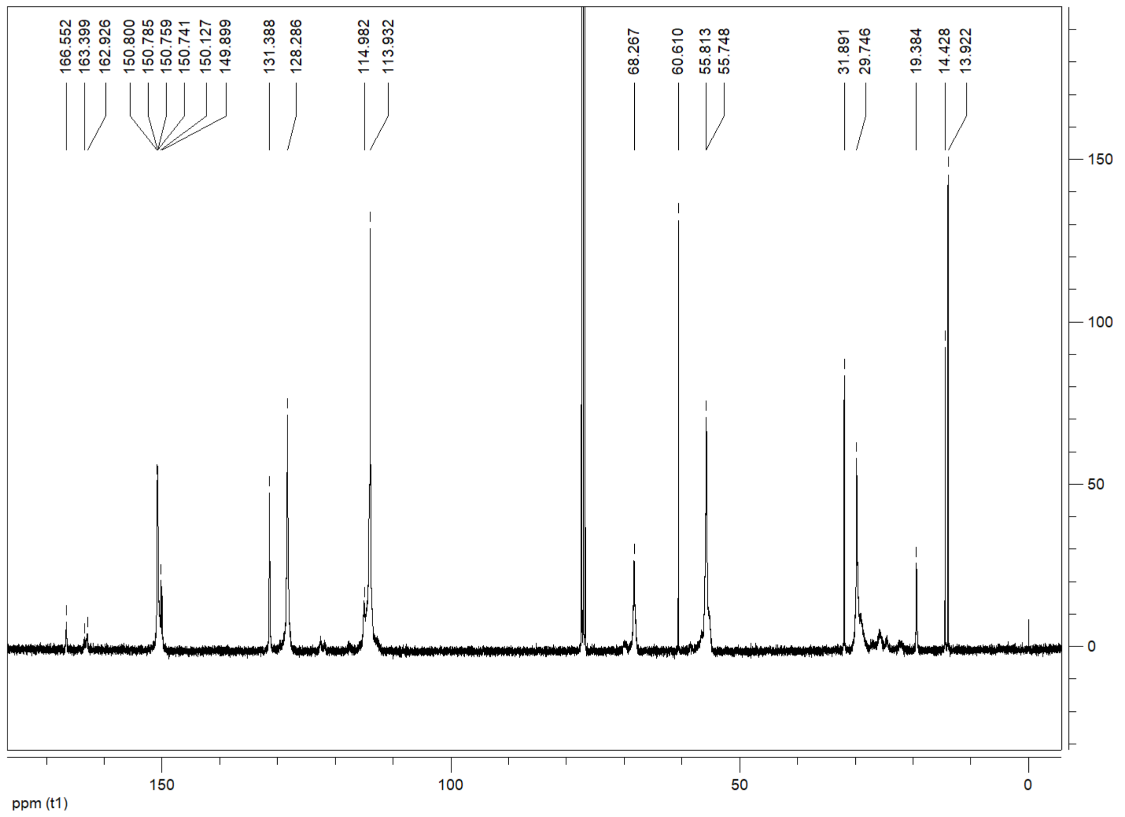
166.552
163.399
162.926
150.800
150.785
150.759
150.741
150.127
149.899
131.388
128.286
114.982
113.932
68.267
60.610
55.813
55.748
31.891
29.746
19.384
14.428
13.922
150
100
50
0
ppm (t1)

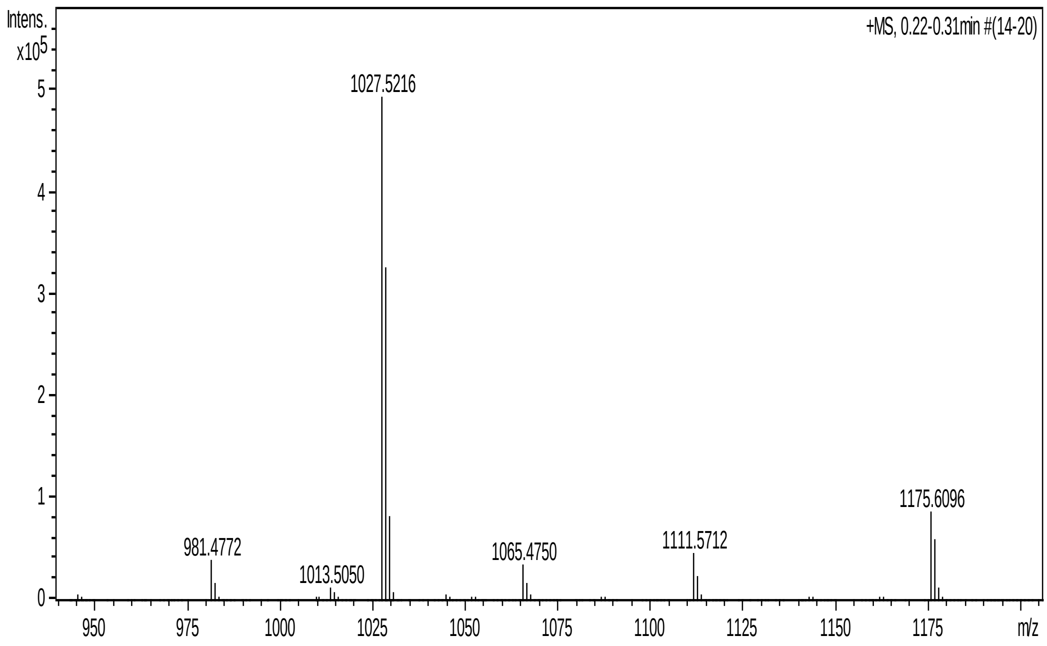
Intens.
x10^5
+MS, 0.22-0.31min #(14-20)
1027.5216
981.4772
1013.5050
1065.4750
1111.5712
1175.6096
m/z

3a: White solid, 81%, m.p. 100-102 °C; ^{1}H NMR (400 MHz, DMSO-d_6) δ: 12.65 (s, 1H, COOH), 7.88 (d, J = 8.8 Hz, 2H, ArH), 7.00 (d, J = 8.8 Hz, 2H, ArH), 6.79-6.75 (m, 10H, ArH), 4.12 (t, J = 6.0 Hz, 2H, CH_2), 3.90 (t, J = 6.0 Hz, 2H, CH_2), 3.82 (t, J = 6.4 Hz, 2H, CH_2), 3.65-3.64 (m, 34H, CH_2, OCH_3), 1.96-1.90 (m, 4H, CH_2), 1.71-1.64 (m, 2H, CH_2), 1.48-1.42 (m, 2H, CH_2), 0.90 (t, J = 7.2 Hz, 3H, CH_3), ^{13}C NMR (100 MHz, DMSO-d_6) δ: 167.6, 162.5, 150.3, 150.3, 150.2, 149.6, 149.4, 131.7, 128.1, 127.9, 127.9, 127.9, 127.9, 127.8, 114.5, 113.7, 113.7, 113.6, 67.9, 55.8, 55.7, 31.6, 29.5, 29.4, 26.2, 25.9, 19.3, 14.1, IR (KBr) υ: 3834, 3577, 3427, 2941, 2862, 2835, 2659, 1926, 1685, 1605, 1504, 1461, 1398, 1309, 1252, 1232, 1187, 1101, 1043, 931, 851, 775, 699 cm^{-1}, MS (m/z): HRMS (ESI) Calcd. for $C_{58}H_{67}O_{13}$ ($[M+H]^+$): 971.4582, found: 971.4587.

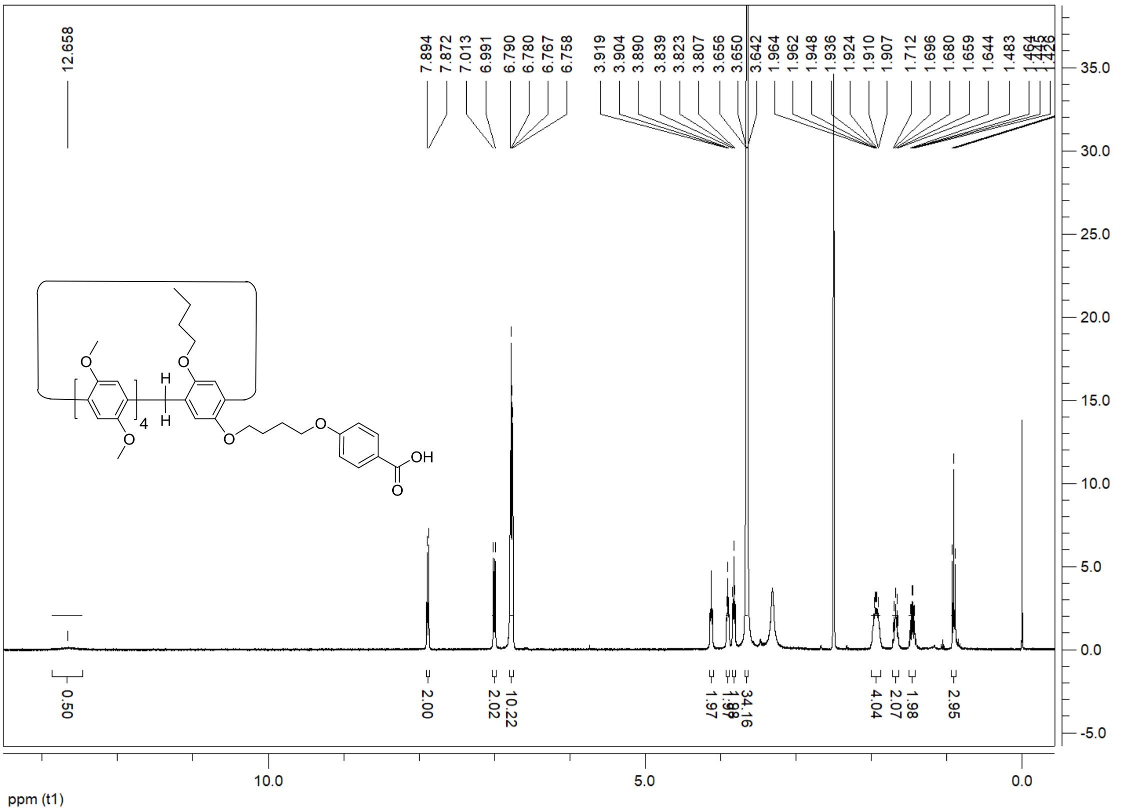

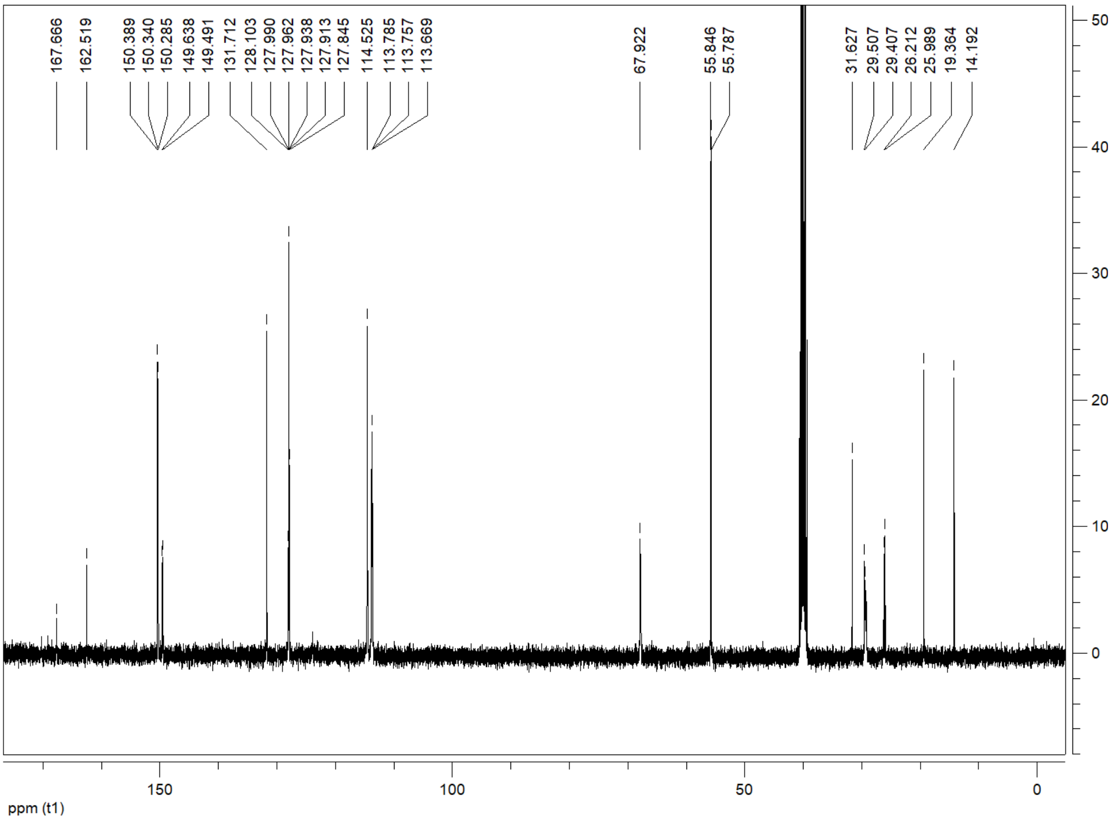
167.666
162.519
150.389
150.340
150.285
149.638
149.491
131.712
128.103
127.990
127.962
127.938
127.913
127.845
114.525
113.785
113.757
113.669
67.922
55.846
55.787
31.627
29.507
29.407
26.212
25.989
19.364
14.192
ppm (t1)

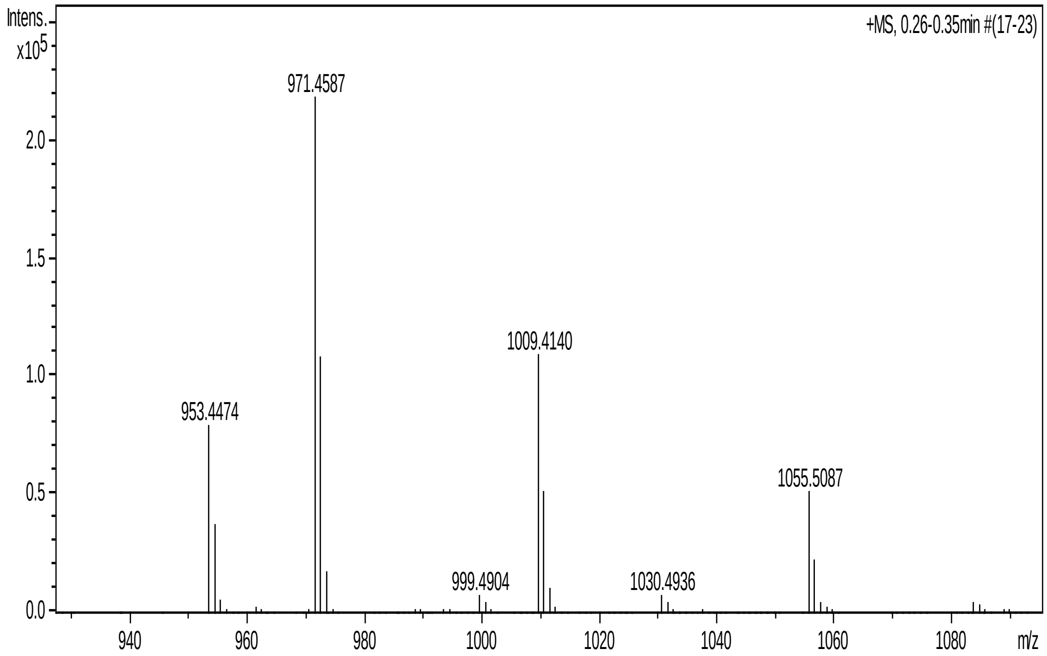
Intens.
x10^5
+MS, 0.26-0.35min #(17-23)
971.4587
1009.4140
953.4474
1055.5087
999.4904
1030.4936
m/z

3b: White solid, 78%, m.p. 106-108 °C; ^{1}H NMR (400 MHz, DMSO-d_6) δ: 12.61 (s, 1H, COOH), 7.88 (d, *J* = 8.8 Hz, 2H, ArH), 7.00 (d, *J* = 8.8 Hz, 2H, ArH), 6.77-6.76 (m, 10H, ArH), 4.05 (t, *J* = 6.0 Hz, 2H, CH_2), 3.87-3.80 (m, 4H, CH_2,), 3.65-3.64 (m, 34H, CH_2, OCH_3), 3.82 (t, *J* = 6.4 Hz, 2H, CH_2), 3.65-3.61 (m, 34H, CH_2, OCH_3), 1.79 (t, *J* = 6.4 Hz, 4H, CH_2), 1.69-1.63 (m, 4H, CH_2), 1.48-1.42 (m, 2H, CH_2), 0.90 (t, *J* = 7.2 Hz, 3H, CH_3), ^{13}C NMR (100 MHz, DMSO-d_6) δ: 167.4, 162.6, 150.3, 150.3, 150.2, 150.2, 149.6, 131.7, 128.0, 128.0, 127.9, 127.8, 127.8, 114.5, 113.7, 113.7, 113.6, 113.6, 68.1, 68.0, 67.8, 55.8, 55.7, 55.7, 31.6, 29.3, 22.7, 19.3, 14.2,δ: IR (KBr) υ: 3656, 2940, 2862, 2835, 1685, 1605, 1503, 1462, 1399, 1255, 1211, 1171, 1100, 1044, 930, 850, 775, 701 cm^{-1}, MS (m/z): HRMS (ESI) Calcd. for $C_{59}H_{69}O_{13}$ ($[M+H]^+$): 985.4738, found: 985.4743.

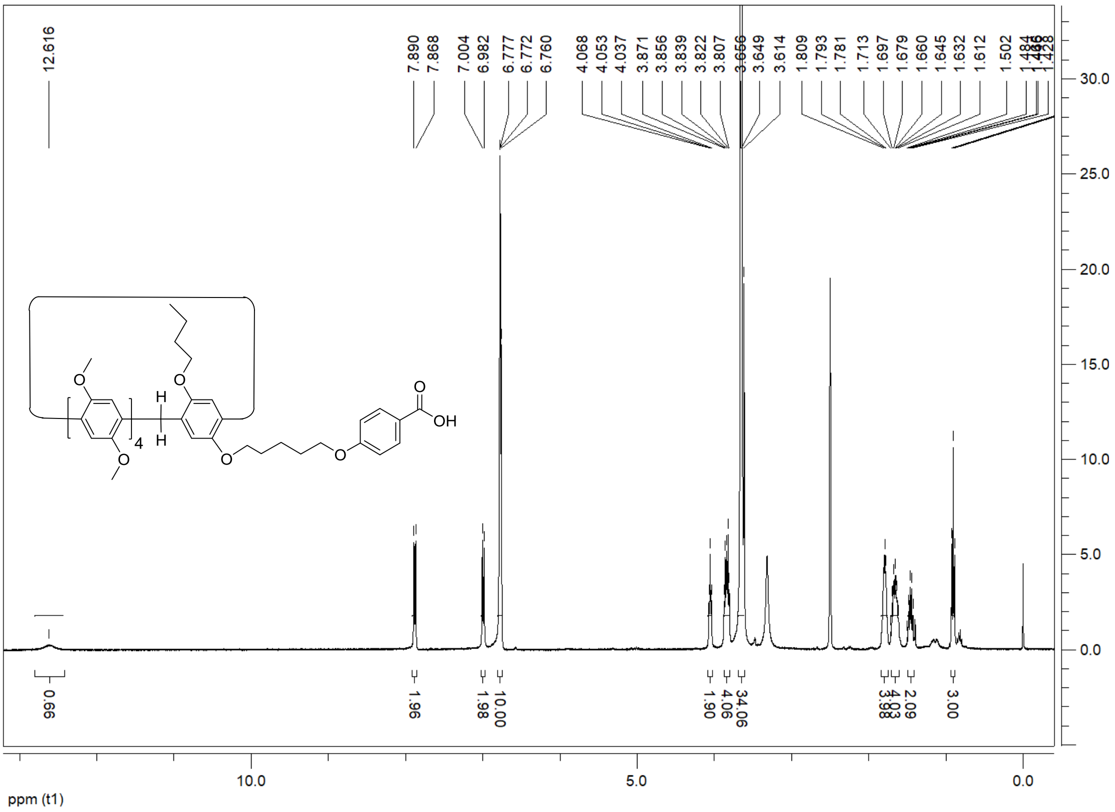

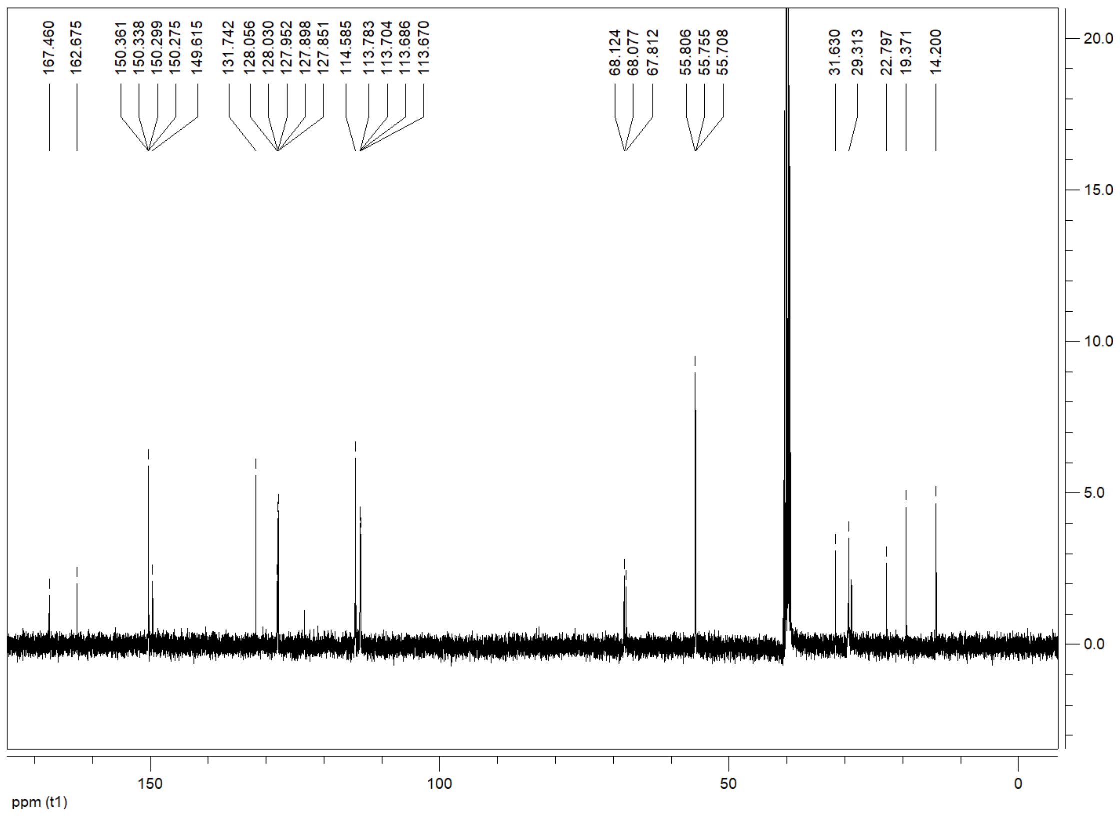
167.460
162.675
150.361
150.338
150.299
150.275
149.615
131.742
128.056
128.030
127.952
127.898
127.851
114.585
113.783
113.704
113.686
113.670
68.124
68.077
67.812
55.806
55.755
55.708
31.630
29.313
22.797
19.371
14.200
ppm (t1)

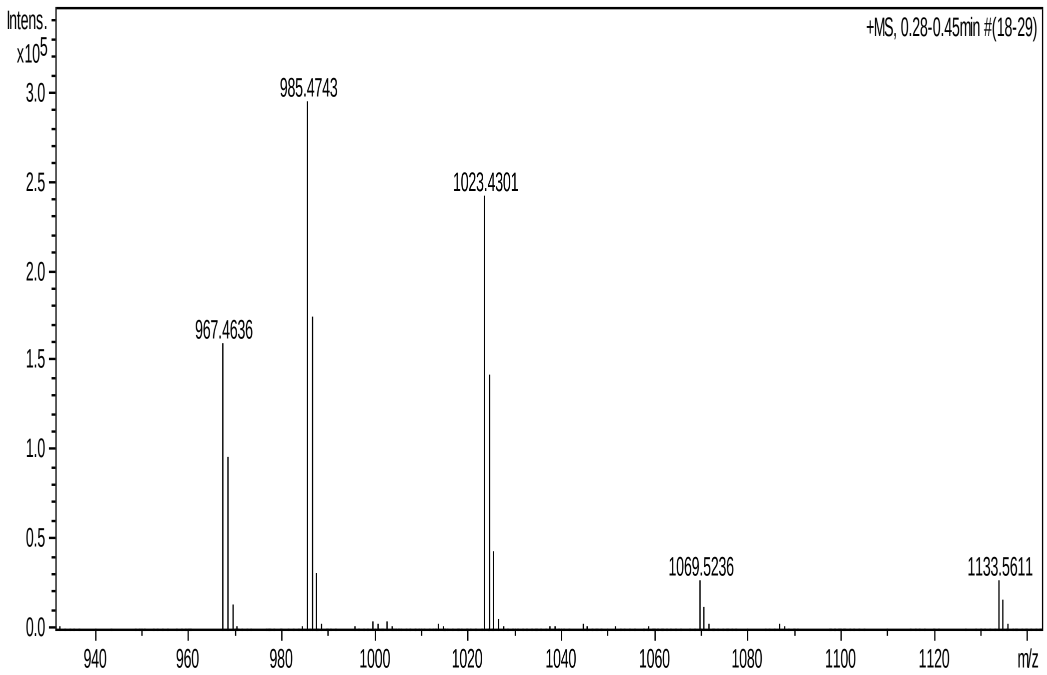
Intens.
x10^5
+MS, 0.28-0.45min #(18-29)
985.4743
1023.4301
967.4636
1069.5236
1133.5611
m/z

3c: White solid, 79%, m.p. 110-112 °C; ^{1}H NMR (400 MHz, DMSO-d_6) δ: 12.61 (s, 1H, COOH), 7.87 (d, J = 8.8 Hz, 2H, ArH), 6.97 (d, J = 8.8 Hz, 2H, ArH), 6.77-6.76 (m, 10H, ArH), 4.00 (t, J = 6.4 Hz, 2H, CH_2), 3.85-3.80 (m, 4H, $CH_{2,}$), 3.65-3.64 (m, 34H, $CH_{2,}$ OCH_3),3.82 (t, J = 6.4 Hz, 2H, CH_2), 3.65-3.61 (m, 34H, $CH_{2,}$ OCH_3), 1.78-1.64 (m, 6H, CH_2), 1.53-1.40 (m, 6H, CH_2), 0.90 (t, J = 7.2 Hz, 3H, CH_3), ^{13}C NMR (100 MHz, DMSO-d_6) δ: 167.5, 162.5, 150.4, 150.3, 150.3, 150.3, 149.5, 149.5, 131.6, 128.0, 127.9, 127.9, 114.5, 113.8, 113.7, 113.7, 68.1, 68.0, 67.8, 55.8, 31.6, 28.8, 25.8, 25.7, 19.3, 14.1, IR (KBr) υ: 3634, 2940, 2860, 2836, 2661, 1686, 1605, 1503, 1462, 1399, 1304, 1254, 1211, 1171, 1100, 1043, 930, 850, 775, 703 cm^{-1}, MS (m/z):HRMS (ESI) Calcd. for $C_{60}H_{71}O_{13}$ ([M+H]$^+$): 999.4895, found: 999.4903.

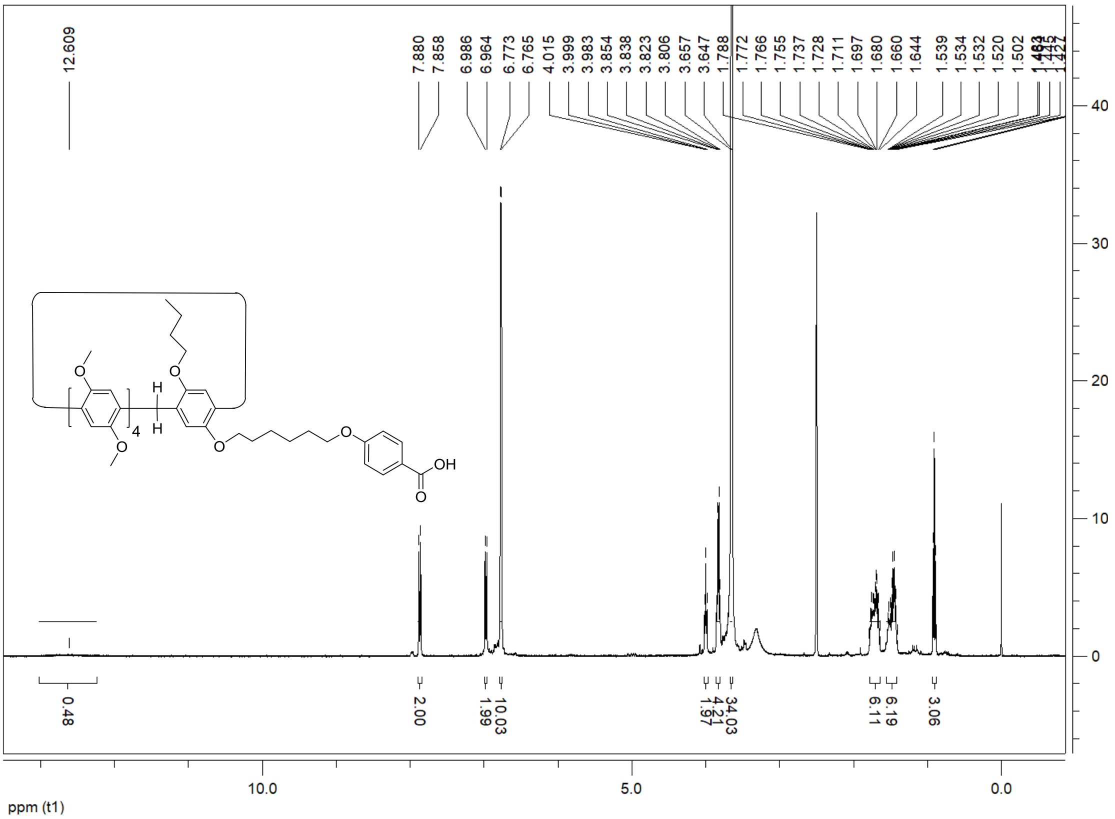

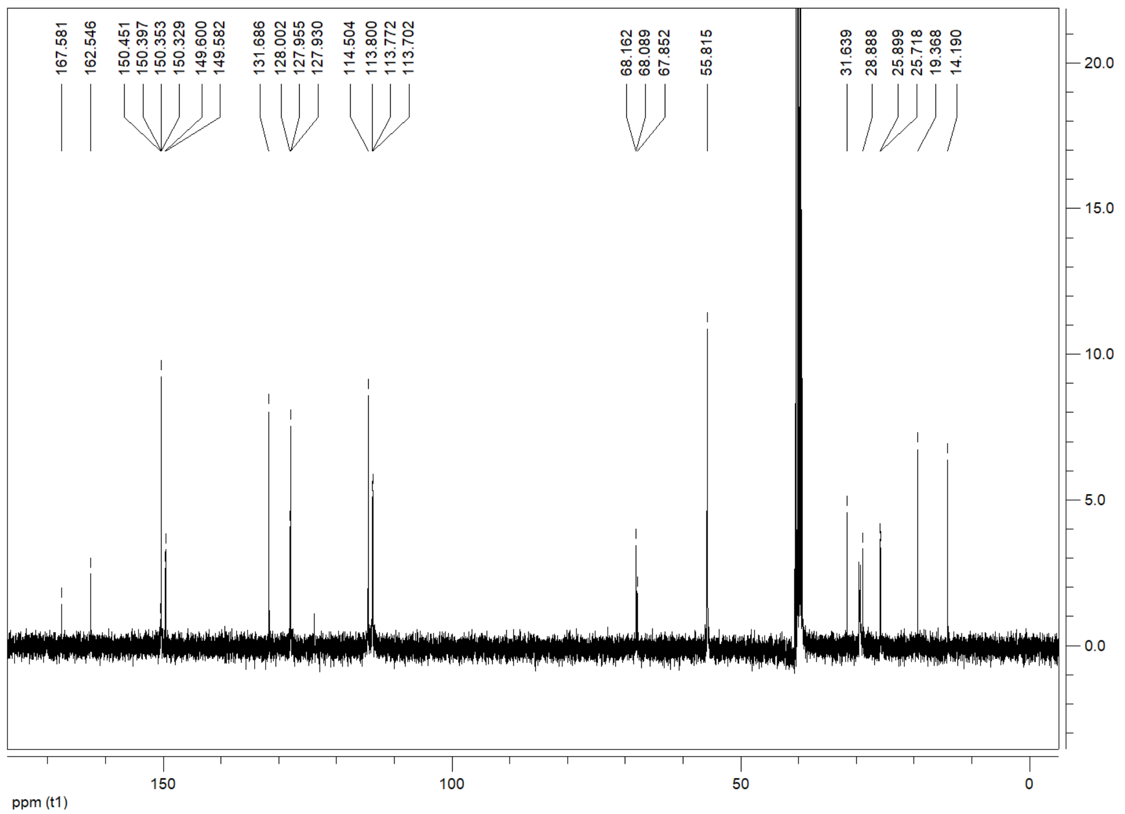
167.581
162.546
150.451
150.397
150.353
150.329
149.600
149.582
131.686
128.002
127.955
127.930
114.504
113.800
113.772
113.702
68.162
68.089
67.852
55.815
31.639
28.888
25.899
25.718
19.368
14.190
20.0
15.0
10.0
5.0
0.0
150
100
50
0
ppm (t1)

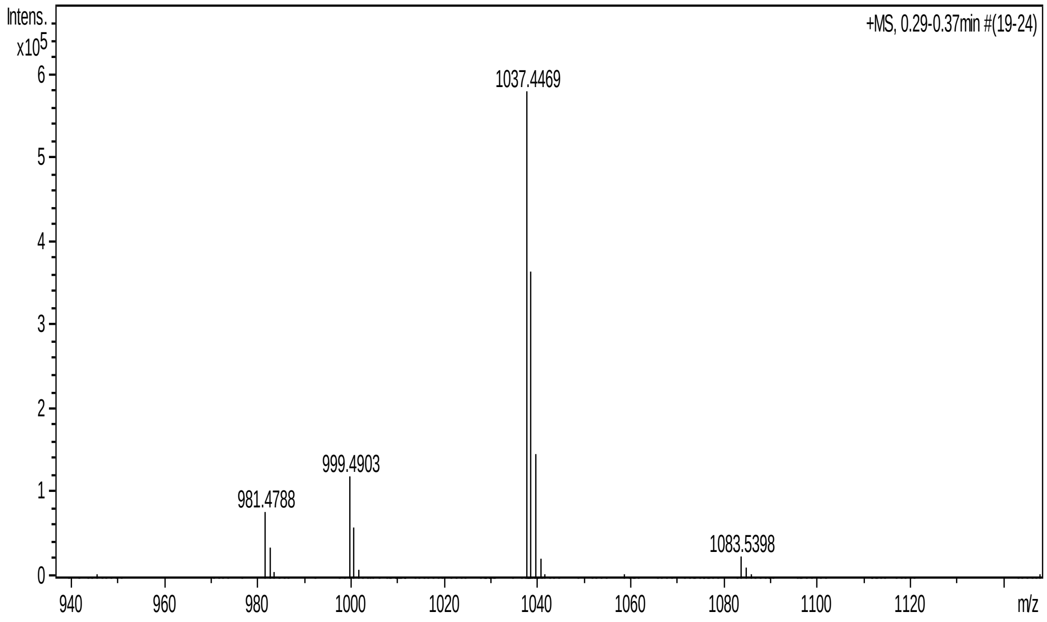
Intens.
x10^5
+MS, 0.29-0.37min #(19-24)
1037.4469
999.4903
981.4788
1083.5398
940
960
980
1000
1020
1040
1060
1080
1100
1120
m/z

5a: White solid, 29%, m.p. 126-128 °C; ^{1}H NMR (600 MHz, $CDCl_3$) δ: 7.51 (d, J = 7.2 Hz, 2H, ArH), 6.99-6.87 (m, 6H, ArH), 6.83-6.67 (m, 16H, ArH), 6.43 (brs, 1H, NH), 4.32 (s, 2H, CH_2), 4.10 (t, J = 6.0 Hz, 2H, CH_2), 4.00 (brs, 1H, NH), 3.93 (t, J = 6.0 Hz, 2H, CH_2), 3.86-3.63 (m, 68H, 10CH_2, 16OCH_3), 3.36 (brs, 2H, CH_2), 2.09-2.04 (m, 2H, CH_2), 2.02-1.97 (m, 2H, CH_2), 1.85-1.81 (m, 2H, CH_2), 1.75-1.70 (m, 2H, CH_2), 1.62 (s, 4H, CH_2), 1.58-1.54 (m, 2H, CH_2), 1.51-1.47 (m, 2H, CH_2), 1.28-1.22 (m, 2H, CH_2), 0.99 (t, J = 7.2 Hz, 3H, CH_3), 0.94 (t, J = 7.2 Hz, 3H, CH_3); ^{13}C NMR (100 MHz, $CDCl_3$) δ: 161.2, 151.5, 150.9, 150.8, 150.7, 150.6, 150.5, 150.3, 150.2, 150.1, 149.7, 148.9, 129.0, 128.7, 128.3, 128.2, 128.1, 128.0, 127.8, 127.7, 127.6, 127.0, 115.0, 114.9, 114.8, 114.6, 114.5, 114.2, 114.1, 114.0, 113.9, 113.8, 113.2, 112.9, 112.8, 68.1, 67.9, 67.7, 66.5, 56.1, 55.9, 55.8, 55.7, 55.4, 55.3, 52.4, 38.1, 31.9, 31.8, 29.7, 29.6, 29.4, 26.5, 26.4, 22.6, 19.4, 14.0, 13.9; IR (KBr) υ: 3397, 2933, 2837, 1667, 1610, 1501, 1460, 1397, 1309, 1210, 1101, 1042, 928, 871, 770, 696 cm^{-1}; MS (m/z): HRMS (ESI) Calcd. for $C_{109}H_{127}N_2O_{23}$ ($[M+H]^+$): 1832.8863, found: 1832.8894.

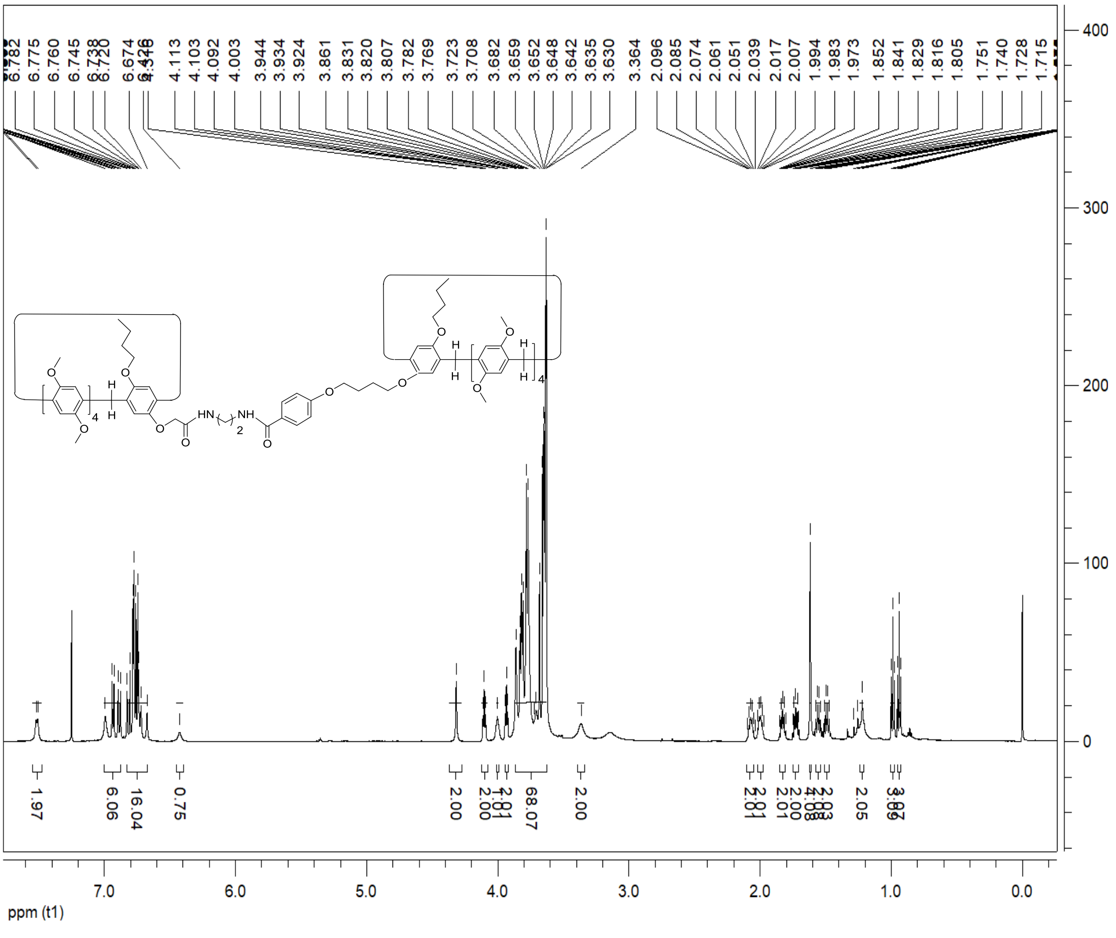

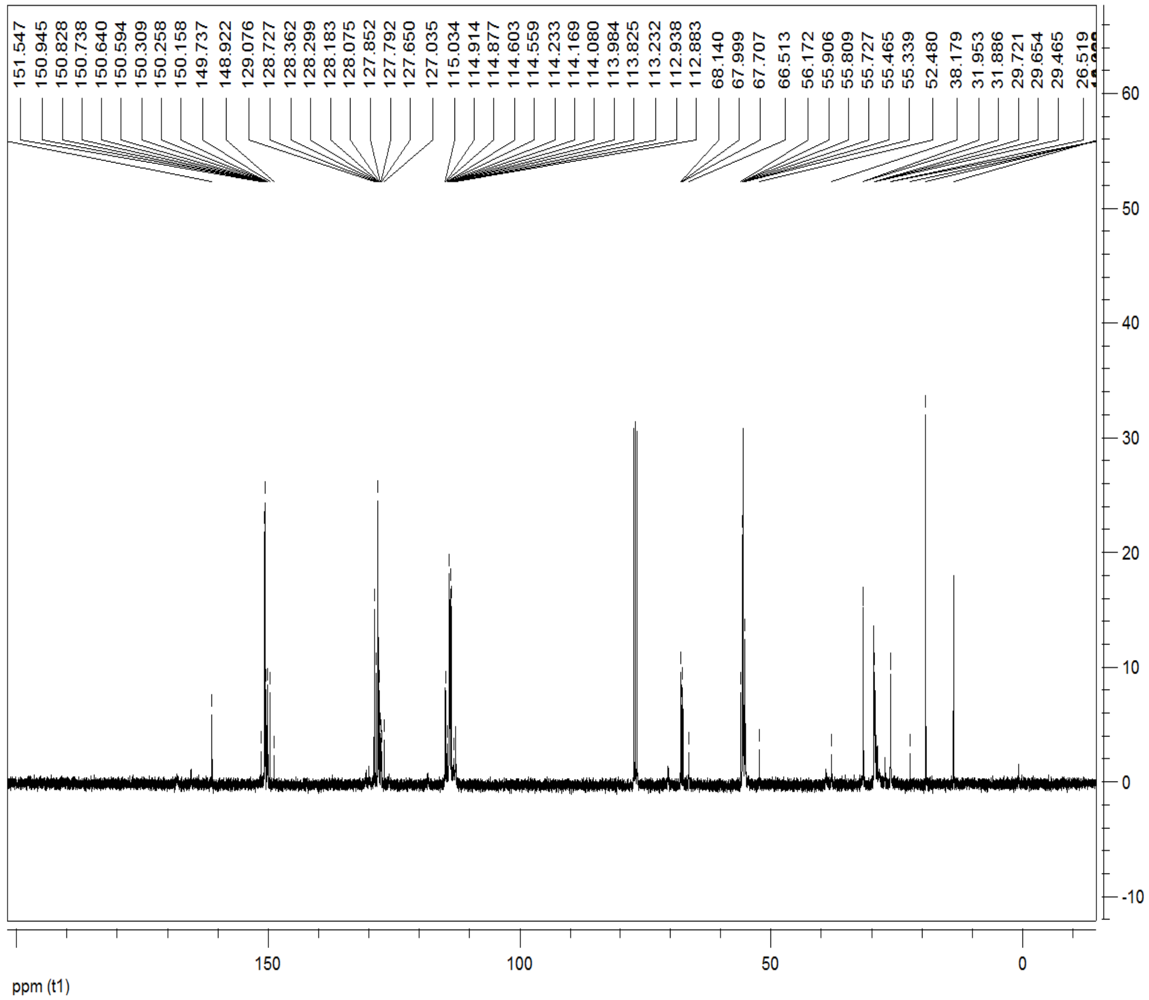

151.547
150.945
150.828
150.738
150.640
150.594
150.309
150.258
150.158
149.737
148.922
129.076
128.727
128.362
128.299
128.183
128.075
127.852
127.792
127.650
127.035
115.034
114.914
114.877
114.603
114.559
114.233
114.169
114.080
113.984
113.825
113.232
112.938
112.883
68.140
67.999
67.707
66.513
56.172
55.906
55.809
55.727
55.465
55.339
52.480
38.179
31.953
31.886
29.721
29.654
29.465
26.519
ppm (t1)

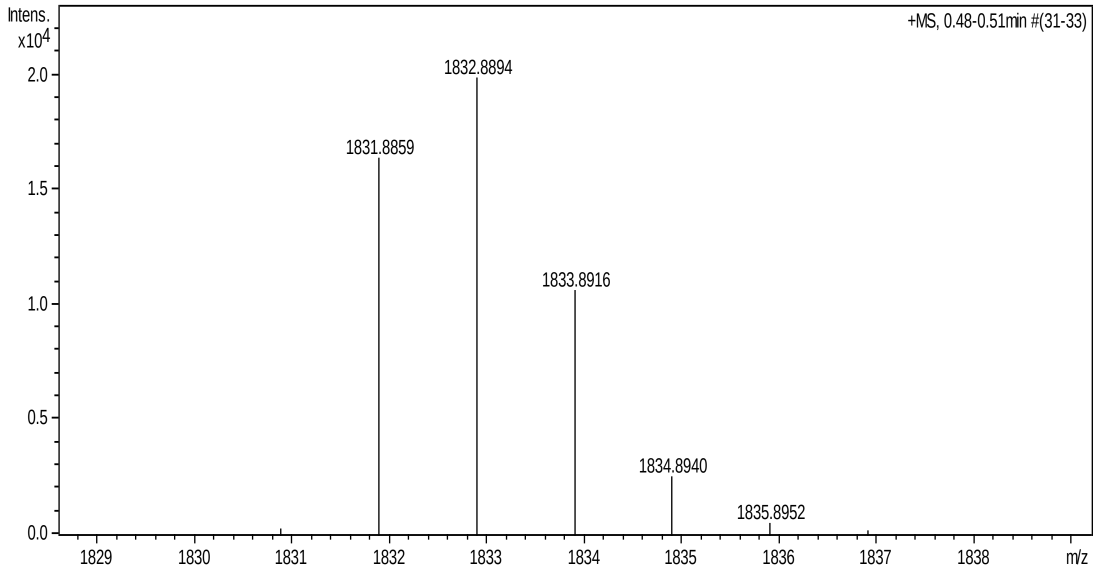

Intens.
x10^4
+MS, 0.48-0.51min #(31-33)
1832.8894
1831.8859
1833.8916
1834.8940
1835.8952
m/z

5b: White solid, 44%, m.p. 136-138 °C; ^{1}H NMR (600 MHz, CDCl$_3$) δ: 7.71 (d, *J* = 8.4 Hz, 2H, ArH), 6.97-6.75 (m, 22H, ArH), 6.51 (brs, 1H, NH), 4.43 (brs, 1H, NH), 4.11 (t, *J* = 6.0 Hz, 2H, CH$_2$), 3.98-3.92 (m, 4H, CH$_2$), 3.89 (brs, 2H, CH$_2$), 3.84-3.64 (m, 68H, 10CH$_2$, 16OCH$_3$), 3.57 (brs, 2H, CH$_2$), 3.32 (brs, 2H, CH$_2$), 2.09-2.06 (m, 2H, CH$_2$), 2.01-1.99 (m, 2H, CH$_2$), 1.81-1.79 (m, 2H, CH$_2$), 1.79-1.72 (m, 2H, CH$_2$), 1.55-1.48 (m, 6H, CH$_2$), 0.98 (t, *J* = 7.2 Hz, 3H, CH$_3$), 0.95 (t, *J* = 7.2 Hz, 3H, CH$_3$), -1.82 (brs, 2H, CH$_2$); ^{13}C NMR (100 MHz, CDCl$_3$) δ: 161.0, 150.8, 150.7, 150.6, 150.5, 150.4, 150.3, 150.2, 149.7, 148.4, 128.8, 128.6, 128.3, 128.2, 128.1, 128.0, 114.9, 114.8, 114.1, 114.0, 113.9, 68.1, 67.9, 67.7, 55.8, 55.7, 55.6, 36.0, 35.1, 31.9, 31.8, 29.7, 29.6, 29.5, 29.4, 29.3, 26.4, 26.3, 22.6, 19.4, 14.1, 14.0, 13.9; IR (KBr) υ: 3401, 2931, 2857, 1663, 1611, 1501, 1460, 1396, 1307, 1210, 1042, 928, 873, 770 cm^{-1}; MS (m/z): HRMS (ESI) Calcd. for $C_{110}H_{129}N_2O_{23}$ ([M+H]$^+$): 1846.9020, found: 1846.9072.

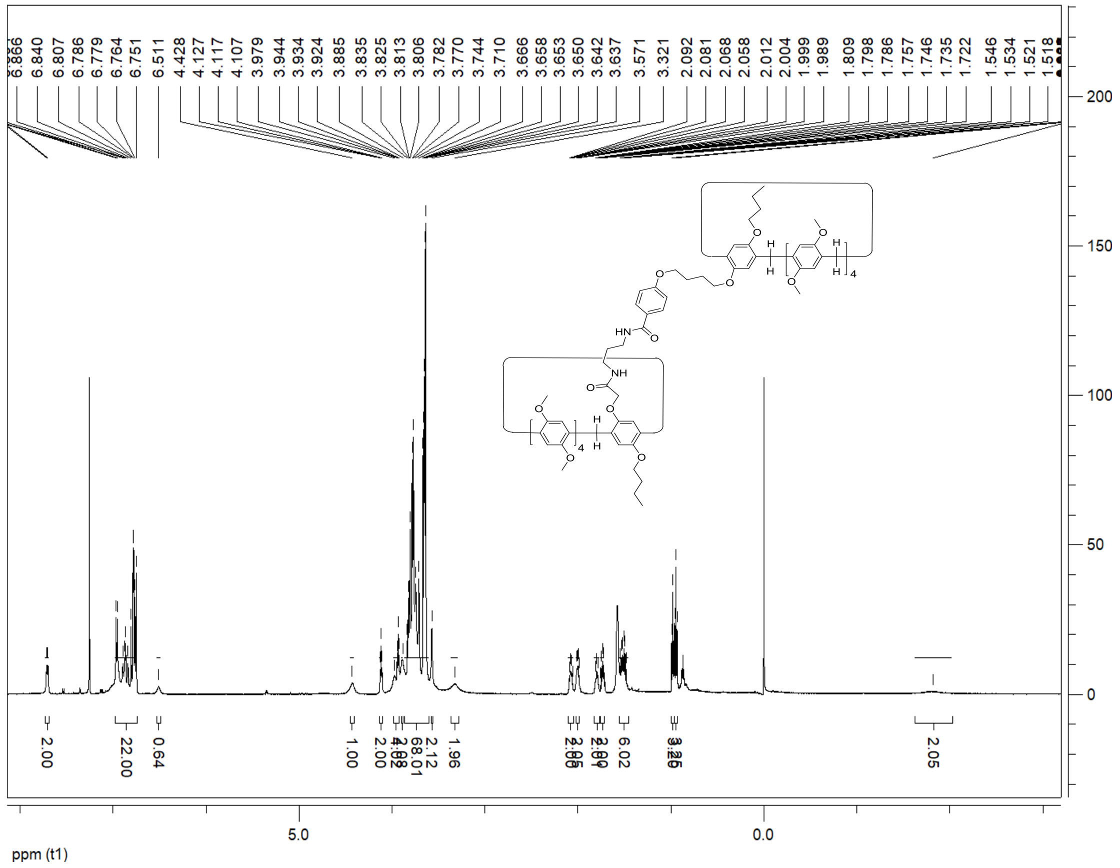

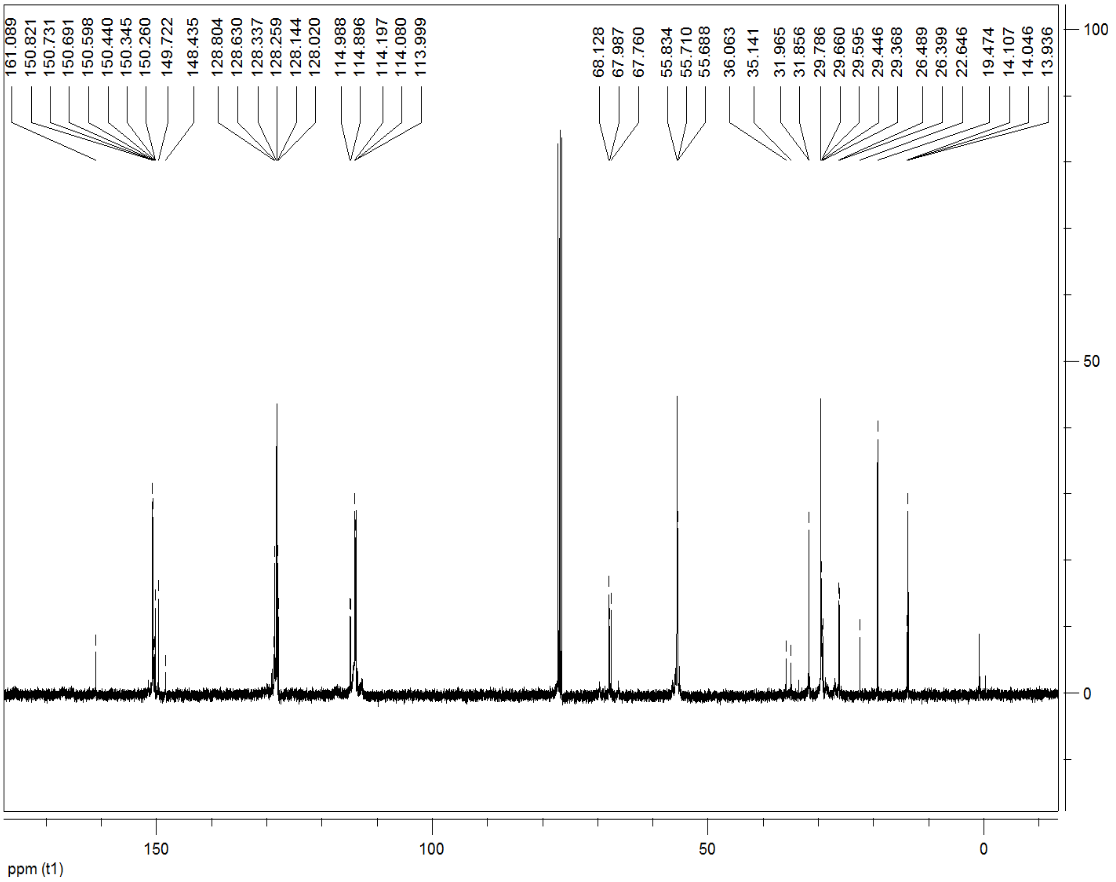
161.089
150.821
150.731
150.691
150.598
150.440
150.345
150.260
149.722
148.435
128.804
128.630
128.337
128.259
128.144
128.020
114.988
114.896
114.197
114.080
113.999
68.128
67.987
67.760
55.834
55.710
55.688
36.063
35.141
31.965
31.856
29.786
29.660
29.595
29.446
29.368
26.489
26.399
22.646
19.474
14.107
14.046
13.936
100
50
0
150
100
50
0
ppm (t1)

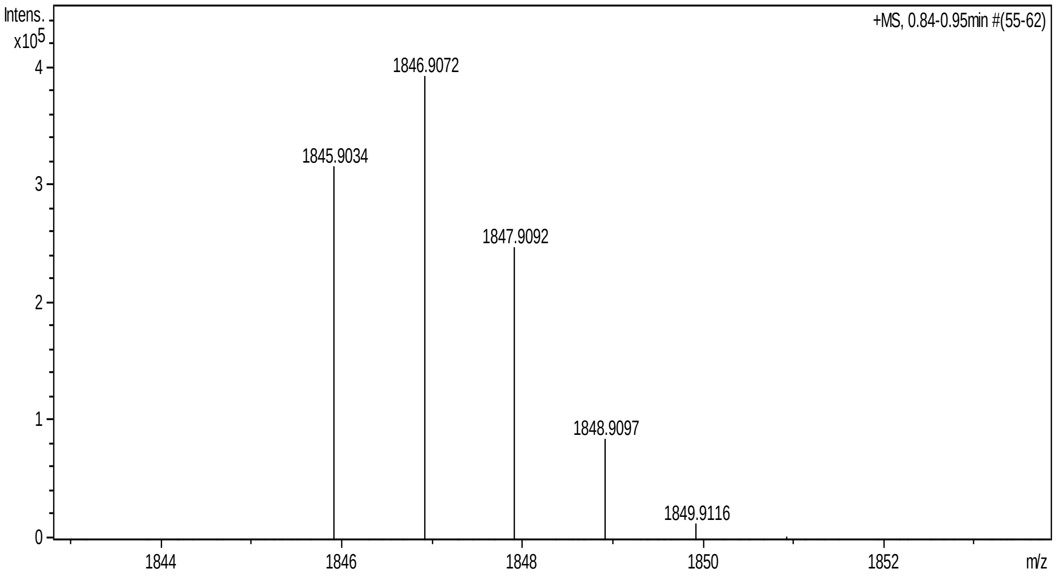
Intens.
x10^5
+MS, 0.84-0.95min #(55-62)
1846.9072
1845.9034
1847.9092
1848.9097
1849.9116
1844
1846
1848
1850
1852
m/z

5c: White solid, 16%, m.p. 130-132 °C; ^1H NMR (600 MHz, $CDCl_3$) δ: 7.85 (d, *J* = 8.4 Hz, 2H, ArH), 7.02-6.96 (m, 8H, ArH), 6.90 (s, 1H, ArH), 6.84-6.75 (m, 12H, ArH), 6.61 (s, 1H, ArH), 5.25 (brs, 1H, NH), 4.62 (brs, 1H, NH), 4.54 (d, *J* = 5.4 Hz, 2H, CH_2), 4.14 (t, *J* = 6.0 Hz, 2H, CH_2), 3.94 (t, *J* = 6.0 Hz, 2H, CH_2), 3.87-3.63 (m, 68H, $10CH_2$, $16OCH_3$), 3.53 (s, 2H, CH_2), 2.10-2.08 (m, 2H, CH_2), 2.02-2.00 (m, 2H, CH_2), 1.74-1.72 (m, 2H, CH_2), 1.59 (s, 6H, CH_2), 1.51-1.49(m, 2H, CH_2), 1.47-1.43(m, 2H, CH_2), 1.24(m, 2H, CH_2), 0.95-0.91 (m, 6H, CH_3), -1.88- -2.14 (m, 4H, CH_2); ^{13}C NMR (100 MHz, $CDCl_3$) δ: 166.5, 161.2, 150.8, 150.7, 150.6, 150.5, 150.4, 150.2, 150.1, 150.0, 149.8, 149.7, 148.4, 131.9, 129.8, 129.0, 128.7, 128.6, 128.4, 128.3, 128.2, 128.1, 128.0, 127.5, 127.4, 126.6, 117.8, 117.0, 114.8, 114.1, 114.0, 113.9, 113.7, 113.1, 113.0, 112.6, 112.4, 68.1, 67.9, 67.8, 66.0, 57.7, 55.8, 55.7, 55.6, 55.4, 55.3, 39.8, 37.2, 31.8, 31.4, 29.7, 29.6, 29.5, 29.4, 28.7, 28.5, 27.4, 26.5, 26.4, 22.8, 22.6, 19.4, 14.0, 13.9; IR (KBr) υ: 3408, 2937, 1670, 1610, 1501, 1460, 1397, 1306, 1210, 1102, 1042, 928, 873, 771, 703 cm^{-1}; MS (m/z): HRMS (ESI) Calcd. for $C_{111}H_{131}N_2O_{23}$ ($[M+H]^+$): 1860.9176, found: 1860.9220.

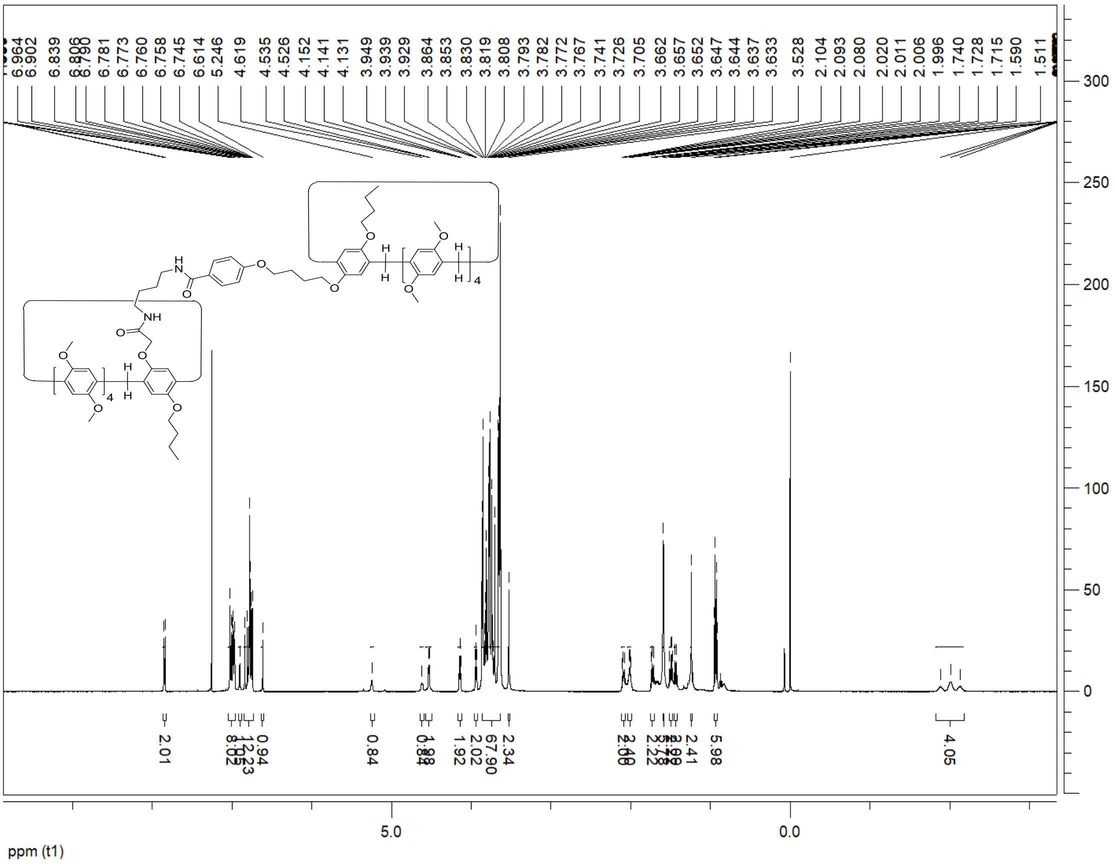

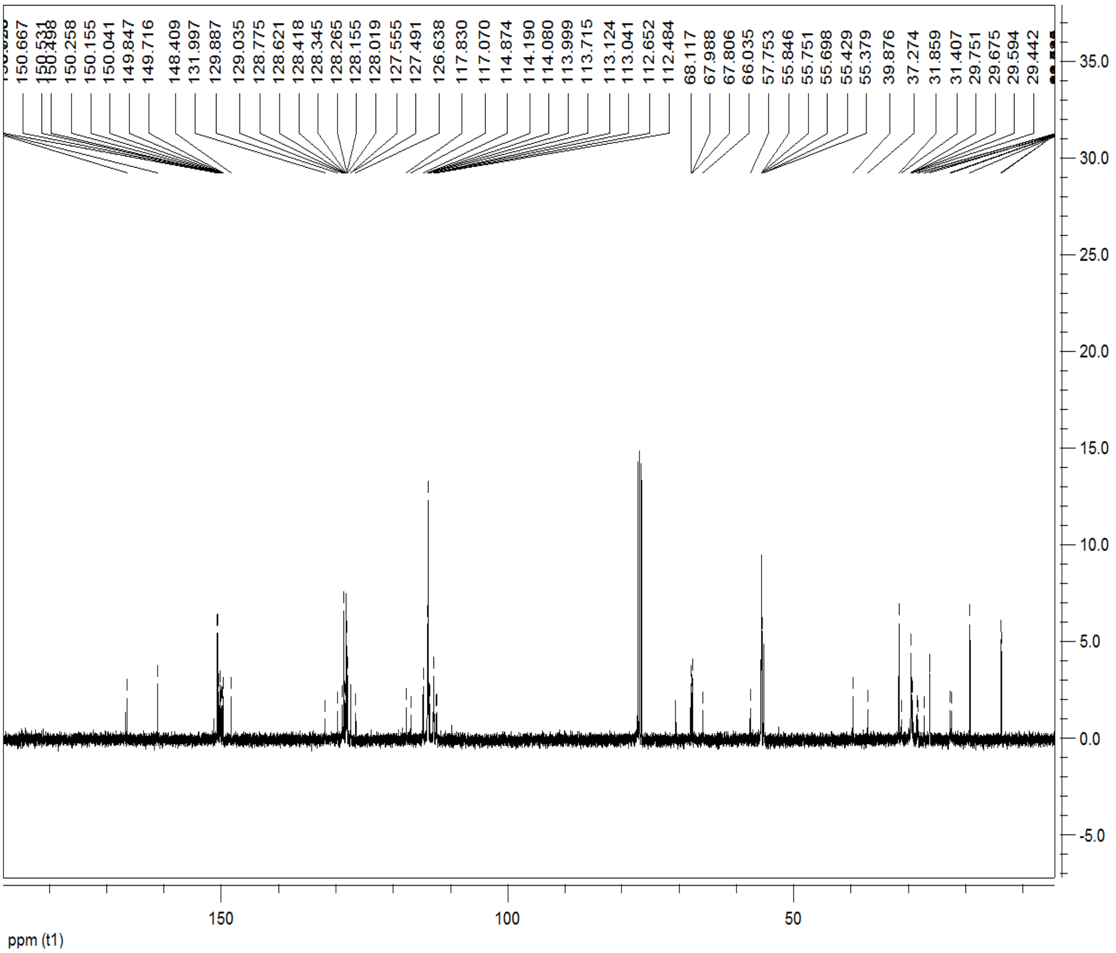

150.667
150.531
150.498
150.258
150.155
150.041
149.847
149.716
148.409
131.997
129.887
129.035
128.775
128.621
128.418
128.345
128.265
128.155
128.019
127.555
127.491
126.638
117.830
117.070
114.874
114.190
114.080
113.999
113.715
113.124
113.041
112.652
112.484
68.117
67.988
67.806
66.035
57.753
55.846
55.751
55.698
55.429
55.379
39.876
37.274
31.859
31.407
29.751
29.675
29.594
29.442
35.0
30.0
25.0
20.0
15.0
10.0
5.0
0.0
-5.0
150
100
50
ppm (t1)

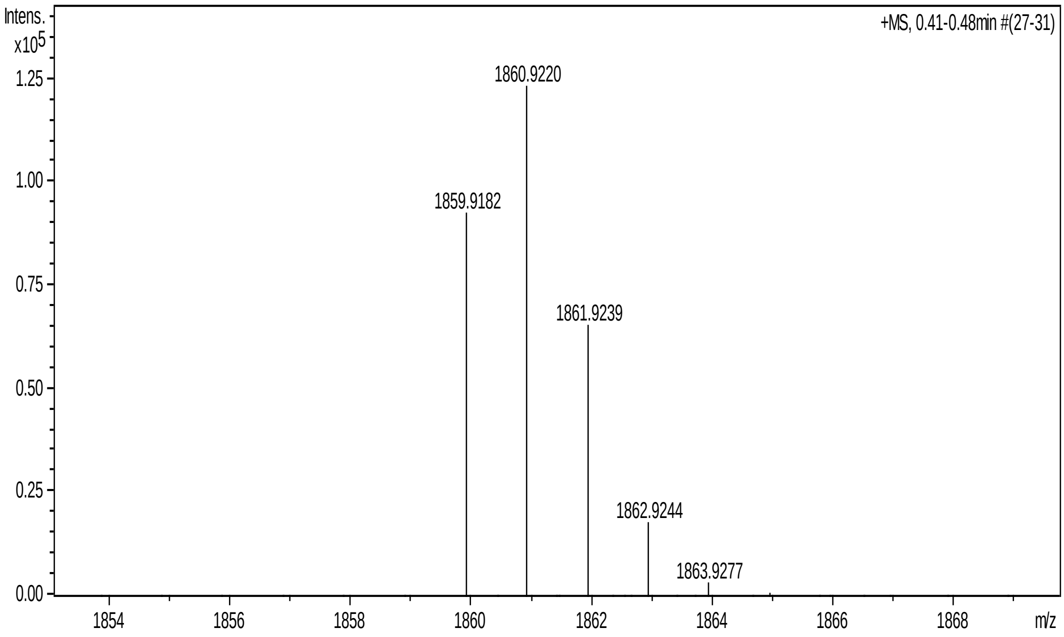

Intens.
x10^5
+MS, 0.41-0.48min #(27-31)
1860.9220
1859.9182
1861.9239
1862.9244
1863.9277
1.25
1.00
0.75
0.50
0.25
0.00
1854
1856
1858
1860
1862
1864
1866
1868
m/z

5d: White solid, 8%, m.p. 129-131 °C; White solid, 16%, m.p. 130-132 °C; ^{1}H NMR (600 MHz, $CDCl_3$) δ: 7.85 (d, *J* = 8.4 Hz, 2H, ArH), 7.01-6.74 (m, 22H, ArH), 6.13 (brs, 1H, NH), 5.28 (brs, 1H, NH), 4.59 (d, *J* = 5.4 Hz, 2H, CH_2), 4.12 (t, *J* = 6.0 Hz, 2H, CH_2), 3.92 (t, *J* = 6.0 Hz, 2H, CH_2), 3.83-3.73 (m, 48H, 16OCH_3), 3.66-3.60 (m, 20H, 10CH_2), 3.52 (s, 2H, CH_2), 2.09-2.04 (m, 2H, CH_2), 1.99-1.97 (m, 2H, CH_2), 1.74-1.70 (m, 2H, CH_2), 1.62-1.58 (m, 8H, CH_2), 1.51-1.45 (m, 4H, CH_2), 1.24-1.23 (m, 6H, CH_2), 0.95-0.89 (m, 6H, CH_3), 0.07 (s, 2H, CH_2), -0.28 (s, 2H, CH_2), -0.72- -0.97 (m, 2H, CH_2), -1.58- -1.66 (m, 2H, CH_2), -2.07 (s, 2H, CH_2); ^{13}C NMR (100 MHz, $CDCl_3$) δ: 167.6, 166.5, 161.4, 150.9, 150.8, 150.7, 150.6, 150.5, 150.4, 150.3, 150.2, 150.1, 149.7, 147.2, 129.6, 129.3, 128.9, 128.6, 128.5, 128.4, 128.3, 128.2, 128.1, 128.0, 127.9, 127.8, 127.5, 127.1, 115.8, 115.2, 115.0, 114.9, 114.8, 114.3, 114.1, 114.0, 113.9, 112.8, 112.7, 112.2, 68.9, 68.1, 67.9, 67.8, 67.7, 65.9, 57.0, 56.3, 55.9, 55.8, 55.7, 55.6, 55.5, 55.4, 55.3, 55.1, 40.0, 37.9, 31.9, 31.8, 30.1, 29.7, 29.6, 29.5, 29.4, 28.9, 28.8, 28.6, 28.5, 26.4, 26.3, 26.2, 25.9, 24.2, 24.1, 23.4, 22.6, 19.4, 14.1, 13.9; IR (KBr) υ: 3411, 2934, 2858, 1668, 1609, 1501, 1460, 1397, 1306, 1210, 1100, 1043, 929, 872, 770, 704 cm^{-1}; MS (m/z): HRMS (ESI) Calcd. for $C_{113}H_{135}N_2O_{23}$ ($[M+H]^+$): 1888.9489, found: 1888.9523.

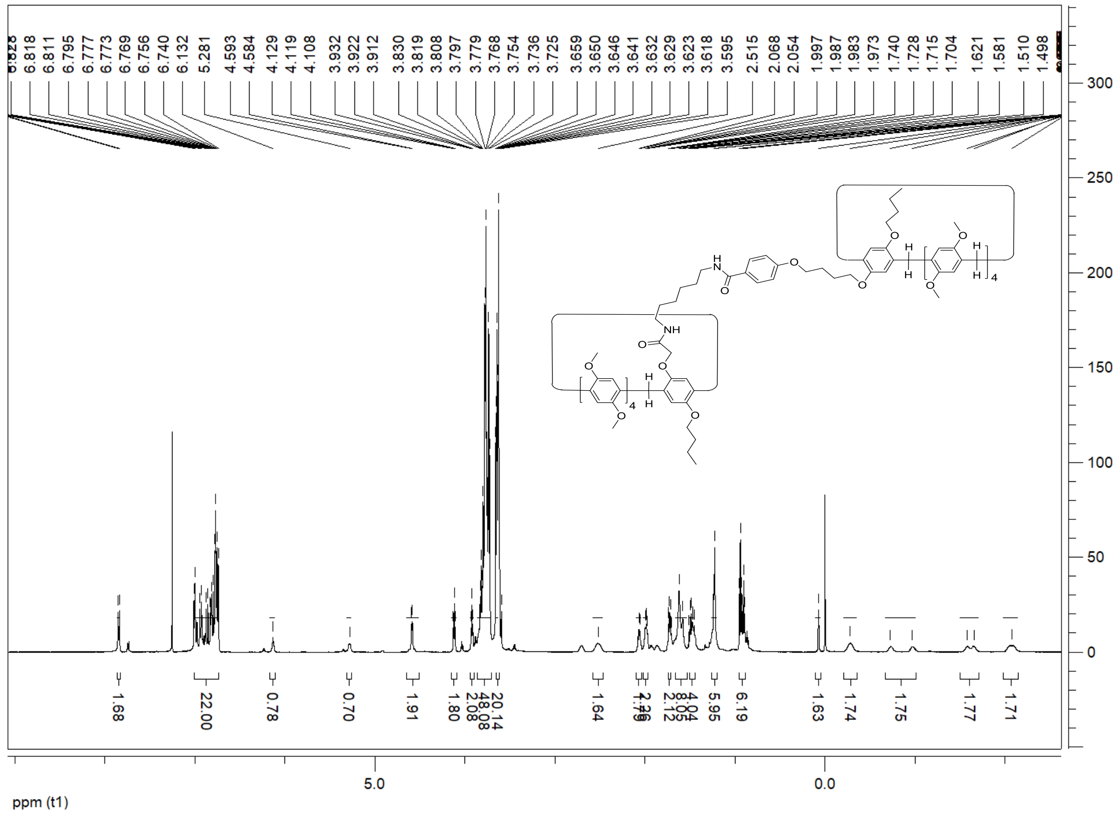

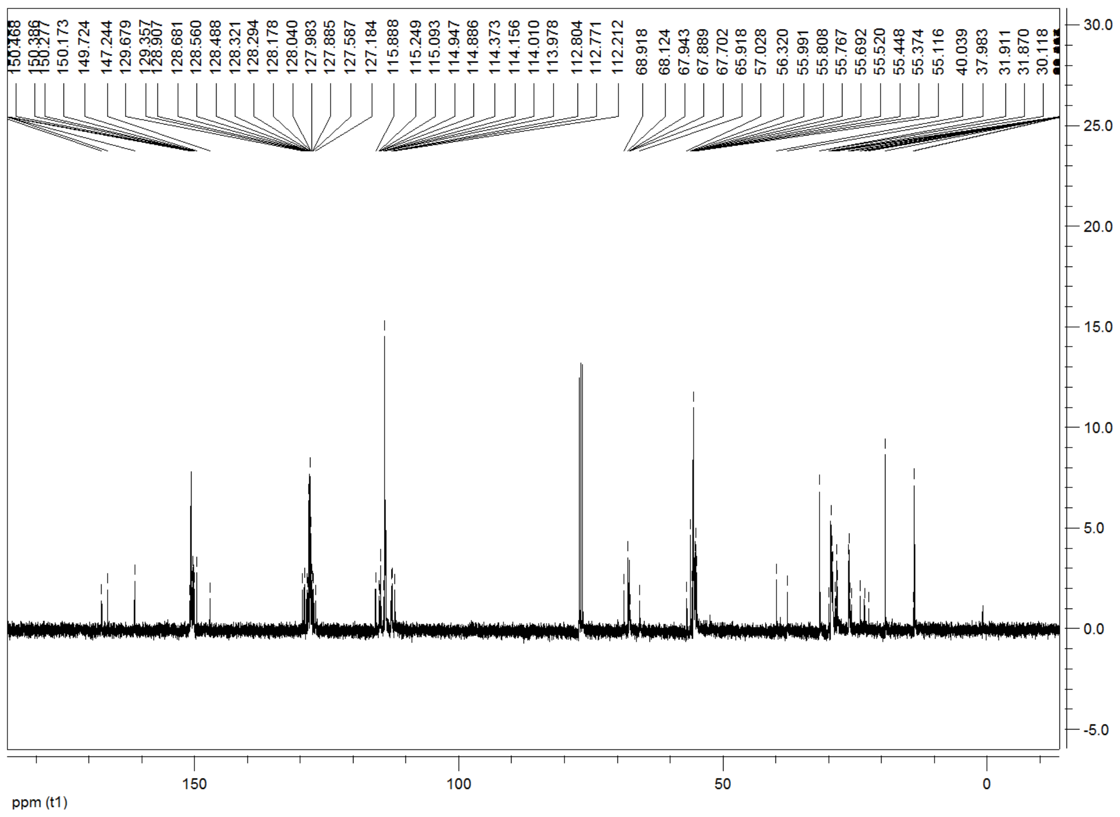
ppm (t1)

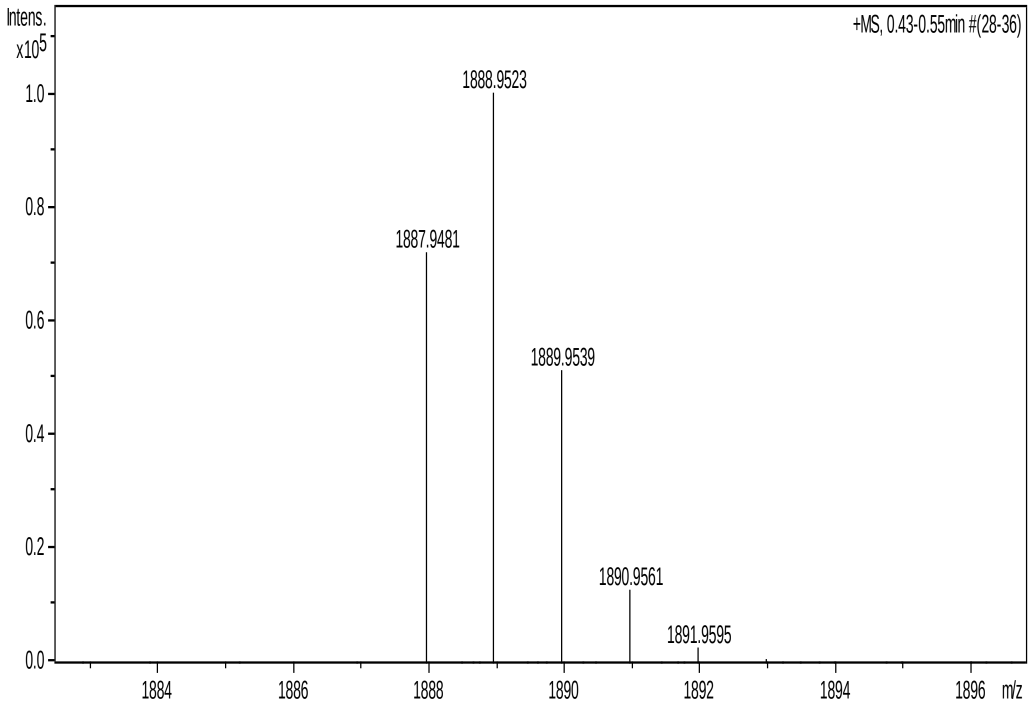
Intens.
x10^5
+MS, 0.43-0.55min #(28-36)
1888.9523
1887.9481
1889.9539
1890.9561
1891.9595
m/z

7: White solid, 20%, m.p. 84.5-86.3 °C; ^{1}H NMR (400 MHz, $CDCl_3$) δ: 7.87 (d, *J* = 4 Hz, 4H, ArH), 6.87 (d, *J* = 4 Hz, 4H, ArH), 6.77-6.72 (m, 10H, ArH), 4.99 (t, *J* = 6 Hz, 4H, OCH_2), 3.89-3.88 (m, 10H, 4OCH_2, 6OCH_3), 3.77-3.75 (m, 10H, CH_2), 3.64-3.57 (m, 24H, OCH_3), 1.94-1.88 (m, 8H, CH_2); ^{13}C NMR (100 MHz, $CDCl_3$) δ: 166.8, 162.7, 150.7, 150.7, 150.7, 150.6, 149.8, 131.5, 128.3, 128.3, 128.3, 128.2, 128.1, 122.3, 114.9, 114.1, 114.1, 114.0, 114.0, 113.8, 113.8, 67.9, 67.6, 60.4, 55.8, 55.8, 55.7, 52.6, 51.8, 29.6, 29.5, 26.2, 25.9, 21.0, 14.1; IR (KBr) υ: 3435, 2936, 2829, 1715, 1606, 1580, 1501, 1466, 1436, 1399, 1281, 1256, 1212, 1168, 1103, 1046, 1010, 975, 930, 880, 849, 772, 732 cm^{-1}; MS (m/z): HRMS (ESI) Calcd. for $C_{67}H_{74}O_{16}Na$ ($[M+Na]^+$): 1157.4875, found: 1157.4856.

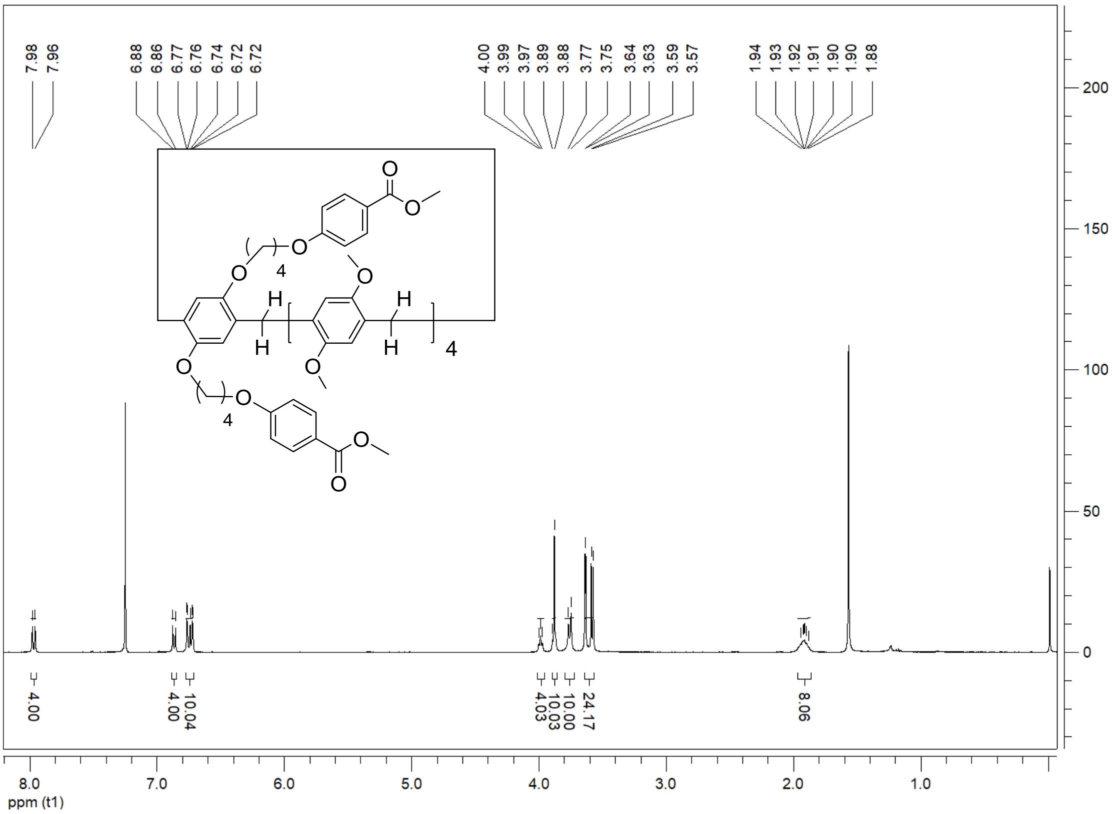

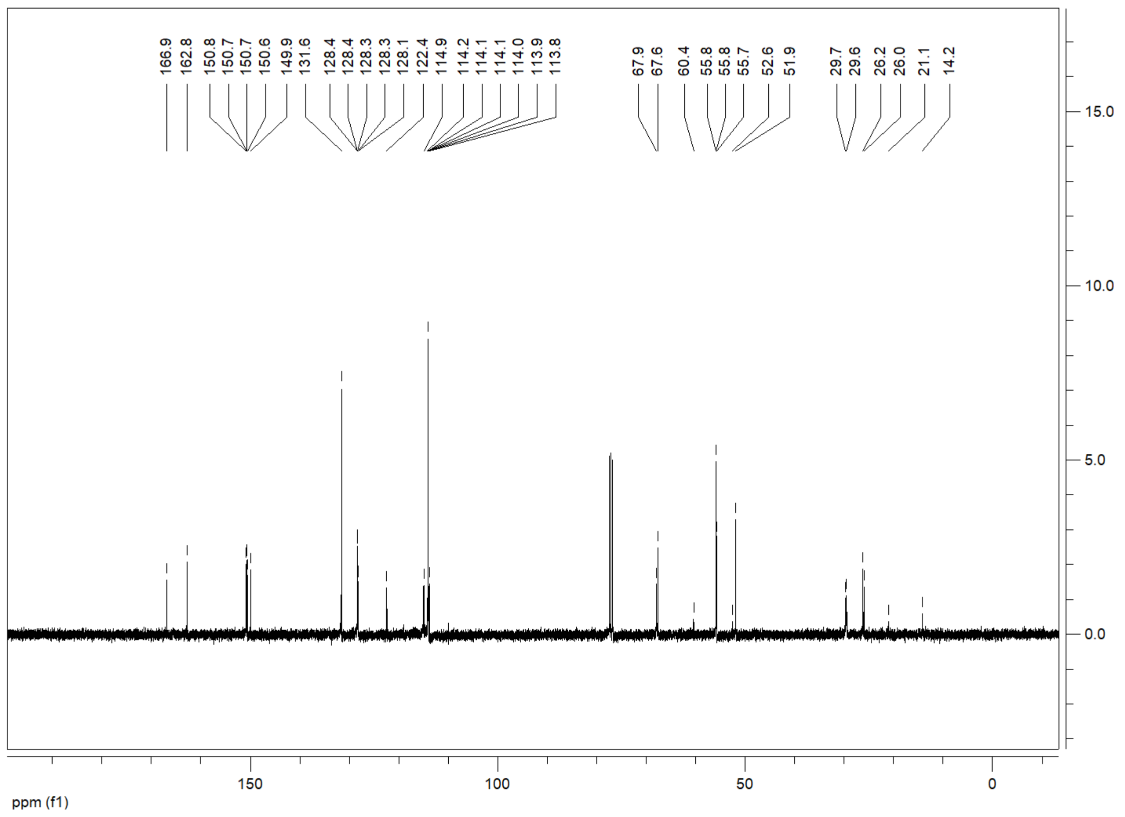
166.9
162.8
150.8
150.7
150.7
150.6
149.9
131.6
128.4
128.4
128.3
128.3
128.1
122.4
114.9
114.2
114.1
114.1
114.0
113.9
113.8
67.9
67.6
60.4
55.8
55.8
55.7
52.6
51.9
29.7
29.6
26.2
26.0
21.1
14.2
15.0
10.0
5.0
0.0
150
100
50
0
ppm (f1)

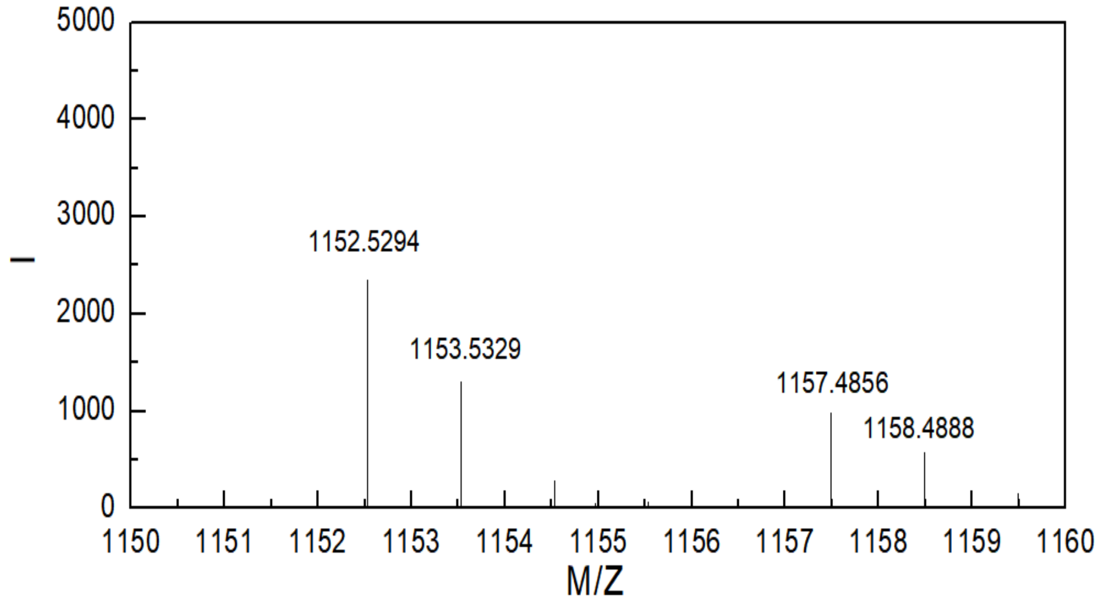
5000
4000
3000
2000
1000
0
I
1152.5294
1153.5329
1157.4856
1158.4888
1150
1151
1152
1153
1154
1155
1156
1157
1158
1159
1160
M/Z

8: White solid, 85%, m.p. 216.6-218.2 °C; ^{1}H NMR (400 MHz, DMSO-d_6) δ: 12.62(s, 2H, COOH), 7.87 (d, J = 4Hz, 4H, ArH), 6.98(d, J = 4Hz, 4H, ArH), 6.78-6.73 (m, 10H, ArH), 4.09 (s, 4H, CH_2), 3.92-3.57 (m, 36H, $12CH_2$, $24OCH_3$), 3.34 (s, 2H, CH_2), 1.93-1.90 (m, 8H, CH_2); ^{13}C NMR (100 MHz, DMSO-d_6) δ: 167.4, 162.6, 150.3, 150.2, 149.5, 131.7, 128.0, 127.9, 127.9, 127.8, 123.2, 114.5, 114.5, 114.4, 113.7, 113.7, 113.6, 67.9, 67.7, 55.7, 29.4, 29.2, 29.1, 26.1, 25.9; IR (KBr) υ: 3436, 2931, 1682, 1605, 1579, 1501, 1466, 1427, 1400, 1255, 1212, 1168, 1105, 1047, 929, 849, 774 cm^{-1}; MS (m/z): HRMS (ESI) Calcd. for $C_{65}H_{70}O_{16}Na$ ($[M+Na]^+$): 1129.4562, found: 1129.4540.

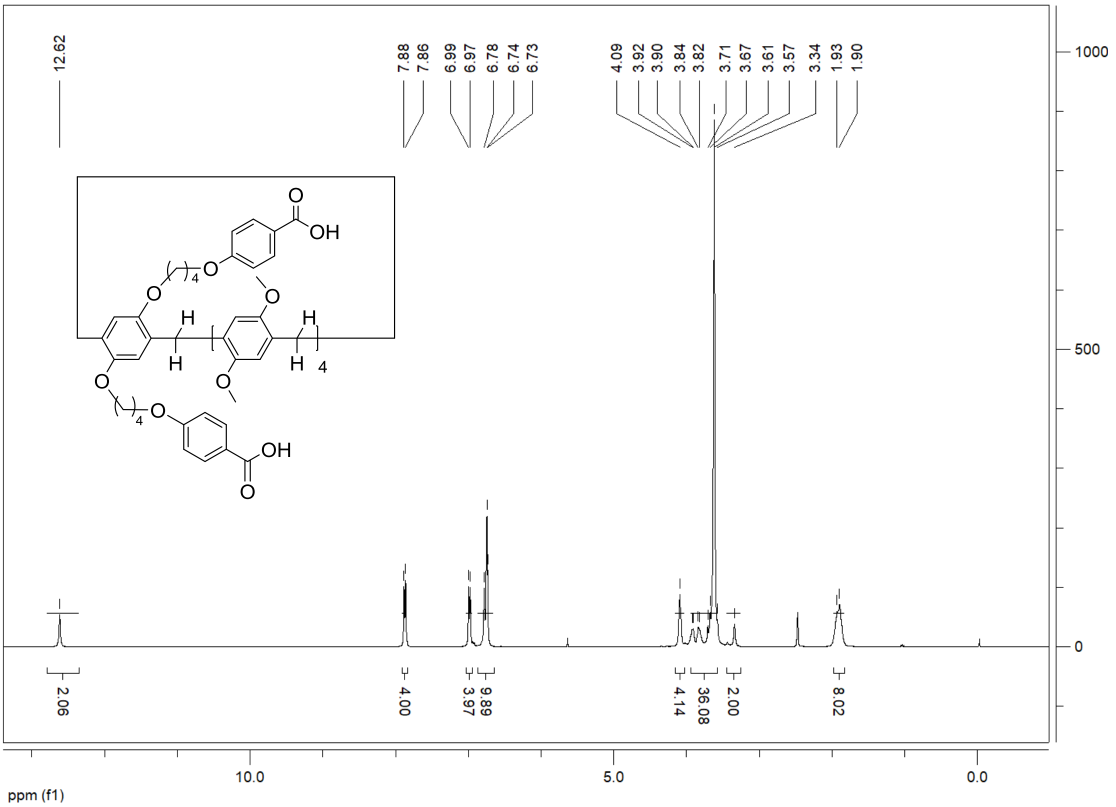

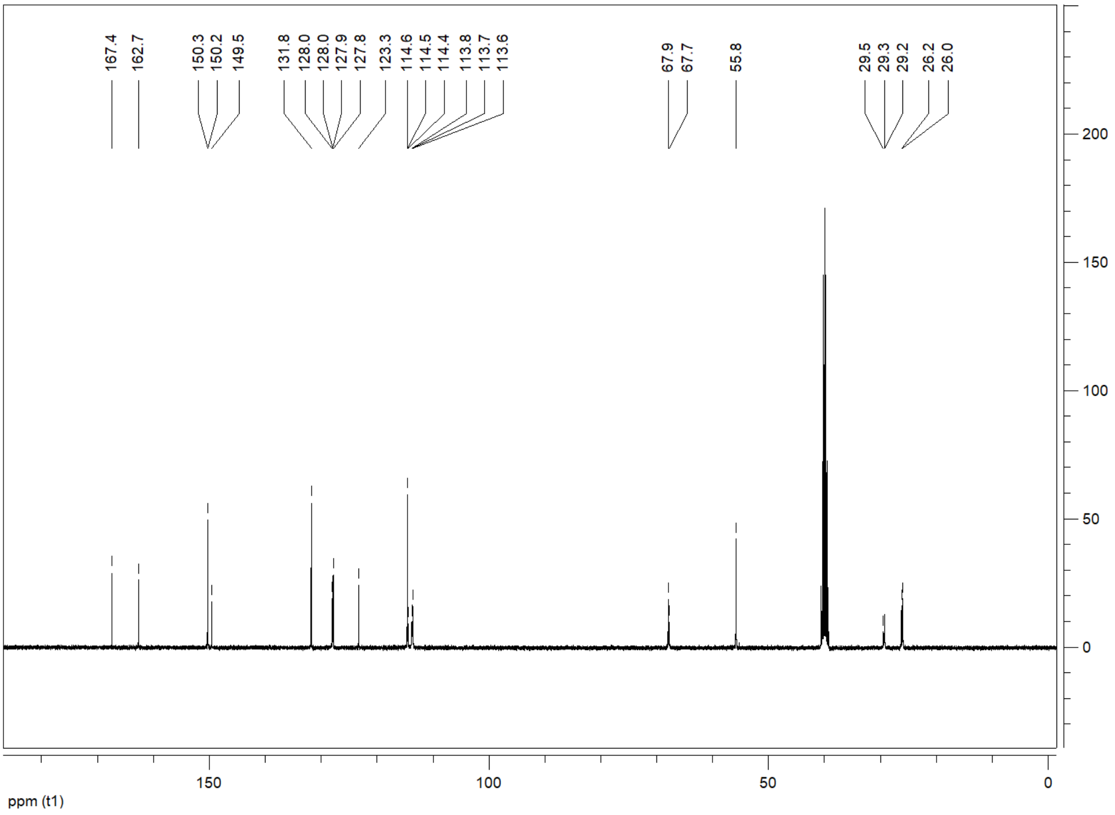
167.4
162.7
150.3
150.2
149.5
131.8
128.0
128.0
127.9
127.8
123.3
114.6
114.5
114.4
113.8
113.7
113.6
67.9
67.7
55.8
29.5
29.3
29.2
26.2
26.0
ppm (t1)

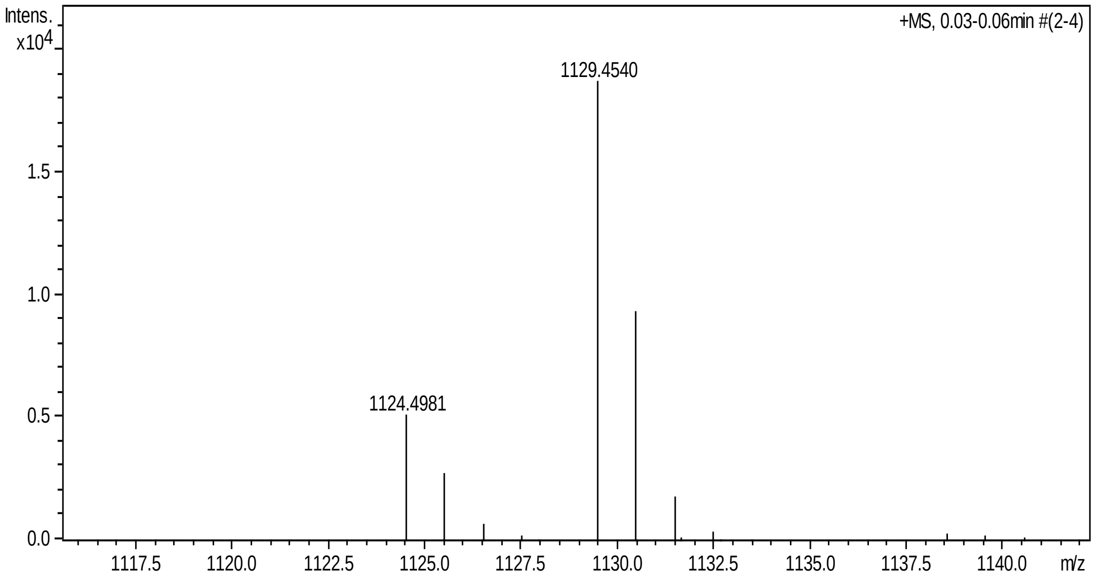
Intens.
x10^4
+MS, 0.03-0.06min #(2-4)
1129.4540
1124.4981
m/z

9a: White solid, 26%, m.p. 129-131 °C; ^{1}H NMR (400 MHz, $CDCl_3$) δ: 7.50 (d, J = 8 Hz, 4H, ArH), 6.96-6.65 (m, 34H, ArH), 6.40 (s, 2H, NH), 5.05 (s, 2H, NH), 4.29 (s, 4H, CH_2), 4.09-3.62 (m, 114H, 42CH_2, 72OCH_3), 3.35 (s, 4H, CH_2), 3.14 (brs, 4H, CH_2), 2.06-1.97 (m, 8H, CH_2), 1.84-1.77 (m, 4H, CH_2), 1.58-1.51 (m, 4H, CH_2), 0.97 (t, J = 7.4Hz, 6H, CH_3); ^{13}C NMR (100 MHz, $CDCl_3$) δ: 165.3, 161.2, 151.5, 150.8, 150.8, 150.8, 150.7, 150.7, 150.6, 150.6, 150.5, 150.3, 150.3, 150.3, 150.2, 150.1, 150.0, 148.8, 130.1, 129.0, 128.7, 128.4, 128.3, 128.3, 128.3, 128.2, 128.1, 128.1, 128.0, 127.8, 127.7, 127.7, 127.6, 127.5, 127.0, 114.9, 114.6, 114.2, 114.2, 114.1, 114.0, 114.0, 113.9, 113.9, 113.7, 113.7, 113.5, 113.4, 113.4, 113.4, 113.3, 113.2, 113.2, 113.2, 113.1, 112.9, 112.8, 68.0, 67.6, 66.4, 56.1, 55.8, 55.8, 55.8, 55.8, 55.7, 55.6, 55.6, 55.5, 55.4, 55.3, 55.3, 55.2, 38.1, 31.9, 29.7, 29.6, 29.1, 27.5, 27.5, 27.5, 26.4, 26.4, 26.3, 19.4, 13.9; IR (KBr) υ: 3397, 2935, 2829, 1662, 1608, 1501, 1463, 1400, 1309, 1250, 1045, 928, 878, 848, 771, 700 cm^{-1}; MS (m/z): HRMS (ESI) Calcd. for $C_{167}H_{190}N_4O_{36}Na$ ($[M+Na]^+$): 2851.3091, found: 2851.3060.

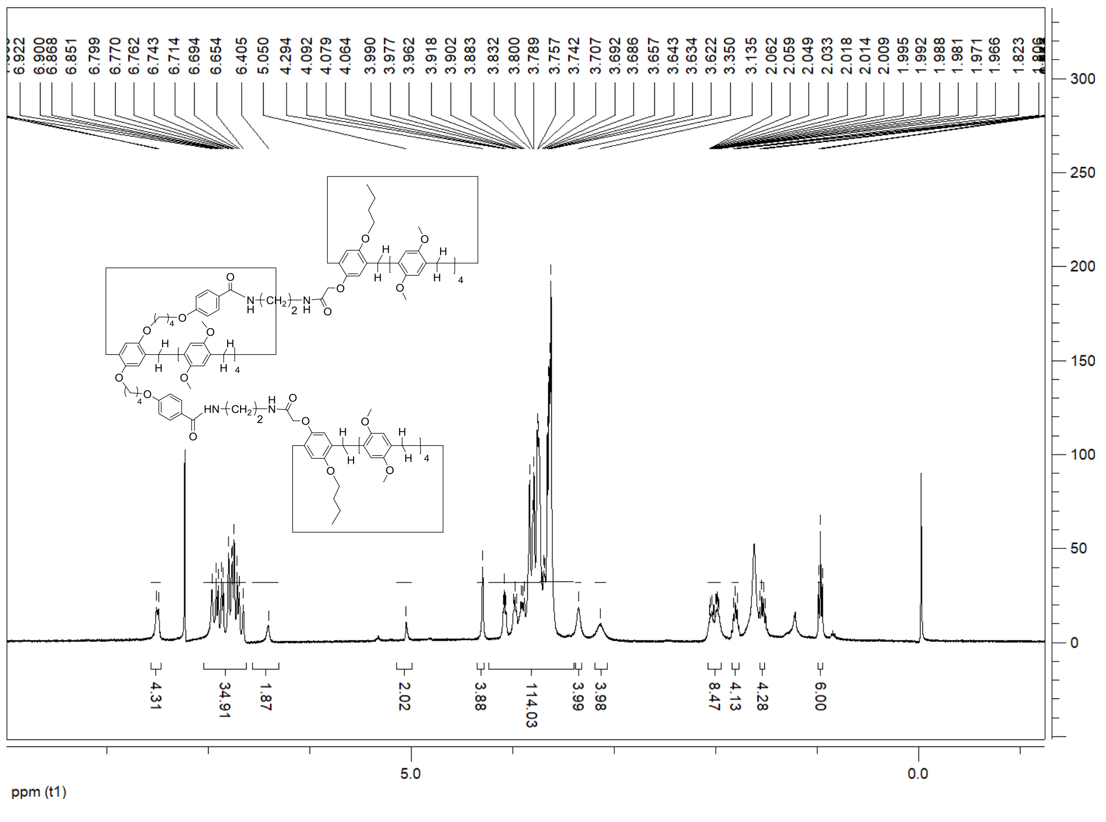

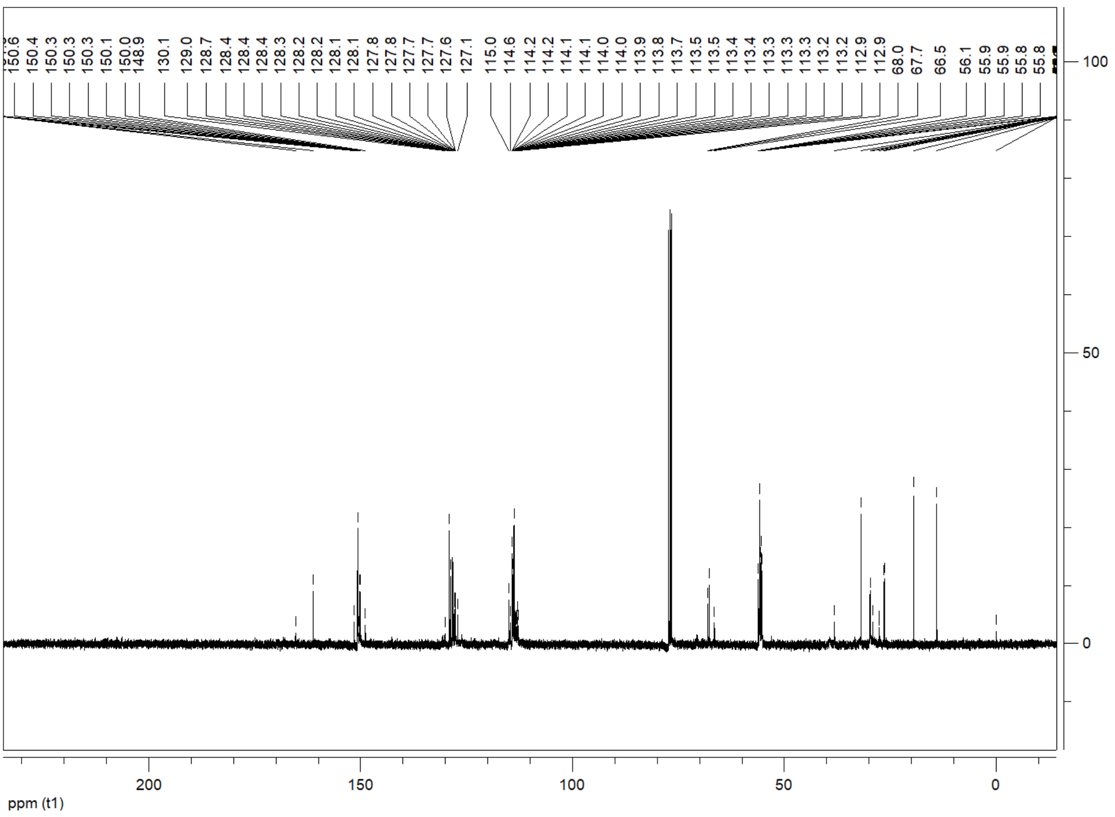

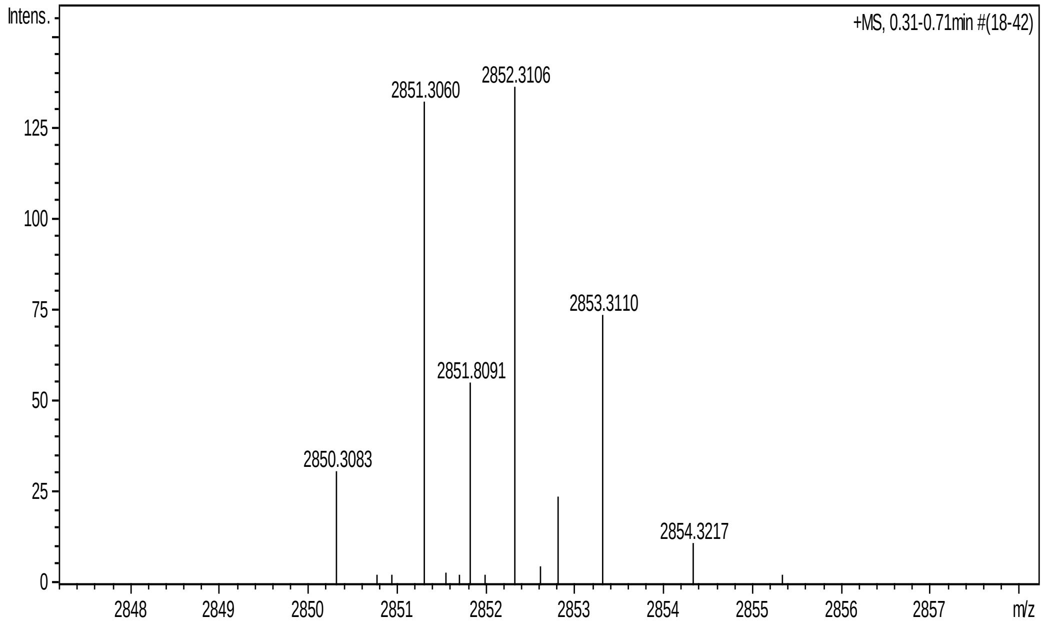

Intens.
+MS, 0.31-0.71min #(18-42)
2851.3060
2852.3106
2853.3110
2851.8091
2850.3083
2854.3217
m/z

9b: White solid, 22%, m.p. 136-138 °C; ^{1}H NMR (400 MHz, $CDCl_3$) δ: 7.72 (d, *J* = 8 Hz, 4H, ArH), 7.01-6.77 (m, 34H, ArH), 6.53 (s, 2H, NH), 4.95 (s, 2H, NH), 4.44 (s, 4H, CH_2), 4.14-3.58 (m, 118H, 46CH_2, 72OCH_3), 3.35(s, 4H, CH_2), 2.10-2.00 (m, 8H, CH_2), 1.84-1.77 (m, 4H, CH_2), 1.59-1.49 (m, 4H, CH_2), 0.99 (t, *J* = 8 Hz, 6H, CH_3), -1.80 (brs, 2H, CH_2); ^{13}C NMR (100 MHz, $CDCl_3$) δ: 161.0, 150.9, 150.8, 150.8, 150.7, 150.6, 150.6, 150.4, 150.3, 150.0, 148.4, 129.1, 128.8, 128.7, 128.7, 128.7, 128.6, 128.6, 128.5, 128.4, 128.4, 128.3, 128.2, 128.1, 128.1, 114.9, 114.2, 114.0, 114.0, 113.9, 68.0, 67.7, 55.8, 55.8, 55.7, 55.3, 36.0, 35.1, 31.9, 29.7, 29.6, 26.5, 26.4, 19.4, 14.0; IR (KBr) υ: 3340, 2935, 2830, 1664, 1608, 1501, 1400, 1309, 1249, 1211, 1044, 828, 878, 849, 771 cm^{-1}; MS (m/z): HRMS (ESI) Calcd. for $C_{169}H_{194}N_4O_{36}Na$ ($[M+Na]^+$): 2879.3404, found: 2879.3399.

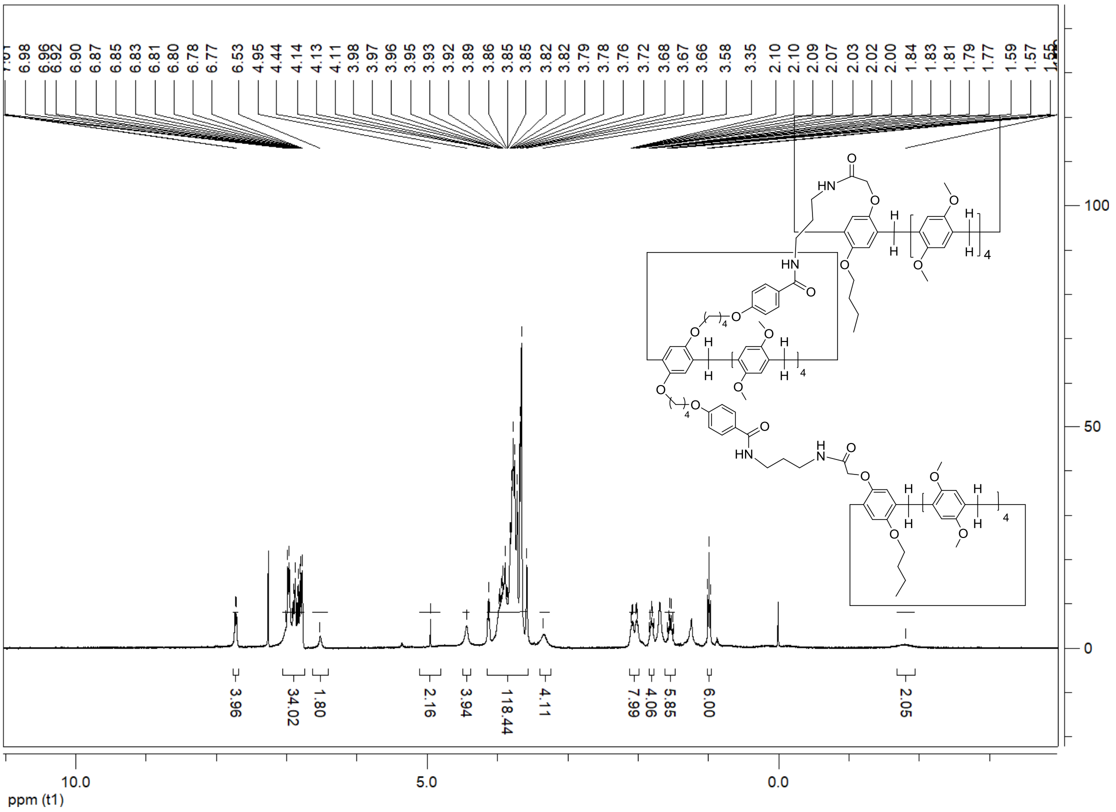

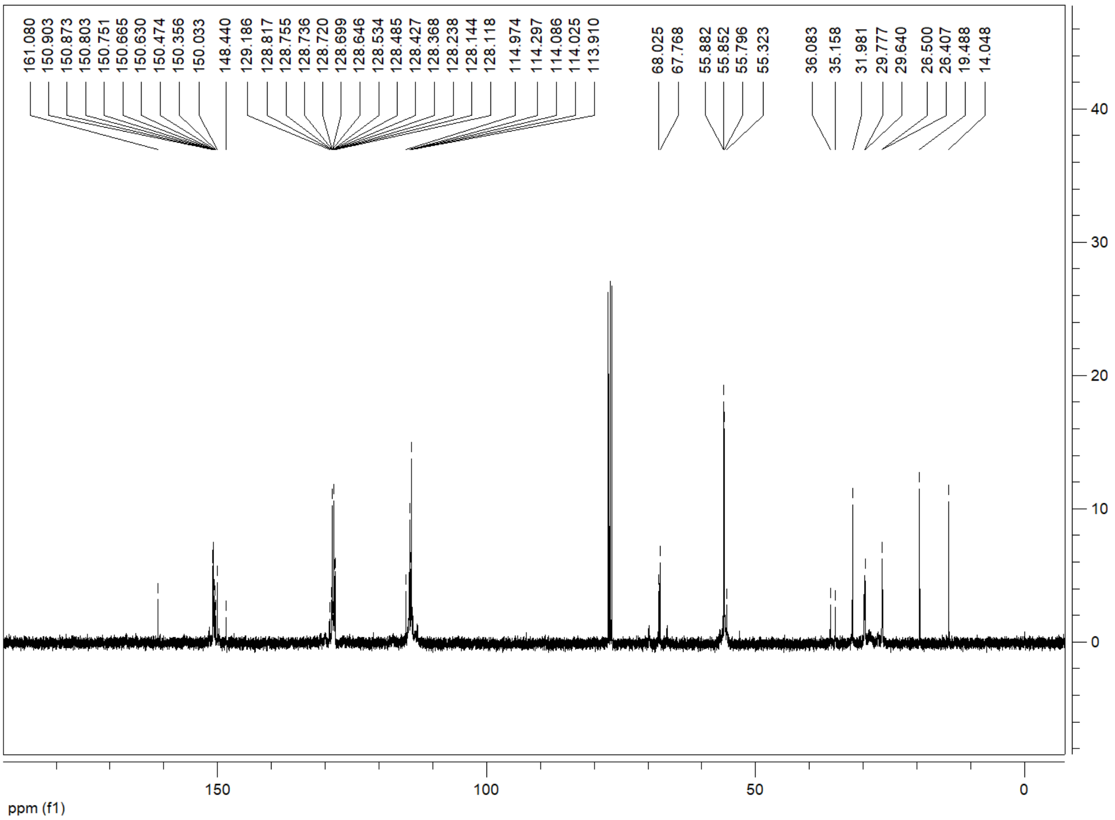
161.080
150.903
150.873
150.803
150.751
150.665
150.630
150.474
150.356
150.033
148.440
129.186
128.817
128.755
128.736
128.720
128.699
128.646
128.534
128.485
128.427
128.368
128.238
128.144
128.118
114.974
114.297
114.086
114.025
113.910
68.025
67.768
55.882
55.852
55.796
55.323
36.083
35.158
31.981
29.777
29.640
26.500
26.407
19.488
14.048
ppm (f1)

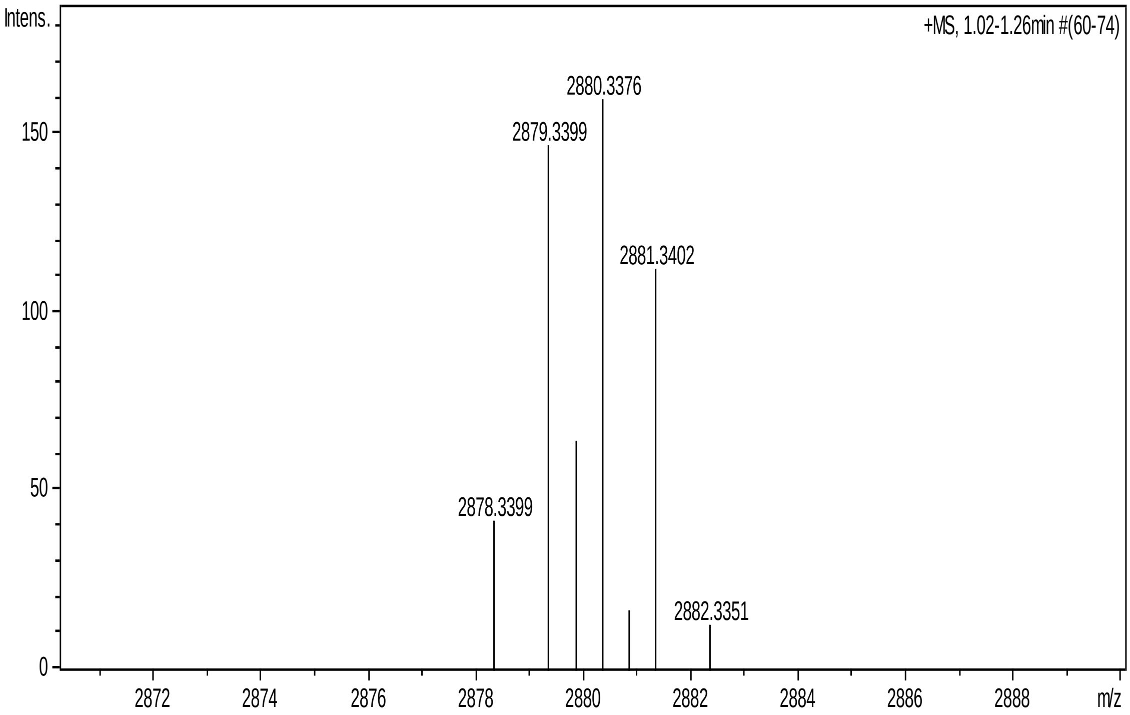
Intens.
+MS, 1.02-1.26min #(60-74)
2880.3376
2879.3399
2881.3402
2878.3399
2882.3351
m/z

9c: White solid, 19%, m.p. 146-148 °C; ^{1}H NMR (400 MHz, $CDCl_3$) δ: 7.86(d, *J* = 6 Hz, 4H, ArH), 7.03-6.77 (m, 34H, ArH), 6.62 (s, 2H, NH), 5.25(s, 2H, NH), 4.54 (s, 4H, CH_2), 4.15(t, *J* = 6 Hz, 4H, CH_2), 3.95-3.53 (m, 118H, 46CH_2, 72OCH_3), 2.10-2.01 (m, 10H, CH_2), 1.47-1.41 (m, 6H, CH_2), 0.93 (t, *J* = 8 Hz, 6H, CH_3), -2.00 (m, 8H, CH_2); ^{13}C NMR (100 MHz, $CDCl_3$) δ: 166.6, 166.4, 161.2, 151.4, 150.9, 150.8, 150.8, 150.7, 150.6, 150.5, 150.5, 150.2, 150.1, 150.0, 149.8, 148.4, 131.9, 129.9, 129.0, 128.7, 128.6, 128.4, 128.3, 128.2, 128.2, 128.1, 128.0, 128.0, 127.5, 127.4, 126.6, 117.8, 117.0, 114.9, 114.3, 114.1, 114.0, 113.9, 113.7, 113.1, 113.1, 113.1, 113.0, 112.9, 112.6, 112.5, 112.4, 70.7, 68.0, 67.7, 66.0, 57.6, 55.8, 55.8, 55.8, 55.7, 55.4, 55.3, 39.8, 37.2, 31.8, 31.3, 29.7, 29.7, 29.6, 28.7, 28.5, 27.4, 26.4, 26.4, 22.9, 22.7, 19.3, 13.9; IR (KBr) υ: 3399, 2935, 2829, 1664, 1608, 1501, 1463, 1400, 1308, 1249, 1211, 1044, 928, 879, 771, 701 cm^{-1}; MS (m/z): HRMS (ESI) Calcd. for $C_{171}H_{198}N_4O_{36}Na$ ($[M+Na]^+$): 2907.3717, found: 2907.3667.

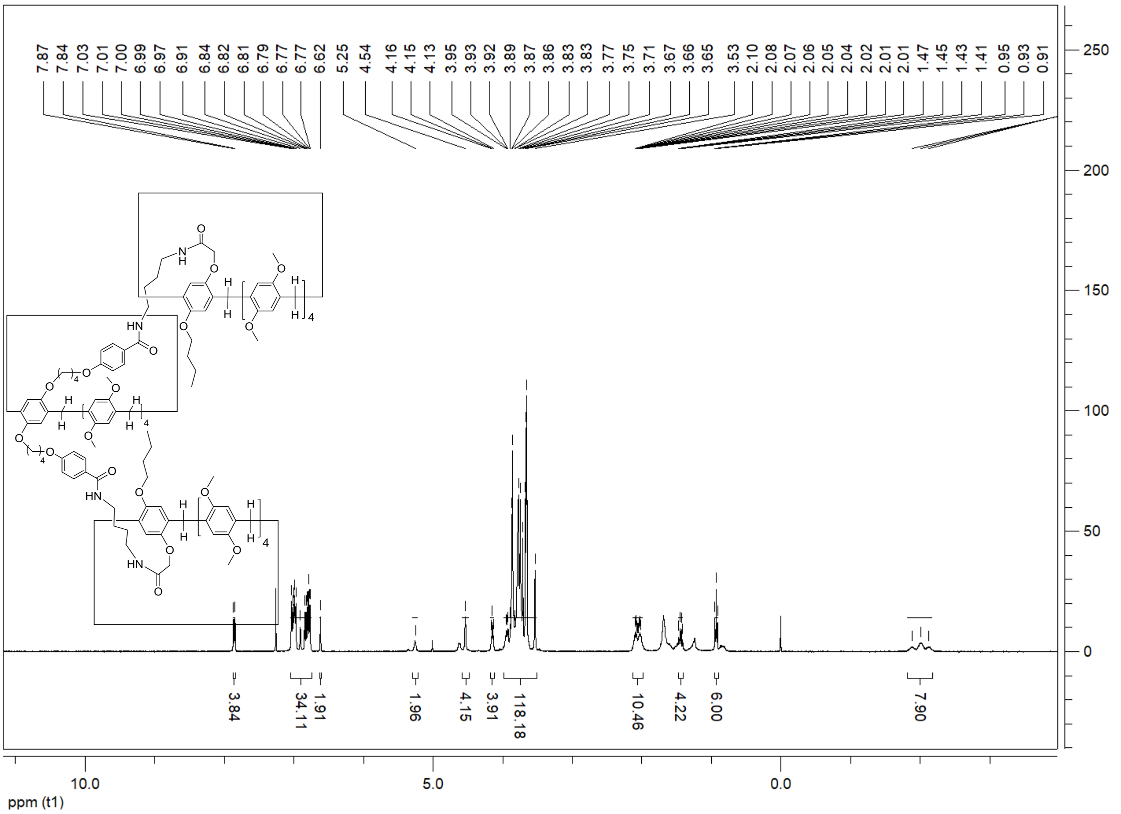

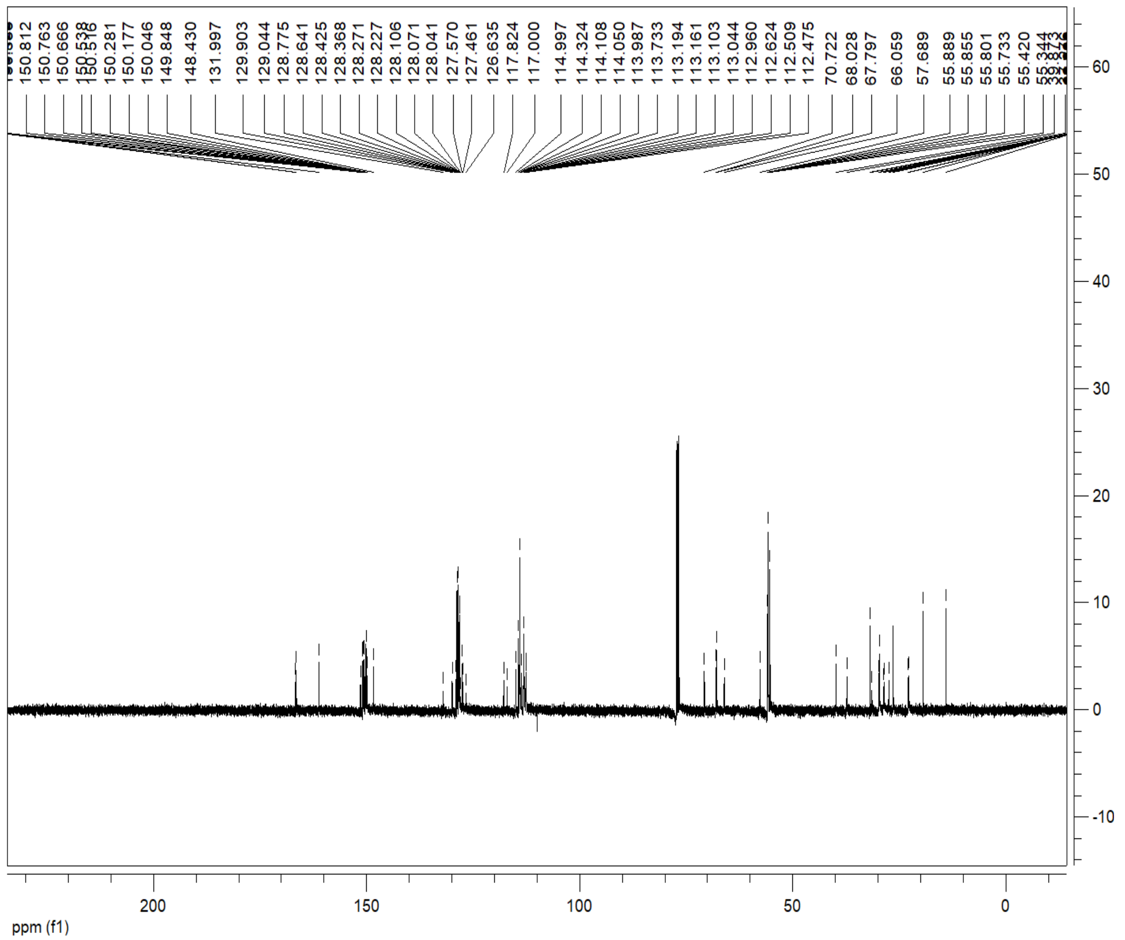

ppm (f1)

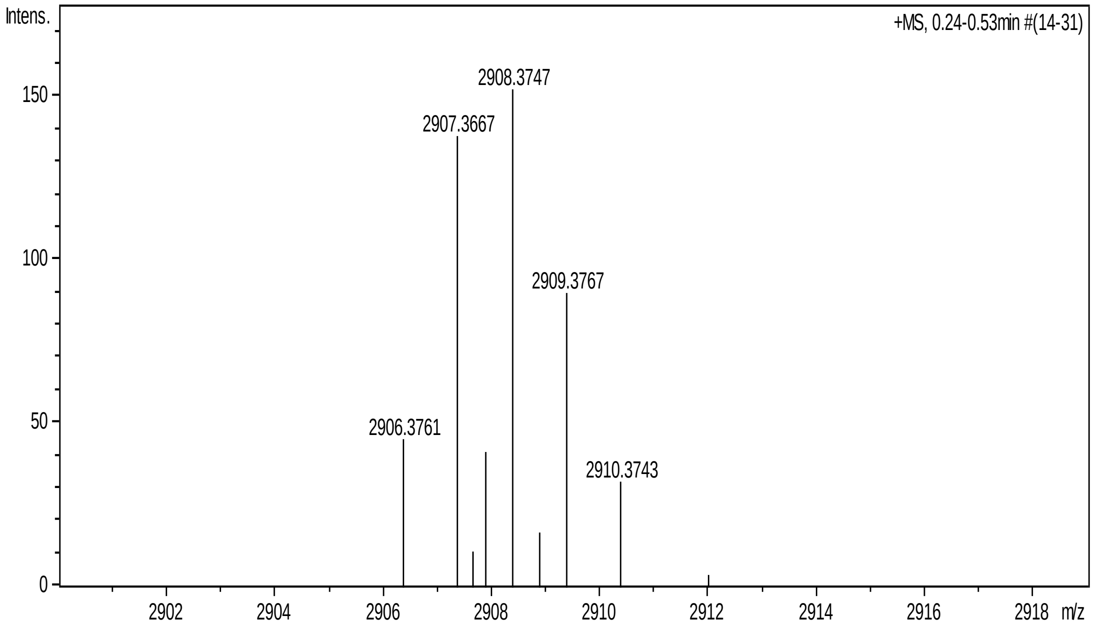

Intens.
+MS, 0.24-0.53min #(14-31)
2908.3747
2907.3667
2909.3767
2906.3761
2910.3743
m/z

9d: White solid, 17%, m.p. 140-142 °C; ^{1}H NMR (400 MHz, $CDCl_3$) δ: 7.84 (d, J = 4 Hz, 4H, ArH), 7.01-6.74 (m, 34H, ArH), 6.14 (s, 2H, NH), 5.28 (s, 2H, NH), 4.58 (s, 4H, CH_2), 4.11 (t, J = 4 Hz, 4H, CH_2), 3.91-3.62 (m, 118H, 46CH_2, 72OCH_3), 2.70 (brs, 2H, CH_2), 2.51 (brs, 2H, CH_2), 2.08-1.95 (m, 8H, CH_2), 1.48-1.42 (m, 4H, CH_2), 0.89 (t, J = 4 Hz, 6H, CH_3), -0.29 (brs, 4H, CH_2), -0.74 (brs, 2H, CH_2), -0.97 (brs, 2H, CH_2), -1.62 (brs, 4H, CH_2), -2.08 (brs, 4H, CH_2); ^{13}C NMR (100 MHz, $CDCl_3$) δ:167.6, 166.4, 161.4, 150.9, 150.9, 150.8, 150.8, 150.7, 150.7, 150.6, 150.6, 150.5, 150.4, 150.3, 150.3, 150.2, 150.1, 149.9, 147.2, 129.6, 129.3, 128.8, 128.5, 128.5, 128.4, 128.4, 128.3, 128.2, 128.2, 128.2, 128.1, 128.0, 127.9, 127.8, 127.5, 127.1, 115.8, 115.1, 115.1, 115.0, 114.8, 114.3, 114.3, 114.1, 114.1, 114.0, 113.9, 113.9, 112.8, 112.7, 112.7, 112.2, 112.2, 112.1, 109.9, 68.8, 67.9, 67.7, 65.8, 57.0, 56.2, 55.9, 55.8, 55.8, 55.7, 55.5, 55.5, 55.4, 55.3, 55.0, 53.1, 40.0, 37.9, 31.8, 30.1, 29.7, 29.6, 29.6, 29.5, 29.5, 29.3, 28.8, 28.6, 28.5, 26.4, 26.3, 26.2, 26.1, 24.1, 23.4, 23.3, 19.4, 13.9; IR (KBr) υ: 3409, 2935, 2856, 1673, 1608, 1500, 1464, 1400, 1307, 1249, 1210, 1100, 1045, 928, 879, 850, 771, 702, 647, 454 cm^{-1}; MS (m/z): HRMS (ESI) Calcd. for $C_{175}H_{206}N_4O_{36}Na$ ($[M+Na]^+$): 2963.4343, found: 2963.4339.

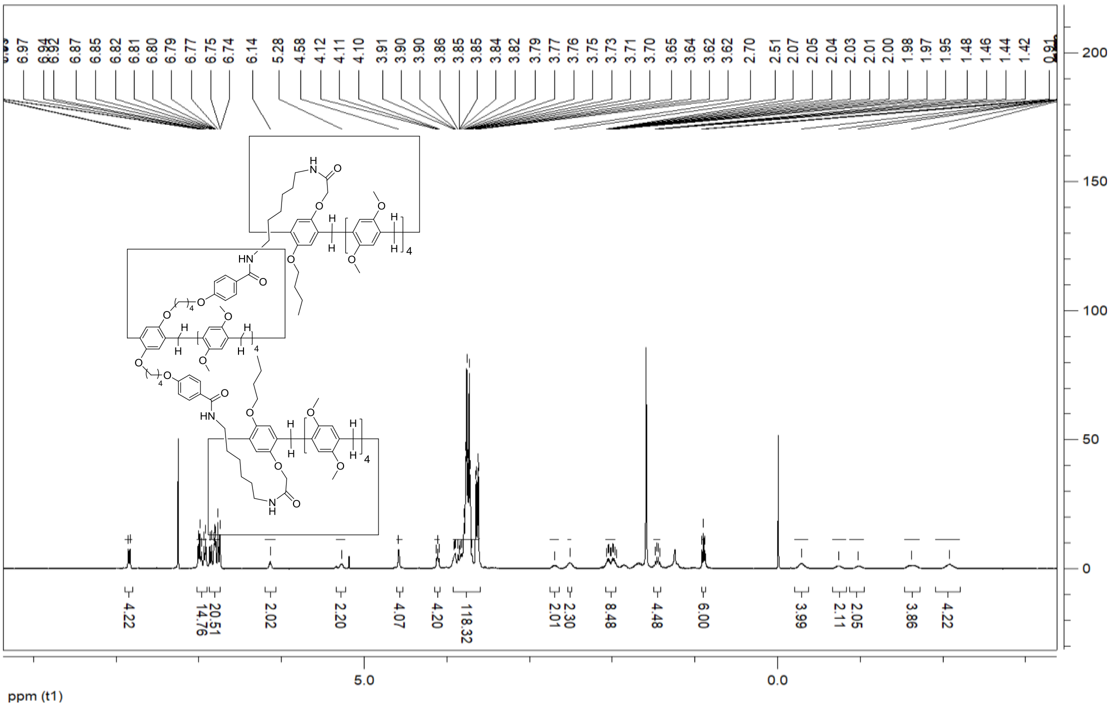

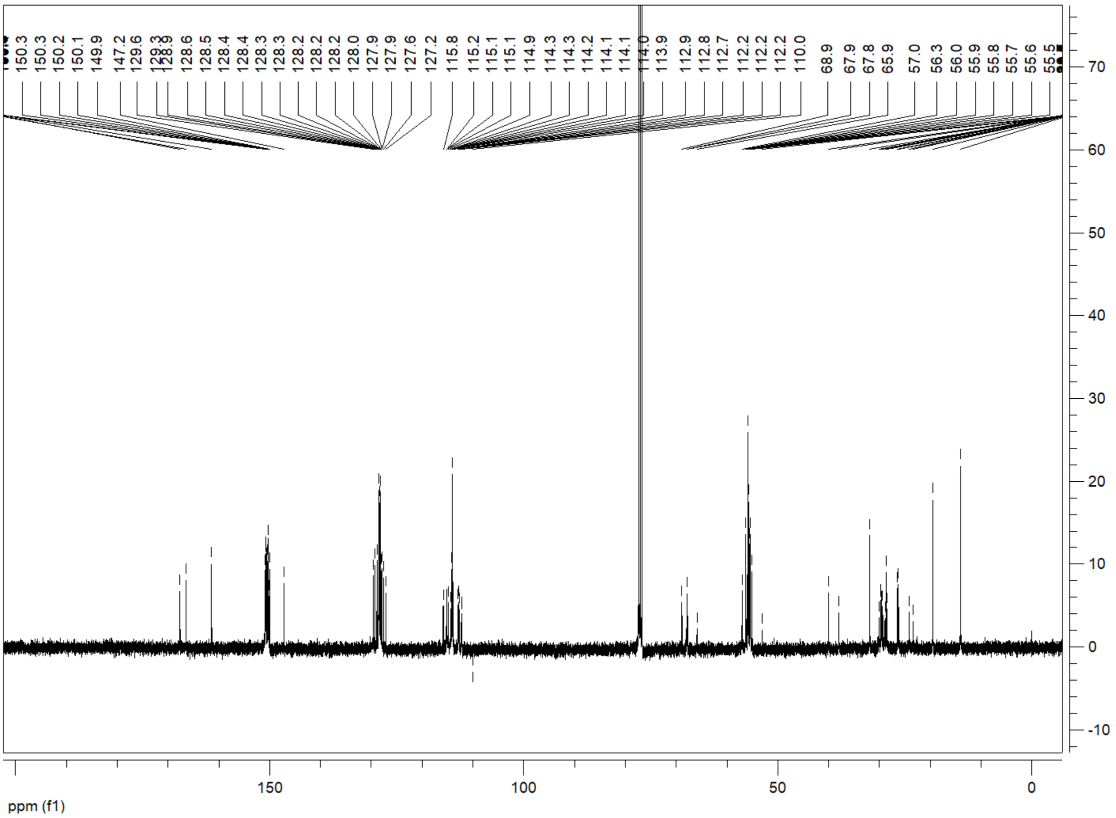
150.3
150.3
150.2
150.1
149.9
147.2
129.6
129.3
128.9
128.6
128.5
128.4
128.3
128.3
128.2
128.2
128.2
128.0
127.9
127.9
127.6
127.2
115.8
115.2
115.1
115.1
114.9
114.3
114.3
114.2
114.1
114.1
114.0
113.9
112.9
112.8
112.7
112.2
112.2
112.2
110.0
68.9
67.9
67.8
65.9
57.0
56.3
56.0
55.9
55.8
55.7
55.6
55.5
70
60
50
40
30
20
10
0
-10
150
100
50
0
ppm (f1)

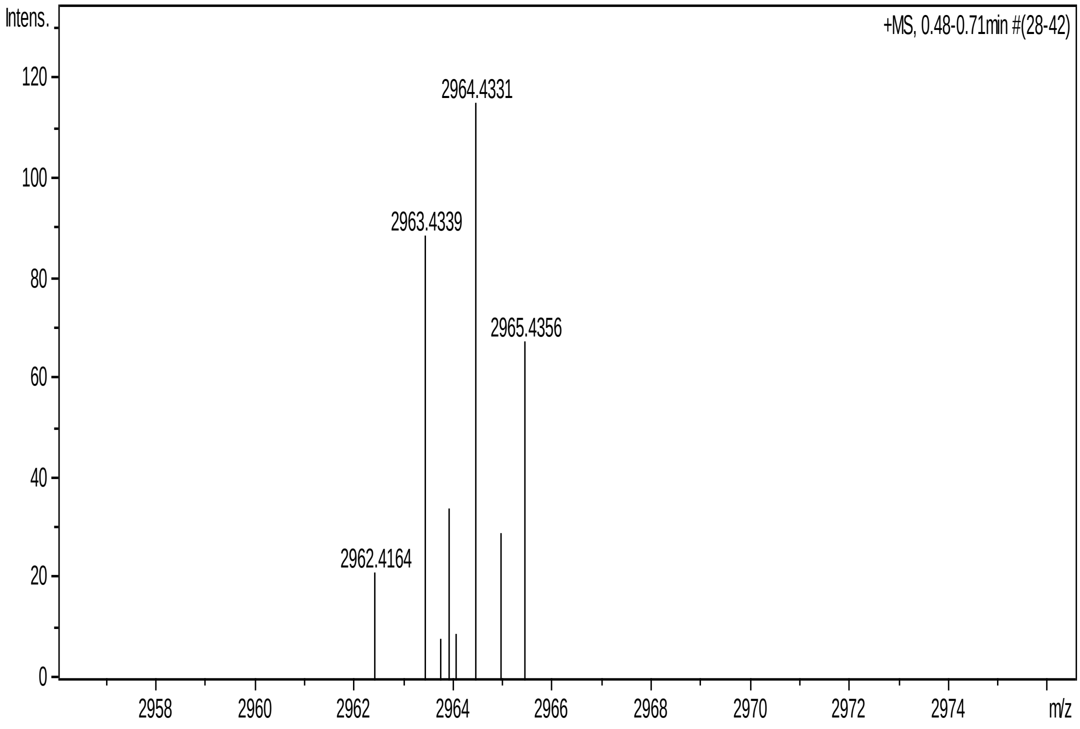
Intens.
+MS, 0.48-0.71min #(28-42)
2964.4331
2963.4339
2965.4356
2962.4164
120
100
80
60
40
20
0
2958
2960
2962
2964
2966
2968
2970
2972
2974
m/z
